# A ferroelectric-induced few-nanometer-thick graphene membrane enables molecular sliding for ultra-fast separation

**DOI:** 10.1093/nsr/nwag419

**Published:** 2026-07-09

**Authors:** Wenqi Ji, Yanan Guo, Guozhen Liu, Binyu Mo, Huan Yu, Kaihua Gao, Jiasheng Wu, Yangyang Mao, Huimin Chen, Gongping Liu, Young Moo Lee, Wanqin Jin, Nanping Xu

**Affiliations:** State Key Laboratory of Materials-Oriented Chemical Engineering, College of Chemical Engineering, Nanjing Tech University, Nanjing 211800, China; State Key Laboratory of Materials-Oriented Chemical Engineering, College of Chemical Engineering, Nanjing Tech University, Nanjing 211800, China; State Key Laboratory of Materials-Oriented Chemical Engineering, College of Chemical Engineering, Nanjing Tech University, Nanjing 211800, China; State Key Laboratory of Materials-Oriented Chemical Engineering, College of Chemical Engineering, Nanjing Tech University, Nanjing 211800, China; State Key Laboratory of Materials-Oriented Chemical Engineering, College of Chemical Engineering, Nanjing Tech University, Nanjing 211800, China; State Key Laboratory of Materials-Oriented Chemical Engineering, College of Chemical Engineering, Nanjing Tech University, Nanjing 211800, China; State Key Laboratory of Materials-Oriented Chemical Engineering, College of Chemical Engineering, Nanjing Tech University, Nanjing 211800, China; State Key Laboratory of Materials-Oriented Chemical Engineering, College of Chemical Engineering, Nanjing Tech University, Nanjing 211800, China; State Key Laboratory of Materials-Oriented Chemical Engineering, College of Chemical Engineering, Nanjing Tech University, Nanjing 211800, China; State Key Laboratory of Materials-Oriented Chemical Engineering, College of Chemical Engineering, Nanjing Tech University, Nanjing 211800, China; Suzhou Laboratory, Suzhou 215100, China; State Key Laboratory of Materials-Oriented Chemical Engineering, College of Chemical Engineering, Nanjing Tech University, Nanjing 211800, China; Department of Energy Engineering, College of Engineering, Hanyang University, Seoul 04763, Republic of Korea; State Key Laboratory of Materials-Oriented Chemical Engineering, College of Chemical Engineering, Nanjing Tech University, Nanjing 211800, China; State Key Laboratory of Materials-Oriented Chemical Engineering, College of Chemical Engineering, Nanjing Tech University, Nanjing 211800, China; Suzhou Laboratory, Suzhou 215100, China

**Keywords:** graphene membrane, ferroelectric-polarization-assisted, phase transition, molecular sliding, water desalination

## Abstract

Graphene’s atomically smooth surface offers a promising platform for achieving superlubricity, not only in solid-state contacts but also in mass transport. While its ability to minimize interfacial friction has been extensively explored in microelectromechanical systems, engines and biomedical implants, harnessing this property for near-frictionless molecular flow to enable ultra-fast permeation remains a fundamental challenge. The principal limitation lies in the fabrication of defect-free graphene membranes with an exposed surface for probing intrinsic molecular friction and the associated transport phenomena. Here, we demonstrate a ferroelectric-polarization-assisted strategy for fabricating a 3.5-nm-thick graphene skin membrane for ultra-fast and selective separation. This is achieved by electrospinning a graphene oxide (GO)-poly(vinylidene fluoride) (PVDF) mixture, followed by thermal reduction. The crucial step involves the electric-field-induced transition of PVDF from the paraelectric (*α*) to the ferroelectric (*β*) phase, which promotes ordered chain packing and reduces the polymer’s affinity for GO, thereby driving the spontaneous segregation of GO to the PVDF nanofiber surface. The resulting graphene surface minimizes interfacial interactions with permeating molecules, reducing the molecular flow friction coefficient by 2–3 orders of magnitude. Acting either as a selective layer or a support, the membrane exhibits ultra-fast permeation of water and organic solvents, surpassing current state-of-the-art membranes for water and solvent purification. For instance, a water flux up to 105.1 L m^−^^2^ h^−^^1^, a salt rejection of >99.99% and long-term stability of 1000 h are achieved for desalination of hypersaline with salt concentrations >150 g L^−^^1^. This work establishes a new paradigm for realizing ultra-fast molecular transport and provides a versatile platform for investigating nanoscale fluidics at material interfaces.

## Teaser

An ultrathin, highly porous graphene membrane possesses an atomically smooth surface to minimize interfacial friction, enabling near-frictionless molecular sliding for ultra-fast and stable separation.

## INTRODUCTION

Graphene, with its atomic-scale thickness and exceptional smoothness, provides an ideal platform for low-resistance mass transport [1,2], offering a promising avenue to address the challenges in molecular-scale separations such as hypersaline water treatment and organic solvent purification. However, the use of membranes in these processes remains constrained by trade-offs between permeability and selectivity, or between permeability and structural stability [3–6]. Conventional approaches to graphene-based membrane design typically exploit the atomic thickness of the lattice to minimize transport resistance while tailoring the tunable interlayer galleries for size-exclusive selectivity [7]. Nanoporous few-layer graphene membranes, for instance, markedly reduce transmembrane mass transport resistance, leading to enhanced permeation rates [8–13]. Yet, their practical deployment is hampered by low pore density, complex perforation procedures and restricted membrane dimensions. Precisely controlling membrane pore dimensions at the sub-nanometer scale facilitates fast and selective transport [14]. Sub-nanometer interlayer channels within stacked two-dimensional membranes, particularly laminar graphene oxide (GO) membranes, enable precise molecular sieving, thereby achieving highly selective transport of small molecules and ions [15–19]. Nonetheless, their permeability remains inadequate, primarily due to tortuous transport pathways and low porosity.

Alongside efforts to exploit graphene’s atomic thinness and molecular sieving capability, an alternative strategy is to harness its atomically smooth surface to enable near-frictionless molecular flow—a mechanism that could transcend the intrinsic limitations of conventional membrane materials. To date, most research has focused on utilizing graphene’s superlubricious surfaces to minimize solid–solid friction, with broad applications in micro-/nanoscale devices, engines and biomedical implants [20–23]. In contrast, whether fluids—particularly molecules flowing over a graphene surface—can experience similarly ultra-low transport resistance remains an open question. A major bottleneck in addressing this lies in fabricating defect-free, highly porous graphene membranes suitable for effective molecular separation. Existing strategies that attempt to exploit the surface properties of graphene (rather than GO) primarily rely on its incorporation as a filler within a polymer matrix [24]. In such composites, graphene typically constitutes only a minor component, with most nanosheets dispersed throughout the polymer bulk. Consequently, the graphene surfaces are obscured by polymer chains, preventing them from presenting the desired atomically flat surface to the fluid phase. Creating a continuous, defect-free graphene skin—fully exposed and free-standing on the membrane surface—therefore represents a critical objective. Such a configuration is essential for probing fluid transport behavior at the graphene surface and for harnessing graphene’s superlubricity to achieve ultra-low-resistance molecular separation.

It is well known that when separating iron and wood chips using a magnetic field, the iron chips are uniformly attracted to the magnet, whereas the wood chips are displaced toward the periphery. This directional segregation behavior is analogous to the phase transition mechanism observed in ferroelectric polymers such as poly(vinylidene fluoride) (PVDF), in which the application of an electric field induces the rearrangement of polymer chains [25–27], thereby altering their packing state and interaction energy with neighboring materials. Inspired by this analogy, we hypothesized that the electric-field-induced chain rearrangement in ferroelectric polymers could be harnessed to drive the migration of graphene nanosheets toward the polymer surface during phase separation in a graphene–polymer mixture, yielding a continuous graphene-skinned membrane.

**Figure 1.**
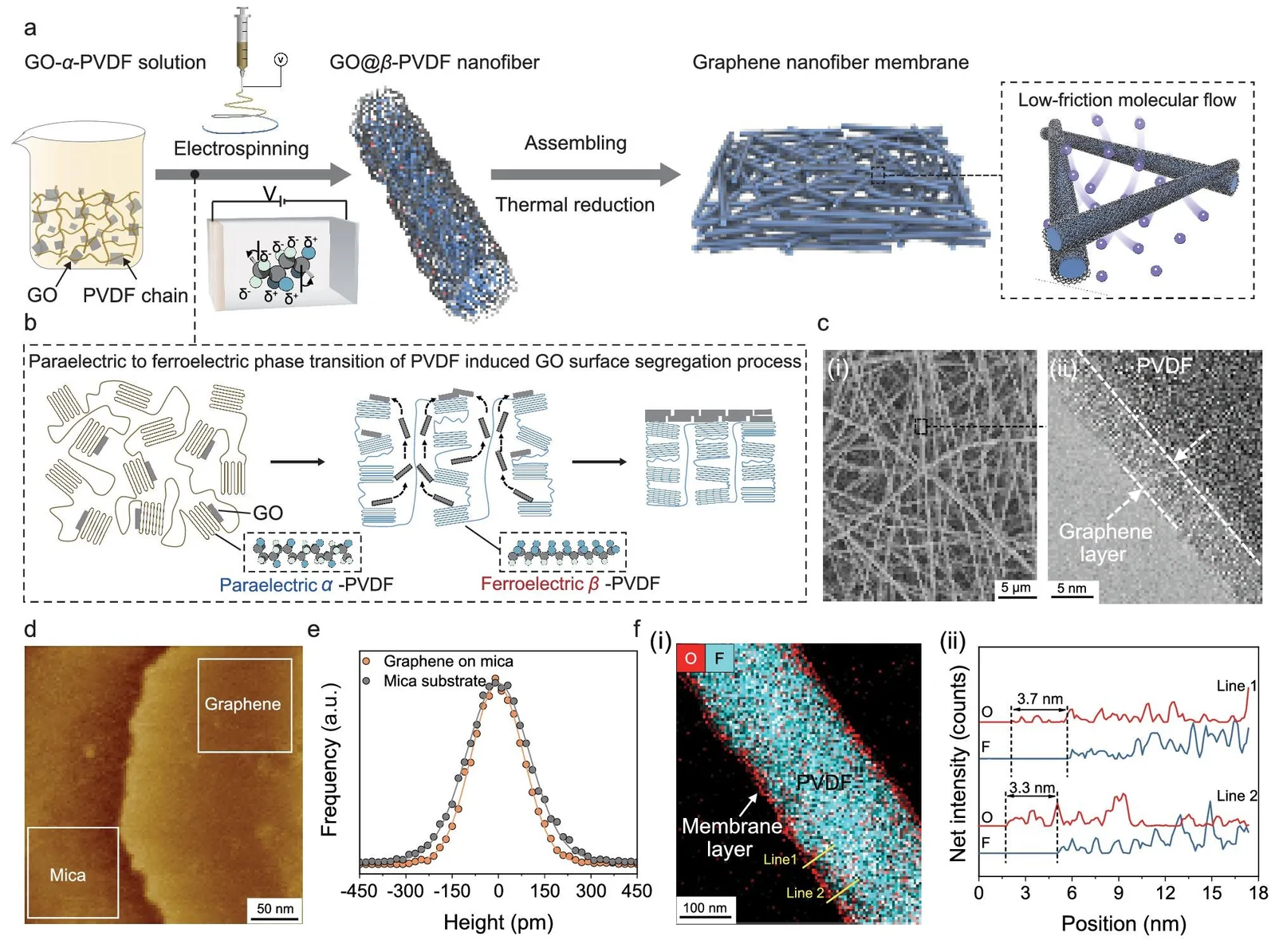
Membrane fabrication and characterization. (a) Electrospinning of GO-*α*-PVDF solution into GO@*β*-PVDF nanofibers, which are assembled into a nanofiber membrane and then thermally reduced to form an ultra-thin and porous graphene nanofiber membrane. The atomically smooth graphene layer exhibits ultra-fast molecular transport. (b) During electrospinning, PVDF undergoes a phase transition from the paraelectric (*α*-PVDF) to the ferroelectric (*β*-PVDF) phase under the applied electric field. Compared with *α*-PVDF, *β*-PVDF exhibits more ordered chain packing and weaker molecular interaction with GO, thereby promoting the segregation of GO to the PVDF surface. (c) Scanning electron microscopy (SEM) and transmission electron microscopy (TEM) images of the graphene nanofiber membrane. (d) Atomic force microscopy (AFM) image showing the boundary between the graphene monolayer (right) and the mica substrate (left). (e) Height histograms of graphene on mica and the mica substrate, obtained from the respective white squares in panel (d). (f-i) EDX mapping of a nanofiber from GO@PVDF membranes fabricated with a GO concentration of 3 mg mL^−^^1^ under an electric field of 15 kV. (f-ii) Line-scan elemental distribution of O and F along lines 1 and 2 in panel (i).

Herein, we report a ferroelectric-polarization-assisted strategy for fabricating an ultra-thin graphene membrane (Fig. 1a). The membrane was prepared by electrospinning a mixture of GO and *α*-phase poly(vinylidene fluoride) (*α*-PVDF), followed by thermal reduction. During electrospinning, the *α*-PVDF (paraelectric phase) undergoes a field-induced transition to the *β*-phase (ferroelectric phase). This *α*-to-*β* phase transition involves a conformational change in the polymer chains—from a structure in which H and F atoms alternate on both sides of the chain in *α*-PVDF, to an all-trans conformation in *β*-PVDF where all H or F atoms are aligned on the same side. The enhanced chain ordering and weakened molecular interaction with GO drive the migration of GO nanosheets toward the fiber surface, resulting in the formation of a continuous skin (Fig. 1b and Fig. S1). Subsequent assembly of GO@*β*-PVDF nanofibers and thermal treatment—which reduces GO to graphene by removing oxygen‑containing groups—results in the formation of a graphene nanofiber membrane. Notably, this graphene skin layer can function either as a selective separation interface or as an underlying substrate, showing a molecular-sliding effect that enables ultra-fast transport of water and organic solvent molecules during separation processes.

## RESULTS AND DISCUSSION

### Membrane fabrication

**Figure 2.**
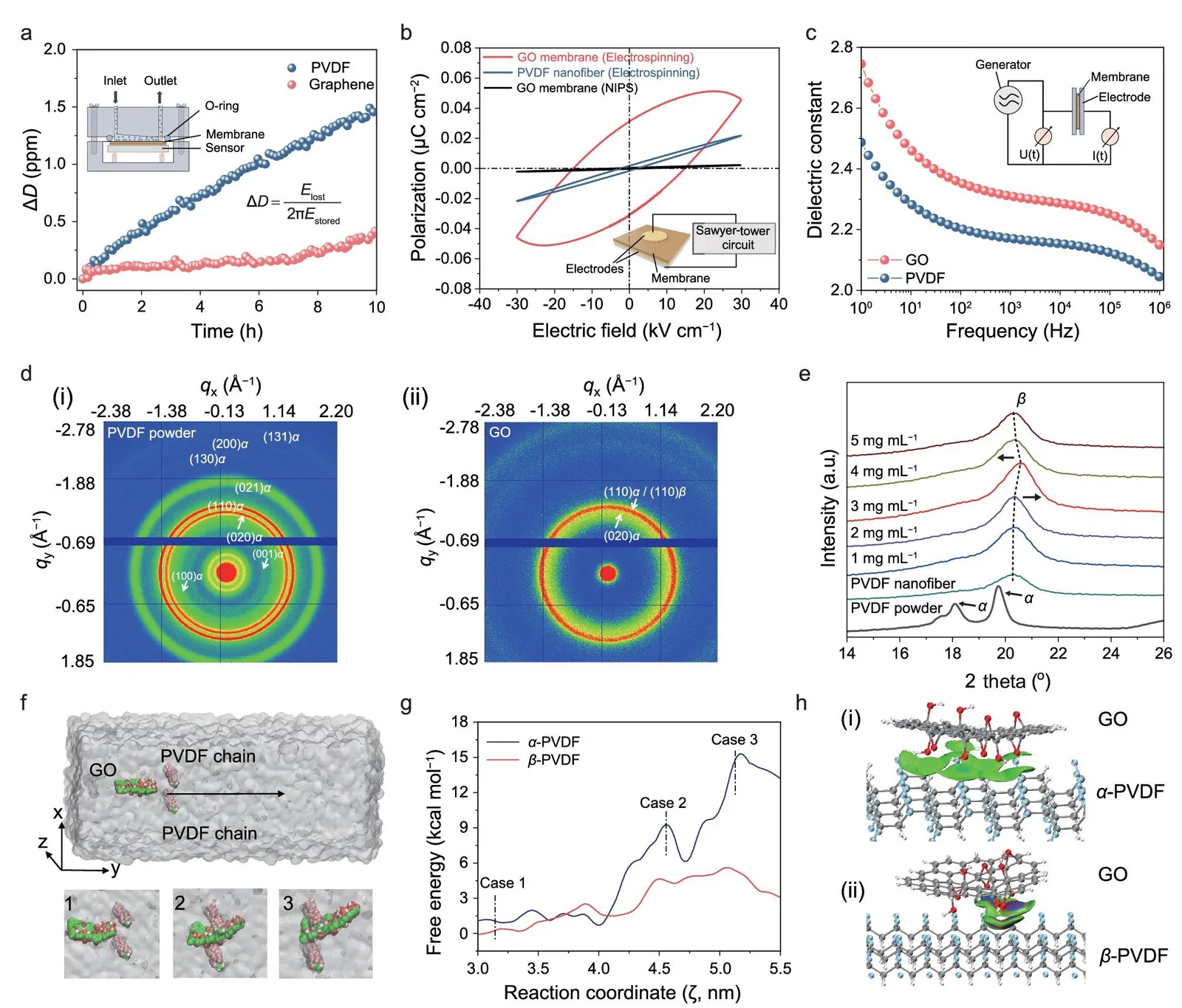
Membrane formation mechanism. (a) Energy dissipation shift (Δ*D*) measured by QCM-D for pristine PVDF nanofiber and graphene nanofiber membranes, highlighting differences in polymer chain flexibility. (b) Ferroelectric hysteresis loops of GO membranes prepared via NIPS and electrospinning: PVDF and GO membranes. (c) Dielectric spectra of PVDF and GO membranes prepared by electrospinning. (d) 2D-WAXS profiles of (i) PVDF powder and (ii) GO membrane with a GO concentration of 3 mg mL^−^^1^. (e) XRD patterns of PVDF and GO membranes with different GO concentrations. Molecular simulations of the energy barrier of GO inside and outside of PVDF chains: (f and g) representative configurations and corresponding potential of mean force (PMF) curves for GO transfer across PVDF chains. (h) The IGMH isosurfaces (isovalue = 0.001 a.u.) depicting interactions between GO and (i) *α*-PVDF and (ii) *β*-PVDF (–CF_2_ groups facing GO). Color scheme: C atoms in gray, O atoms in red, F atoms in light cyan and H atoms in white.

We prepared the graphene membrane by thermal reduction of a GO precursor, because pristine graphene lacks functional groups and readily aggregates in solution, making it unsuitable for forming continuous films (Fig. S2). The precursor solution for membrane fabrication was obtained by dispersing GO nanosheets (lateral size: 1–2 μm; thickness: ∼1 nm; Fig. S3) and *α*-PVDF in a mixed solvent of *N,N*-dimethylformamide and acetone (Fig. S2). Owing to favorable molecular interactions between GO and PVDF, as confirmed by molecular simulations (Fig. 2h), a uniform and processable solution was achieved. This solution was processed into a nanofibrous mat via an electrospinning process, producing a GO@PVDF nanofiber membrane with a highly interconnected porous architecture (porosity: ∼85%; Figs S4–S6). Notably, the porosity is one order of magnitude higher than those of conventional graphene-based membranes, which have a porosity of 4%–12% [9,10,12,28], offering a structural foundation for low-resistance permeation. Systematic optimization of the electrospinning parameters, particularly the GO concentration and applied voltage, proved critical for forming a continuous GO skin layer.

Elemental mapping by energy-dispersive X-ray spectroscopy (EDX) coupled with transmission electron microscopy (TEM) provided direct evidence of the nanostructural evolution. At an optimal GO concentration of 3 mg mL^−^^1^, a continuous oxygen-rich layer—originating exclusively from GO—was uniformly observed enveloping the fluorine-rich PVDF nanofiber core (Fig. 1f-i and Fig. S7). Linear EDX scans across multiple edge positions revealed a consistent GO layer thickness of ∼3.5 nm (Fig. 1f-ii), which aligns closely with the theoretically predicted value of 3.4 nm for a continuous, conformal GO shell encapsulating a PVDF nanofiber core (Fig. S8; see Supplementary data for details). This finding confirms the complete segregation of GO onto the nanofiber surface, forming a uniform and defect-free GO coating. Thermal reduction of the as-spun GO membranes yielded a graphene layer (Fig. S9). TEM revealed that the graphene layer maintained a thickness comparable to that of the initial GO layer (Fig. 1c and Fig. S10). Atomic force microscopy (AFM) further demonstrated that the graphene nanosheets obtained by thermal reduction of GO exhibited a surface roughness similar to that of the atomically flat mica standard [1], directly evidencing the smooth surface of the graphene layer (Fig. 1d and e). While the reduction process inevitably introduces minor oxygen residues and topological defects, the resulting reduced GO exhibits ultra-low oxygen content and structural integrity comparable to that of reported chemical vapor deposition (CVD)-grown graphene [29–31] (Figs S11–S13).

To delineate the parameter space of this assembly process, we systematically varied the GO concentration. Reducing the GO concentration to 1–2 mg mL^−^^1^ yielded a thinner layer of 1.5–2.5 nm due to the limited amount of nanosheets (Figs S14 and S15). In contrast, concentrations exceeding the optimum (4–5 mg mL^−^^1^) generated a discontinuous, non-conformal coating whose measured thickness deviated markedly from theoretical predictions (Figs S8 and S16–S18). This was attributed to the nanosheet agglomeration within the PVDF bulk phase, with only a portion of nanosheet segregating to the PVDF surface. The electrospinning voltage was found to be equally decisive. An applied voltage of 15 kV afforded a defect-free, ultra-thin GO layer. Lower voltages (e.g. 10 kV) generated insufficient electric field strength to fully align PVDF chains and drive complete GO segregation, yielding a suboptimal layer (Fig. S19). Conversely, excessively high fields (e.g. 20 kV) caused violent jet thinning and instability, displacing GO nanosheets into the surrounding air and leading to incomplete surface coverage (Fig. S20).

To confirm the complete surface localization of the graphene layer, X-ray photoelectron spectroscopy depth profiling was performed across the GO@PVDF nanofiber (Fig. S21). The analysis reveals a stark compositional divide: the oxygen atomic percentage decreased by an order of magnitude within the first 10 nm from the surface, beyond which it remained nearly constant, confirming that the graphene layer is confined to the surface region. The asymmetric graphene@PVDF architecture was further examined using a quartz crystal microbalance with a dissipation monitoring (QCM-D) system (Fig. S22). Analysis of the dissipation shift (Δ*D*), which reflects viscoelastic damping and thus polymer chain mobility, revealed fundamentally distinct behaviors. The pristine PVDF exhibited a sharp increase in Δ*D*, indicating highly mobile polymer chains, whereas the graphene-coated PVDF displayed a markedly suppressed response (Fig. 2a). This signifies that the conformal graphene layer exerts nanoscale confinement, effectively restricting large-scale chain dynamics in the underlying PVDF. This confinement was further corroborated by structural and mechanical analyses. X-ray diffraction (XRD) and small-angle X-ray scattering (SAXS) spectra both showed reduced inter-chain and lamellar spacing (Fig. 2e and Figs S23 and S24), suggesting that the graphene skin compacts the polymer matrix. Correspondingly, tensile testing revealed a significant enhancement in mechanical strength (Fig. S25). These findings demonstrate that the GO segregates to form a seamless overlayer that fully encapsulates the PVDF nanofiber, producing a uniform and continuous graphene skin.

### Membrane formation mechanism

The consistent formation of a continuous skin layer raises a fundamental question—what underlying principle drives such complete GO segregation? Attempts to replicate this structure using non-solvent-induced phase separation (NIPS) or by electrospinning GO with non-ferroelectric polymers failed to yield a surface-segregated GO layer; instead, the GO nanosheets remained embedded within the polymer matrix (Figs S26–S30). We therefore hypothesized that the ferroelectric behavior of PVDF during electrospinning is the necessary prerequisite for complete GO segregation. To elucidate this mechanism, we first examined the ferroelectric properties of the GO@PVDF system using polarization–electric field (P–E) hysteresis measurements (Fig. S31), which revealed a pronounced electromechanical transition. PVDF powders exhibited a linear P–E loop, indicative of a dominant paraelectric phase (*α*-phase), in which the helical chain conformation causes the C–F dipoles to cancel spatially. In contrast, the electrospun PVDF nanofiber and GO@PVDF nanofiber membranes displayed a distinct non-linear hysteresis loop (Fig. 2b), characteristic of the ferroelectric *β*-phase [27,32]. The phase transformation was further confirmed at the molecular level. Two-dimensional wide-angle X-ray scattering (2D-WAXS) profiles of PVDF powder showed characteristic *α*-phase diffraction rings—(100), (001), (020), (110), (021), (130), (200) and (131) [33]. In comparison, these *α*-phase diffraction rings largely disappeared in the GO@PVDF nanofiber membrane, while a new *β*-phase diffraction ring corresponding to the (110) plane emerged (Fig. 2d and Fig. S32). Complementary infrared spectroscopy further corroborated the conformational transition from the *α*- to the *β*-phase (Fig. S33).

We attribute this phase transformation to the high voltage applied during electrospinning, which exerts sufficient torque on the C–F dipoles of PVDF chains to induce collective polarization and realignment [27]. This process enforces a conformational transition from the disordered *α*-phase to the highly ordered *β*-phase (Fig. 1b) [26], accompanied by a loss of conformational entropy. To compensate for this entropy reduction, the system expels GO nanosheets from the polymer bulk, thereby increasing translational entropy and lowering overall free energy. The surface-segregated GO layer, in turn, further promotes the *α*–*β* phase transition through interfacial confinement, which stabilizes the polar *β* phase and reinforces dipole alignment. This synergistic effect is reflected in the enhanced remanent polarization—a key indicator of ferroelectric behavior—and the increased dielectric permittivity (Figs S31 and S34). These results also confirm the complete segregation of GO from the PVDF bulk phase, as any residual GO within the polymer matrix would disrupt ordering and diminish the observed ferroelectric response.

Our molecular dynamics simulations provide kinetic insight into the surface segregation mechanism of GO nanosheets during electrospinning. The energy for GO outside the PVDF matrix is substantially lower than that within the polymer bulk (∆*E* = −34.8 kcal mol^−^^1^) (Figs S35 and S36), indicating that surface localization is thermodynamically favorable. However, the migration of GO toward the PVDF surface must overcome an energy barrier associated with diffusion through the polymer chains. Compared with *α*-PVDF, this energy barrier is ∼2-fold lower in *β*-PVDF (Fig. 2f and g and Fig. S37), suggesting that the *β*-phase facilitates GO migration. This difference arises from the distinct molecular interactions between GO and the two PVDF phases. Density functional theory (DFT) calculations show that the interaction energy between GO and *β*-PVDF (−0.036 kcal mol^−^^1^ Å^−^^2^ when –CF_2_ groups face GO) is roughly half that between GO and *α*-PVDF (−0.07 kcal mol^−^^1^ Å^−^^2^). The Independent Gradient Model based on Hirshfeld partition (IGMH) isosurfaces further clarifies the nature of these interactions (Fig. 2h and Fig. S38). The IGMH isosurface between *α*-PVDF and GO displays green and light blue regions, corresponding to O–H⋯F and O⋯C–H hydrogen bonds and van der Waals attractive interactions. In contrast, the IGMH isosurface between *β*-PVDF and GO exhibits pronounced red regions, signifying repulsive interactions between the negatively charged epoxy-O*^δ^*^⁻^ of GO and –F*^δ^*^⁻^ of *β*-PVDF. At the PVDF–GO interface, *α*-PVDF presents –CH_2_ and –CF_2_ groups, enabling mixed attractive interactions, whereas *β*-PVDF exposes predominantly –CF_2_ groups, inevitably introducing electrostatic repulsion with GO. Consequently, the overall interaction energy for *β*-PVDF is weaker than that for *α*-PVDF. Even when the –CH_2_ groups of *β*-PVDF face GO, the interaction energy (−0.056 kcal mol^−^^1^ Å^−^^2^; Fig. S38) remains lower than that of the GO–*α*-PVDF interface. Thus, the *α*–*β* phase transition effectively reduces the polymer–GO interaction strength, diminishing interfacial compatibility and promoting spontaneous migration of GO nanosheets to the PVDF surface. These results confirm that *β*-phase is essential for GO migration; together with the mechanism of reduced interaction energy and enhanced chain ordering following phase transition, they reveal that the *α*-to-*β* transition initiates prior to significant GO segregation and then the wrapping effect of GO also promotes the formation of *β*-phase.

### Molecular separation performance

**Figure 3.**
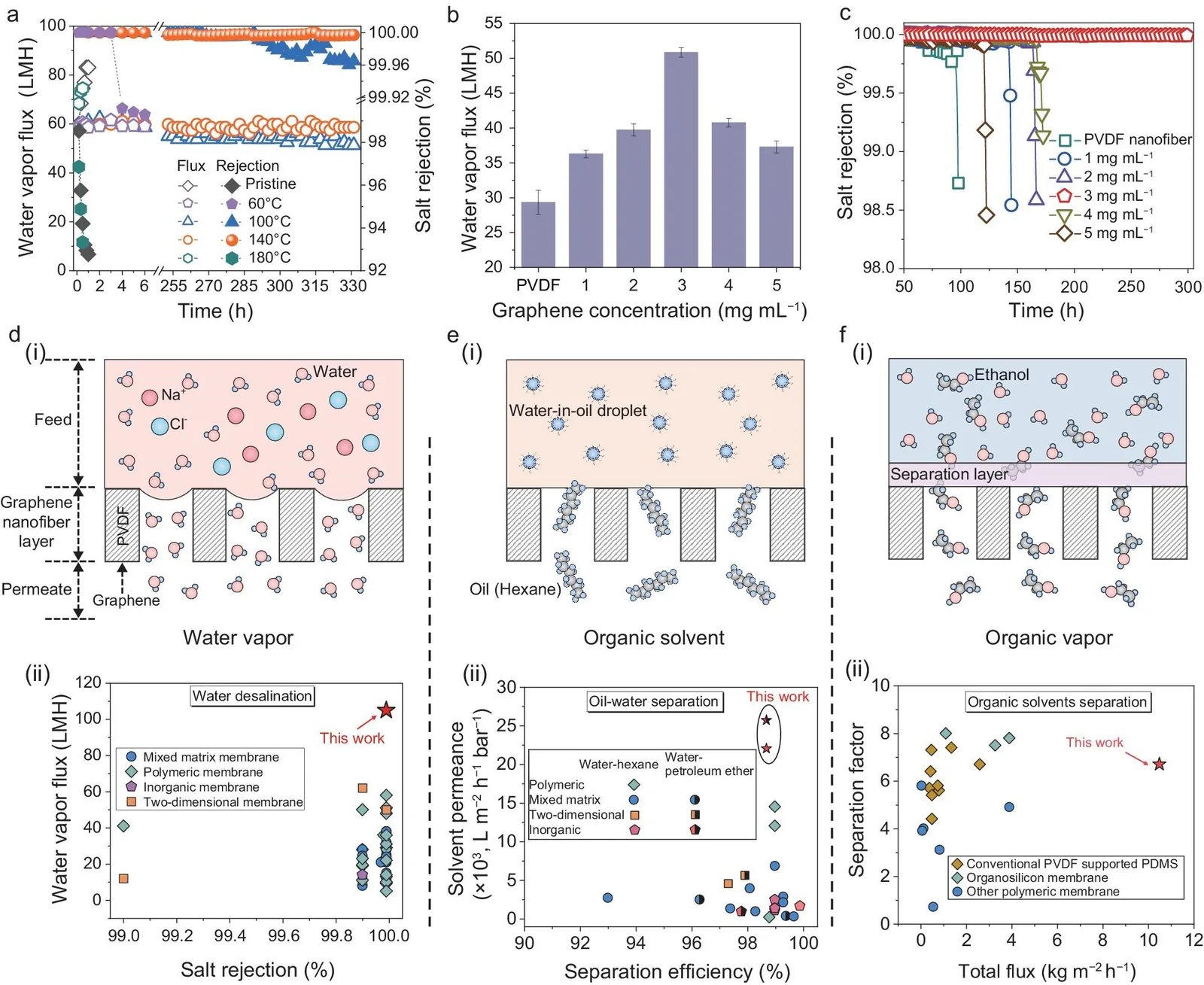
Membrane separation performance. (a) Effect of the thermal treatment temperature on the desalination performance. The feed temperature is 60°C and the NaCl concentration is 35 g L^−^^1^. (b) Water vapor flux and (c) salt rejection rate of graphene nanofiber membranes fabricated using different GO concentrations. The feed temperature is 60°C and the NaCl concentration is 150 g L^−^^1^. Schematic illustrations of the graphene nanofiber membrane applied to: (d-i) water desalination, (e-i) oil–water separation and (f-i) organic solvent separation using a graphene-supported polydimethylsiloxane (PDMS) separation layer. Panels (ii) are the corresponding comparison of separation performance with state-of-the-art membranes.

We first examined the water transport property of the graphene membrane using an interfacial evaporation desalination process [also known as direct contact membrane distillation (DCMD), Fig. S39]. In this process, a temperature gradient under non-isothermal conditions drives water vapor from a heated saline feed across a hydrophobic membrane (Fig. 3a–d) [34], wherein water vapor molecules permeate through the large pores between the graphene nanofibers (Fig. 3d-i). The graphene membrane desalination performance is closely related to the thermal reduction temperature and nanosheet concentration. We found that 140°C thermal reduction is sufficient to obtain a hydrophobic graphene surface with a water contact angle >140°, ensuring a stable flux and salt rejection during the desalination process (Fig. 3a and Fig. S40). As a sustainable alternative to vacuum membrane desalination or pervaporation desalination processes, the DCMD process can be more energy-efficient by potentially using waste heat or solar energy.

We tested the DCMD desalination performance of membranes fabricated using different nanosheet concentrations. A membrane with an orderly stacked and thus continuous graphene layer (sample: 3 mg mL^−^^1^) exhibited a water vapor flux of 50.9 L m^−^^2^ h^−^^1^ when exposed to high-salinity water containing 150 g L^−^^1^ NaCl (Figs 1a and 3b). The flux is higher than that of the membranes with a disorderly stacked and discontinuous graphene layer (samples: 0, 1, 2, 4 and 5 mg mL^−1^) (Fig. 3b and Figs S14–S17). It is worth noting that the pore size and porosity of the pristine PVDF and graphene membranes are similar (Fig. S6), thereby excluding the dominant influence of pore structure on the observed flux enhancement. This is ascribed to the intrinsically hydrophobic and atomically smooth graphene surface that facilitates the fast transport of water vapor with decreased resistance. Besides the low flux, current membrane distillation membranes generally suffer from the same wetting issue observed for membranes without a continuous graphene layer. The salt rejection rate starts to decay dramatically after operating for 70–170 h (Fig. 3c). Salt crystals formed on the membrane surface, as revealed by SEM images of the membranes after testing (Fig. S41). The influence of nanosheet size on the membrane desalination performance was also investigated. We found that the larger the lateral size, the easier nanosheets tend to agglomeration (Fig. S42a and b), which partially blocked the channels and increased the transport resistance for water vapor. Consequently, the water vapor flux decreased with increasing nanosheet lateral size (Fig. S42c). The membrane prepared from small-sized nanosheets maintained a stable salt rejection of >99.99% for over 300 h—an order of magnitude longer than the membranes made from larger nanosheets. This is because the agglomeration of larger nanosheets leads to membrane wetting by liquid water.

This graphene membrane exhibited an ultra-high water vapor flux of 105.1 L m^−^^2^ h^−^^1^ at 80°C, while maintaining a salt rejection above 99.99% (Figs S43 and S44). Both the intrinsic vapor permeance (normalized by partial vapor pressure) and the measured flux significantly surpass those of state-of-the-art polymeric membranes, including PVDF and polytetrafluoroethylene (PTFE) (Fig. 3d-ii, Fig. S45 and Tables S1 and S2). Besides, the membrane demonstrated exceptional selectivity toward boron—a hazardous contaminant in drinking water—achieving an exceptional rejection of 99.7%, far exceeding the 30%–50% removal typically reported for conventional reverse osmosis systems. Our membrane also achieved near-complete ion removal (∼99.99%) from simulated radioactive wastewater, demonstrating its strong potential for advanced environmental remediation and nuclear wastewater treatment (Table S3).

We further extend the applicability of the membrane to oily wastewater treatment (Fig. S46), a major environmental concern owing to the vast quantities discharged from industrial and domestic sources [35,36]. In this separation process, selective permeation is governed by the wettability contrast between water and organic phases, enabling one phase to pass through the membrane while the other is effectively repelled (Fig. 3e-i). When tested using a series of water-in-oil emulsions, the membrane exhibited solvent permeance of ∼22 000 L m^−^^2^ h^−^^1^ bar^−^^1^ for petroleum ether/water, ∼25 700 L m^−^^2^ h^−^^1^ bar^−^^1^ for hexane/water mixture and ∼18 530 L m^−^^2^ h^−^^1^ bar^−^^1^ for hexadecane/water mixtures, with permeate solvent purities consistently ∼99% (Figs S47 and S48). The obtained permeance values surpass those of most reported membranes by one to two orders of magnitude (Fig. 3e-ii and Table S4).

Beyond serving as a standalone separation layer, the graphene membrane was further exploited as a support for organic solvent recovery via the pervaporation process (Fig. S49). In this process, target components from the liquid feed adsorb into the selective layer, diffuse through it and subsequently desorb as a vapor on the permeate side before transporting through the support layer (Fig. 3f-i). PDMS—a benchmarked membrane material for solvent recovery—was deposited as a selective separation layer on top of the graphene membrane (Fig. S50). The resulting PDMS@graphene membrane was evaluated for ethanol recovery from ethanol–water mixtures, a key industrial process in biofuel production from fermentation broths [37]. As shown in Fig. 3f-ii, the composite membrane exhibited a total flux of 10.7 kg m^−^^2^ h^−^^1^, which is much higher than that of conventional substrate-supported PDMS membranes, while the separation factor remained similar. This separation performance substantially surpasses the performance of the state-of-the-art membranes (Fig. S51 and Table S5).

**Figure 4.**
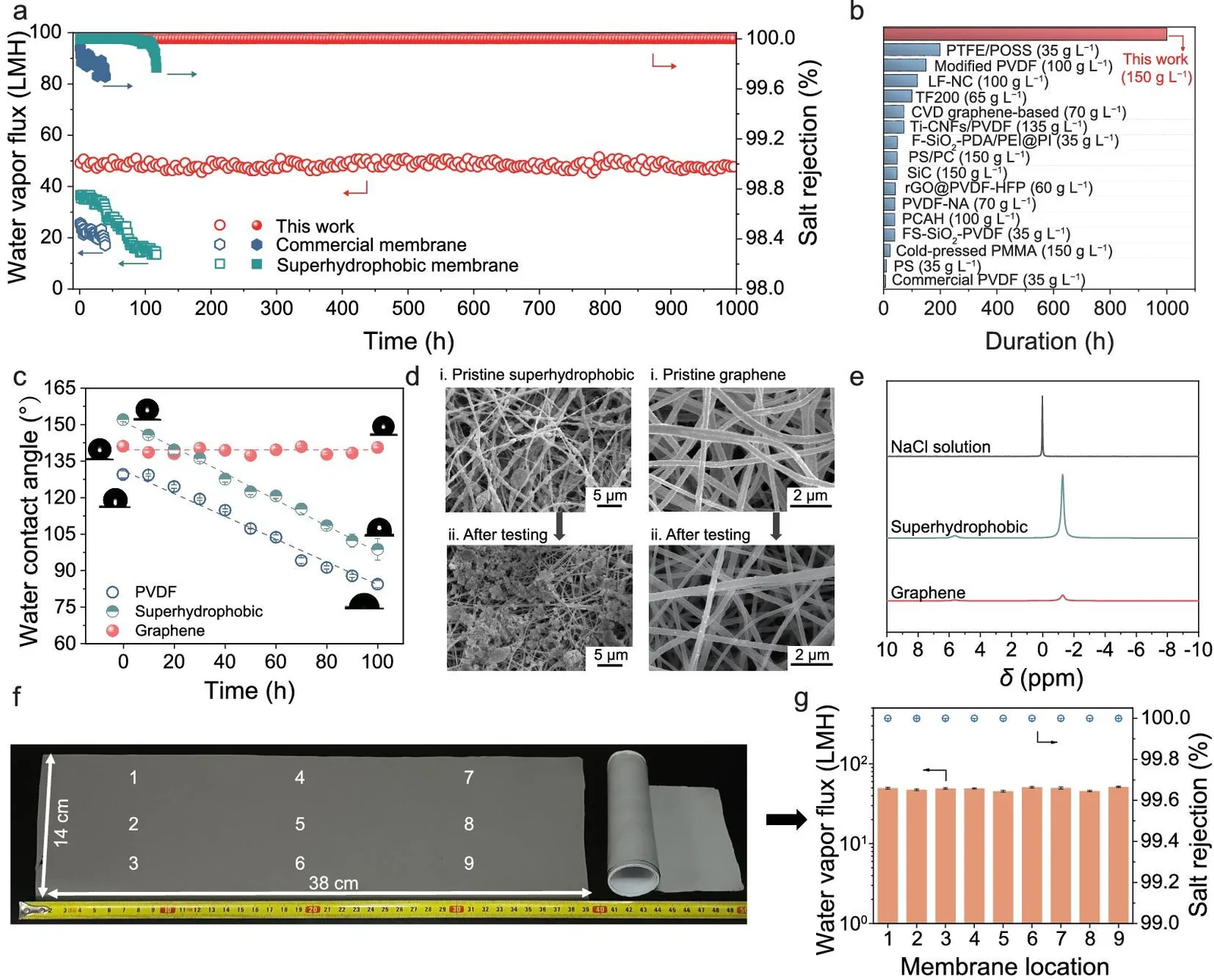
Membrane separation stability and scale-up fabrication. (a) Water vapor flux and salt rejection of a commercial PVDF membrane (Millipore Durapore, HVHP04700), a superhydrophobic membrane and the graphene nanofiber membrane during the long-term testing. The feed temperature was 60°C, with NaCl concentration of 150 g L^−^^1^. (b) Comparison of operational duration for water desalination relative to state-of-the-art membranes. (c) Water contact angles of graphene, commercial PVDF (Millipore Durapore, HVHP04700) and superhydrophobic membranes during the high-salinity water desalination process. (d) SEM surface images of superhydrophobic and graphene nanofiber membranes before and after water desalination testing. (e) ^23^Na nuclear magnetic resonance (NMR) of superhydrophobic and graphene nanofiber membranes after immersion in high-salinity water (150 g L^−^^1^). 0.1 M NaCl in water was used as a control. (f) Photograph of the large-area graphene nanofiber membrane. (g) Desalination performance of nine different sections from the scaled-up graphene nanofiber membrane shown in panel (f) tested under feed and permeate temperatures of 60°C and 20°C, respectively, and with a feed NaCl concentration of 150 g L^−^^1^.

To assess the durability of the graphene membrane, we performed the challenging hypersaline desalination tests under extreme conditions. The feed solution contained 150 g L^−^^1^ NaCl, well beyond typical operational limits. We initially tested the long-term stability of a commercial PVDF membrane (Millipore Durapore, HVHP04700) for comparison. We observed 2–4 times lower flux and lost rejection within 5 h, accompanied by visible surface salt crystallization (Fig. 4a and Fig. S52). To further elucidate the role of surface properties, we engineered a superhydrophobic PVDF membrane (water contact angle: 152°) with a pore structure similar to that of the graphene membrane (Fig. 4c). Although it remained stable under moderate salinity (35 g L^−^^1^; Fig. S53), it underwent rapid performance degradation within 100 h at 150 g L^−^^1^ (Fig. 4a), owing to irreversible salt crystallization (Fig. 4d). These adsorbed salts reduce membrane hydrophobicity and obstruct transport pathways (Fig. 4e), resulting in decreased salt rejection and water flux. This phenomenon is directly evidenced by a sharp decline in water contact angles—from 131° to 85° for PVDF and from 152° to 98.9° for the superhydrophobic PVDF membrane during prolonged desalination operations (Fig. 4c).

In contrast, our graphene membrane with a continuous skin layer maintained a stable water contact angle (∼140°), with no obvious salt crystallization on the membrane surface (Fig. 4c–e), enabling a high-water flux and near-perfect salt rejection (>99.99%) for over 1000 h—performance levels one to two orders of magnitude greater than those of state-of-the-art membrane distillation membranes (Fig. 4a and b). This result overcomes the conventional trade-off between flux and operational lifetime that constrains current high-salinity desalination membrane.

We further explored the scalability of the graphene membrane (Fig. 4f). A membrane sheet measuring 38 cm × 14 cm exhibited reproducible desalination performance and could be conveniently rolled into a module format, highlighting the process compatibility with large-scale manufacturing (Fig. 4g). The ferroelectric-polarization-assisted fabrication method we propose overcomes the inherent limitations of CVD-derived graphene membranes, including challenging transfer procedures and limited scalability, offering superior processability and larger membrane areas [38]. Although the thermal reduction process introduces non-ideal structural features, such as defective *sp*^3^ domains and trace residual chemical groups [39], our results confirm that these imperfections are acceptable [18] and do not significantly compromise ultra-fast molecular transport. This membrane formation strategy demonstrates broad applicability across two-dimensional materials. We successfully fabricated ultra-thin, highly porous MXene and MoS_2_ membranes (Figs S54 and S55) with structural characteristics analogous to the graphene membrane.

### Molecular transport mechanism

**Figure 5.**
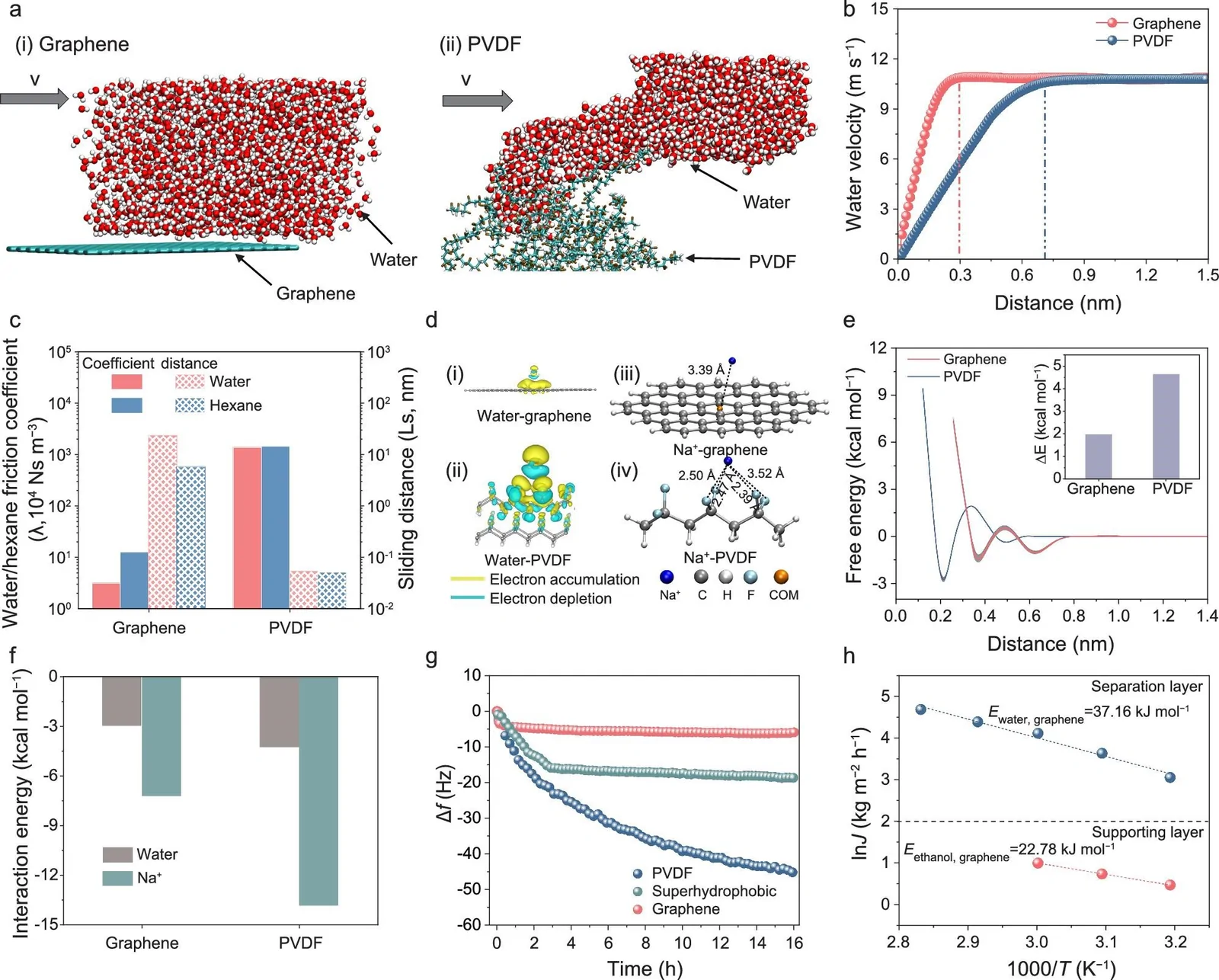
Membrane transport mechanism. Molecular simulations and DFT calculations: (a) representative snapshots showing water sliding on (i) graphene and (ii) PVDF under identical conditions. (b) Corresponding velocity distribution of water molecules on graphene or PVDF surface. (c) Friction coefficient and sliding distance for water and *n*-hexane on graphene and PVDF. (d) Electron density difference isosurface (isovalue = 0.0002 e Bohr^−^^3^) for (i) graphene and (ii) PVDF. Optimized geometry of (iii) the Na^+^–graphene complex and (iv) the Na^+^–*β*-PVDF complex. The center of mass (COM) is indicated for the six-carbon ring in graphene. The distances between the ions and the COMs, along with several of the shortest atomic distances between the ions and PVDF, are denoted by the dotted lines. The distance unit is given in Å. (e) PMF profiles for water escaping graphene and PVDF surfaces. Inset: corresponding free energy barriers. (f) Interaction energies between the water or Na^+^ ions and graphene or *β*-PVDF. (g) Frequency shift (Δ*f*) of water measured for PVDF, superhydrophobic and graphene membranes using QCM-D. (h) Activation energies of water and ethanol for graphene membranes acting as separation layers or as supports during hypersaline desalination and pervaporation recovery of ethanol from aqueous solution.

The ultra-fast transport behavior observed in two-dimensional nanochannels is attributed to the low-friction transport pathways and slip-flow effects induced by nanoscale confinement [40–42]. We hypothesized that the ultra-fast permeation of water and organic solvents through the graphene membrane originates from molecular sliding facilitated by its atomically smooth surface. To elucidate this mechanism, we first simulated the flowing behavior of water across graphene and PVDF membrane surfaces. In stark contrast to the hindered flow and strong adsorption observed on PVDF, water molecules exhibited a nearly frictionless glide across the graphene surface (Fig. 5a and Fig. S56). The velocity distribution revealed a fundamental disparity in interfacial momentum transfer between water and the two surfaces (Fig. 5b). Water flow attained a steady-state velocity within a remarkably short distance of ∼0.3 nm on graphene, less than half that observed on PVDF (∼0.7 nm). These distances, comparable to the theoretical thickness of a single graphene layer or a PVDF polymer strand, signify the extent of the interface-dominated region. This indicates that water achieves rapid momentum coupling within an atomic-scale zone on graphene, whereas the broader, more dissipative interface of PVDF retards velocity development. Such a contrast in interfacial coupling length substantiates the near-frictionless sliding of molecules along the atomically smooth graphene surface. Quantitatively, the water friction coefficient on graphene was determined to be 3.15 × 10^4^ Ns m^−^^3^, three orders of magnitude lower than that on PVDF (1.37 × 10^7^ Ns m^−^^3^) (Fig. 5c). A similarly dramatic reduction was observed for hexane (1.27 × 10^5^ Ns m^−^^3^ on graphene versus 1.42 × 10^7^ Ns m^−^^3^ on PVDF). Correspondingly, the calculated slip lengths for water and hexane on graphene [43] were two to three orders of magnitude larger than those on PVDF, consistent with reduced interfacial friction.

We studied the slip length for water on the graphene and PVDF using the classical hydrodynamics equation, modified by the slip term [43,44] (Fig. S57):


(1) \begin{eqnarray*}
\frac{{\Delta P}}{R}{\mathrm{\ = \ }}{\rho }_{\mathrm{0}}\frac{{\left( {\frac{{{\mathrm{2}}\sigma {\mathrm{cos}}\emptyset }}{h}{\mathrm{\ + \ }}\frac{A}{{6\pi {h}^{\mathrm{3}}}}} \right){h}^{\mathrm{4}}}}{{{\mathrm{12}}\eta L}}\left( {{\mathrm{1\ + \ }}\frac{{6\delta }}{h}} \right),
\end{eqnarray*}

where Δ*P* is the pressure difference driving the flow and *R* represents the flow resistance. *ρ*_₀_ and *η* denote the density and viscosity of water, respectively. *σ* is the surface tension of water and *θ* is the contact angle. *h* and *L* are the height and length of the channels in the PVDF or graphene membranes, respectively. *A* is the Hamaker constant (e.g. *A* ≈ 115 zJ for the graphene/water system [45]) and *δ* is the water slip length. The obtained slip length ratio of water–graphene to water–PVDF was 1.96, which is close to the measured water flux ratio of the graphene membrane to the PVDF membrane (1.73) during water desalination process (Table S1). The observed deviation in slip length ratio from simulation is attributable to non-ideal structural features. The free energy barrier for water molecules to desorb from the PVDF surface into the bulk phase was ∼2.4 times higher than that from the graphene surface (Fig. 5e), providing a thermodynamic rationale for the nearly frictionless molecular sliding on graphene.

We calculated the molecular interaction of water and ions with graphene and PVDF. The molecular interaction energy (Δ*E*_int_) of water with graphene and PVDF was determined to be −2.9 and −4.3 kcal mol^−^^1^, respectively (Fig. 5f and Fig. S58), indicating that the water interacts more weakly with graphene than with PVDF. The molecular polarity index—an indicator of the local molecule or surface polarity—was 5.3 kcal mol^−^^1^ Å^−^^2^ for graphene and 27.0 kcal mol^−^^1^ Å^−^^2^ for PVDF, showing that the latter is over 5 times more polar. The electron density difference maps for water adsorption on graphene and on PVDF (Fig. 5d-i and ii) further reveal that charge transfer, which is positively correlated with polarization, is considerably greater on PVDF than on graphene. The weaker molecular interaction on graphene correspondingly lowers the activation energy for water transport (Table S6), thereby facilitating the observed ultra-high water flux (Fig. 4a). A similar trend was observed for ion interactions: the calculated *ΔE*_int_ for Na^+^ and Cl^−^ in water with PVDF is 1.8–1.9 times higher than that with graphene (Fig. 5d-iii and iv and Fig. S58), confirming the generally weaker interfacial binding on graphene surfaces.

We further measured the interfacial adhesion properties between the membrane and feed species. Using the QCM-D system, we evaluated the dynamic water adsorption behavior on graphene, PVDF and superhydrophobic membranes (Fig. S22). The frequency shift (Δ*f*) for graphene was markedly less negative than those for PVDF and the superhydrophobic surface (Fig. 5g), indicating a substantially weaker water–surface affinity. Consistently, the graphene membrane exhibited >8-fold lower mass uptake in high-salinity water compared with PVDF, and nearly 3-fold lower than its superhydrophobic analogue (Fig. S59). In parallel, markedly stronger sodium signals on the PVDF membrane relative to the faint response observed on graphene surfaces were revealed by ^23^Na NMR spectroscopy (Fig. 4e). These results collectively confirm the suppressed salt adhesion on graphene membrane.

These findings reveal a fundamental limitation of conventional membrane surfaces such as PVDFs, which exhibit nanoscale chemical heterogeneity and physical roughness, providing affinity sites for molecular interactions and restrict molecular motion [46]. While surface fluorination grafts low-surface-energy –CF_3_ groups to enhance hydrophobicity by minimizing interfacial energy and improving chemical inertness [33], the resulting surfaces of superhydrophobic PVDF membrane still retain atomic-scale chemical heterogeneity, sustaining a higher water adsorption capacity than graphene. Under hypersaline conditions, these heterogeneous surface regions act as nucleation sites, promoting heterogeneous crystallization and subsequent growth of salt crystals. This process not only physically compromises the structural integrity of the hydrophobic layer but also establishes capillary transport pathways for liquid water, ultimately triggering membrane wetting and rejection failure. Indeed, as shown in Fig. 4a, durability decayed within 100 h.

In contrast, the chemically homogeneous and atomically smooth graphene surface forms a uniform interface with minimal energy corrugation [47]. Its *sp*^2^-carbon lattice lacks chemical heterogeneities that would restrict molecular motion, enabling super-sliding for molecules of diverse chemical natures, including polar water and non-polar hexane. Moreover, the graphene surface reduces the tendency for salt ion accumulation, thereby diminishing the thermodynamic driving force for nucleation. Its remarkable chemical inertness [48] confers exceptional resistance to prolonged exposure to hypersaline environments, thus sustaining for over 1000 h. Furthermore, unlike conventional support layers, the molecular sliding effect on the graphene surface allows permeate molecules to migrate with minimal energy dissipation after leaving the separation layer, thereby lowering the transport resistance of the support. This effect is directly reflected in the substantially reduced activation energy for ethanol permeation—22.78 kJ mol^−^^1^ for the graphene supported PDMS membrane versus 35.6–53.4 kJ mol^−^^1^ for reported PDMS membranes—and for water permeation—37.16 kJ mol^−^^1^ for the graphene membrane versus 46.1–59.4 kJ mol^−^^1^ for reported hypersaline desalination membranes—corroborating the molecular sliding transport mechanism (Fig. 5h and Tables S6 and S7).

## CONCLUSIONS

Our results demonstrate that the graphene membrane—with its chemically homogeneous and atomically smooth surface—exhibits markedly reduced interfacial interactions with feed components compared with conventional membranes. This property gives rise to molecular sliding, enabling ultra-fast and stable separation processes. These findings shift the paradigm of membrane separation from the conventional pore-size-dominated perspective toward a framework governed by atomic-scale surface smoothness. Beyond the GO/PVDF system, the ferroelectric-polarization-assisted strategy is potentially generalizable to other ferroelectric polymer–2D material systems. This work shows a versatile route for constructing atomically smooth surfaces, establishing a materials platform not only for diverse membrane separation processes but also for fundamental studies of nanoscale molecular transport and related applications.

## Supplementary Material

nwag419_Supplemental_File
