# Supplementary material for: A ferroelectric-induced few-nanometer-thick graphene membrane enables molecular sliding for ultra-fast separation
Source: Natl Sci Rev. 2026 Jul 9;13(17):nwag419. doi: 10.1093/nsr/nwag419 (PMC13599783; doi:10.1093/nsr/nwag419)
Supplement: Supplementary file 1 [file nwag419_supplemental_file.pdf]

Supplementary Materials

A ferroelectric-induced few-nm-thick graphene membrane enables molecular sliding for ultra-fast separation

Wenqi Ji[1,†], Yanan Guo[1,†], Guozhen Liu[1,*], Binyu Mo[1], Huan Yu[1], Kaihua Gao[1], Jiasheng Wu[1], Yangyang Mao[1], Huimin Chen[1], Gongping Liu[1,2,*], Young Moo Lee[1,3,*], Wanqin Jin[1,*], Nanping Xu[1,2]

[1]State Key Laboratory of Materials-Oriented Chemical Engineering, College of Chemical Engineering, Nanjing Tech University, Nanjing 211800, China;

[2]Suzhou Laboratory, Suzhou 215100, China and [3]Department of Energy Engineering, College of Engineering, Hanyang University, Seoul 04763, Republic of Korea

∗**Corresponding authors.** E-mails: guozhenliu@njtech.edu.cn; gpliu@njtech.edu.cn; ymlee@hanyang.ac.kr; wqjin@njtech.edu.cn

[†]Equally contributed to this work.

This PDF file includes:

Materials and Methods

Supplementary Discussion

Supplementary Figures 1 to 59

Supplementary Tables 1 to 7

References

Materials and Methods

Materials

Poly(vinylidene fluoride) (PVDF) powders, M_w = 500,000 g mol^{-1}, were obtained from Kureha Corporation, Japan. Poly(methyl methacrylate) (PMMA) powders, M_w = 996,000 g mol^{-1}, boric acid (H_3BO_3), cesium chloride (CsCl), strontium chloride ($SrCl_2$), cobaltous chloride ($CoCl_2$), dibutyltin dilaurate (DBTDL, ≥95%) and Span 80 were purchased from Sigma-Aldrich. Nano SiO_2 particles (30 nm), 1H,1H,2H,2H-perfluorodecyltriethoxysilane (PFDTES, (≥96%) was obtained from Aladdin Biochemical Technology Co., Ltd, China. N, N-Dimethylformamide (DMF, >99.5%), N-hexane (≥97%), isopropyl alcohol (IPA, >99.7%), sodium nitrate ($NaNO_3$), sodium chloride (NaCl), tetraethyl orthosilicate (TEOS), n-heptane, and petroleum ether were purchased from Sinopharm Chemical Reagent Co., Ltd. Lithium chloride (LiCl) was provided by Xilong Scientific Co., Ltd, China. Dihydroxypolydimethylsiloxane (PDMS, M_w = 60,000) was supplied by Shanghai Resin Factory Co., Ltd. Hexadecane (98%) was purchased from Macklin Biochemical Technology Co., Ltd, China. Acetone (>99.5%) was purchased from Jiangsu Yonghua Fine Chemical Co., Ltd. Graphene oxide (GO) nanosheets with differenrt lateral size were supplied by Nanjing Jicang Nanomaterials Technology Co., Ltd, China. Ti_3C_2Tx MXene and MoS_2 nanosheets were purchased from XFNANO Materials Technology Co., Ltd. Commercial PVDF membranes were supplied by Millipore. All chemicals were used without further purification.

Membrane preparation

GO was dispersed into 30 mL DMF/acetone solution (4:1 v/v) and ultrasonicated for 2 h to obtain uniform dispersions. PVDF (3 g) and LiCl (0.0025g) powders were added, and the mixture was stirred until homogeneous. GO concentrations were were varied at 1-5 mg mL^{-1}. Air bubbles

were removed by vacuum suction, and the solution was electrospun through a 21-gauge needle to fabricate GO@PVDF membrane. During this process, the solution was injected from the needle to the collector at a flow rate of 2 mL h^{-1} under a voltage of 10~20 kV and relative humidity of 60%. The distance between the needle and the collector was 20 cm. The PVDF nanofiber was fabricated similarly without GO. GO@PVDF membranes were thermally reduced at 60~180°C for 12 h to obtain graphene membranes. GO nanosheets with lateral sizes of 5–8 μm and 20–30 μm were fabricated into membranes using the same procedure, followed by thermal reduction at 140°C for 12 h to obtain the graphene membranes. MXene and MoS_2 membrane were prepared via analogous procedures to demonstrate method universality.

For comparison, GO/PVDF mixed-matrix membranes were prepared via nonsolvent-induced phase separation (NIPS). The GO-PVDF solution (3 mg mL^{-1} GO) was cast on a glass plate using doctor blade (400 μm gap) and immersed in ethanol for 12 h, followed by drying at room temperature and thermal treatment at 140°C for 12 h. Superhydrophobic membranes were prepared by electrospinning SiO_2-PVDF solutions, followed by immersion in 5 wt% PFDTES/n-hexane for 12 h and drying at 100°C for another 12 h.

PDMS/graphene composite membranes were prepared by spin-coating a PDMS/n-heptane solution (3 wt% PDMS) with DBTDL catalyst and TEOS crosslinker (mass ratio 1:10:100) onto the graphene substrate using a two-step protocol (300 rpm for 9 s, then 3000 rpm for 30 s). Coated membranes were exposed to air for 12 h and heated at 60°C for an additional 12 h.

Characterizations

Morphologies were observed by field emission scanning electron microscope (FE-SEM, Hitachi, S-4800 and Phenom Pharos G2, Netherland) and transmission electron microscopy (TEM, Talos F200X G2 and Themis Z, USA). Surface topography was evaluated via atomic force

microscope (AFM, Bruker Dimension Icon, Germany), and elemental composition by energy dispersive spectroscopy (EDS). X-ray photoelectron spectroscopy (XPS, Thermo ESCALAB 250, USA) determined chemical composition and depth profiles. Ferroelectric properties were evaluated by a ferroelectric tester (TF2000e, Germany). Water contact angles (WCA) were obtained with a contact angle meter (Biolin Scientific, Theta Lite, Finland) using deionized (DI) water droplets on the membrane surface to calculated WCA using Young-Laplace equation, with three replicates per sample. Mechanical properties were evaluated using a dynamic mechanical analyser (DMA, TA Instruments, DMA850). ^{23}Na NMR spectra were acquired on a 600 MHz WB solid-state NMR spectrometer (Bruker AVANCE NEO 600 WB). The polarization-electric field (P-E) hysteresis loop of samples was conducted using a ferroelectric analyzer (ACIX TF2000E, Germany). Dielectric properties were measured using an broadband dielectric impedance spectrometer (NOVOCONTROL Concept80, Germany) in a frequency range from 1 Hz to 1 MHz at room temperature.

Crystal structure and chain packing were analyzed by X-ray diffraction (XRD, Rigaku, MiniFlex600), small-angle X-rays scattering (SAXS) and wide-angle X-ray Scattering (WAXS, Xeuss 3.0, France), and Fourier transform infrared spectroscopy (FTIR, PerkinElmer, L1600300 Spectrum Two Lita). Specifically, the WAXS experiment was performed with a sample-to-detector-distance of 5 cm and a resolvable scattering vector modulus (q) range from 2 to 27 nm^{-1}. SAXs experiment was performed with a sample-to-detector-distance of 107 cm and a resolvable scattering vector modulus (q) range from 0.07 to 2.20 nm^{-1}.

Average nanosized chains distance (d) based on the peak position q_0 from SAXs patterns was obtained according to the following equation:

$$d=\frac{2\pi}{q_0} \tag{1}$$

where the peak position q_0 was obtained from the Lorentz-corrected SAXS curve.

Membrane porosity was determined by a gravimetric method. Specifically, the dried membrane with mass of m_p was immersed into isopropanol (IPA) overnight to make sure that all the membrane pores were impregnated by IPA molecules. After being wiped off by airlaid paper, the humid membrane was then weighted as m_l. Thus, the membrane porosity (ε) can be calculated according to the following equation:

$$\varepsilon = \frac{m_l/\rho_l}{m_l/\rho_l + m_p/\rho_p} \tag{2}$$

where ρ_l and ρ_p represent the density of IPA and polymer, respectively.

Salt adsorption experiment was conducted by immersing the membrane (cut into 2 × 2 cm piece) into 150 g L^{-1} NaCl solution at 60°C, wherein the membrane was fixed using two O-rings. During the testing process, we took out the membrane every hour, wiped off the solution remained on the surface using dust-free paper, and then evaluated the mass change using a balance.

Adsorption test of H_2O was analyzed using a quartz crystal microbalance with dissipation (QCM-D) system. The samples were prepared by depositing PVDF, GO@PVDF and superhydrophobic PVDF membranes on the QCM sensor using the electrospinning method. The graphene sample was obtained by annealing the GO membrane at 140°C for 12 h. All the samples were dried at 40°C under vacuum for 24 h before testing. After placing the sensor on the module, the surface was purged with nitrogen at a flow rate of 50 mL min^{-1} for 12 h to exclude the influence of inert gas. The water vapor was introduced by blowing nitrogen (50 mL min^{-1}) into water. The mass changes of the sample on the quartz surface and the frequency (f) of the oscillating crystal measured follow the Sauerbrey relationship[1] (equation (3)), while the dissipation factor (D) is

related to the viscoelastic properties of the adsorbed layers. Therefore, QCM-D can be used to quantify changes in water adsorption by fibers.

$$\Delta m = \frac{-C \times \Delta f}{n} \tag{3}$$

where Δm is the mass of water adsorption, Δf is the change of frequency during the test and n is the overtone. C is a constant value (17.7 ng $Hz^{-1}cm^{-2}$). The sensor with 5 MHz fundamental frequency was used, and Δf can be displayed at both fundamental (n=1) and overtone frequencies (n=3, 5, 7). The data presented here were obtained from the third overtone (n=3).

The energy from the oscillating crystal dissipates when the driving voltage is turned off, which is defined as the energy loss at one period of oscillation. The dissipation factor (D) was used to describe the energy dissipation[2]:

$$D = \frac{E_{lost}}{2\pi E_{stored}} \tag{4}$$

where E_{lost} is the energy dissipated during one oscillation cycle and E_{stored} is the energy stored in the oscillating system**.** In addition, D is inversely proportional to the decay time (τ) as follows:

$$D = \frac{1}{\pi f \tau} = \frac{2}{\omega \tau} \tag{5}$$

where f is the resonant frequency.

Membrane performance evaluation

Desalination performance was evaluated by a hydrophobic interfacial evaporation desalination process (Supplementary Fig. 39). The membrane with an effective area of 4.1 cm^2 was fixed in a membrane cell using two silicon gaskets. The temperatures of feed side (40~80°C) and permeation side (20°C) were controlled by an oil bath (HWCL-3, Greatwall, China) and a heating/refrigerated circulator (Hengmin, China), respectively. The sodium solution (NaCl, 35 g L^{-1} or 150 g L^{-1} or 280 g L^{-1}) or boron-sodium mixed solution (NaCl, 150 g L^{-1}; boric acid, 100

mg L^{-1}) or cesium-sodium mixed solution (NaCl, 150 g L^{-1}; Cs, 100 mg L^{-1}) or simulated radioactive wastewater solution ($NaNO_3$, 50 g L^{-1}; Co (II), 100 mg L^{-1}; Sr (II), 100 mg L^{-1}; and Cs (I), 100 mg L^{-1}) were utilized as the feed solution. The permeate solution (cold water) and the feed solution were cycled through the membrane cell at the rates of 1.6 L min^{-1} and 0.18 L min^{-1}, respectively, using two gear pumps (Leadfluid, CT3000S). The water vapor flux (J, L m^{-2} h^{-1}), LMH) across the membrane was measured by monitoring the weight change in permeate solution using a digital balance (BSA2202S, Sartorius, Germany). The salt rejection (R, %) was evaluated based on the ions concentration in the permeate side measured by a calibrated conductivity meter (CS3702D, Clean, China). The weight change and ions concentration in the permeate side were recorded using a real-time online recording system.

Water vapor flux and salt rejection were calculated according to the following equations:

$$J = \frac{\Delta m}{A \times \Delta t} \tag{6}$$

$$R = \left[1 - \frac{\Delta(V_p \times C_p)}{J \times A \times \Delta t \times C_f}\right] \times 100\% \tag{7}$$

where Δm (kg) represents the weight change in permeate solution, A (m^2) is the effective membrane area, Δt (h) is the time interval, V_p (L) stands for the volume of the permeate solution, and C_f(mg L^{-1}) and C_p (mg L^{-1}) are the salt concentration of feed and permeate solutions, respectively.

Permeance was calculated by normalizing the measured water flux with the transmembrane vapor pressure difference (ΔP), as follows[3]:

$$\Delta P = P_f - P_p \tag{8}$$

$$Permeance = \frac{flux}{driving\ force} = \frac{J}{\Delta P} \tag{9}$$

where ΔP (Pa) is the saturated vapor pressure difference across the membrane. P_f (Pa), P_p (Pa) are the saturated vapor pressure on the feed and permeate sides of the membrane, respectively. These values were determined through process simulation using Aspen Plus, taking into account the temperature and salt concentration of each stream.

Oil-water separation performance was evaluated by a gravity-driven filtration system (10 cm liquid column) (Supplementary Fig. 46). The effective membrane area is 1.77 cm^2. The water-in-oil elmusion was prepared by adding 5 mg of span 80 into 99 mL petroleum ether, n-Hexane, or n-Hexadecane, and then 1 ml water was added. The mixture was stirred for several hours. The permeance (P) and rejection (R) are usually used to evaluate membrane performance. They can be calculated by the following equations:

$$P=\frac{V}{A\times t\times \Delta p} \tag{10}$$

$$R=(1-\frac{C_p}{C_f})\times 100\% \tag{11}$$

where V (L) is the volume of permeate, A (m^2) represents the effective membrane area, t (h) is the time interval, Δp (bar) is the applied pressure. C_p and C_f are water concentrations in the permeate solution and the feed solution, respectively, which were measured using a Karl Fischer moisture analyzer (Metrohm, 870 KF Titrino plus, Switzerland).

Organic solvents separation performance of PDMS@graphene membrane was evaluated by a home-made pervaporation apparatus (Supplementary Fig. 49). Ethanol-water solution with an ethanol concentration of 5 wt% was used as the feed solution. It was circulated between the feed tank and membrane module using a peristaltic pump at a flow rate of 100 mL min^{-1}. The effective membrane area is 2.3 cm^2. The pressure on the permeate side was maintained below 300 Pa using a vacuum pump. The permeated organic vapor was collected by a cold trap placed in liquid-

nitrogen. The ethanol concentrations of feed and permeate solutions were detected by a gas chromatography (Shimadzu, GC-2014, Japan).

Total flux (J, kg m^{-2} h^{-1}) and separation factor (β) were calculated according to the following equations:

$$J=\frac{M}{A\times t} \tag{12}$$

$$\beta=\frac{y_p/(1-y_p)}{x_f/(1-x_f)} \tag{13}$$

where M is the total weight of permeate (kg), A is the effective membrane area (m^2), t is the testing time interval (h). y_p and x_f are the ethanol mass concentrations in the feed and permeate sides, respectively.

Molecular dynamics simulations.

MD simulations were performed and analyzed using GROMACS 2022.5[4], with the AMBER force field applied for the GO and graphene nanosheets, the GAFF force field applied for the PVDF chains, and the DMF molecules. The SPC/E (extended simple point charge) model was used to describe water molecules. The visual molecular dynamics program was used for the visualization of the trajectories[5]. The charges of functional groups of GO were adopted from the ref[6], resulting in 0 total charge of GO. The topology for the GO was generated using the x2top tool implemented within GROMACS. The topologies for PVDF chains and DMF molecules were generated using the Sobtop code[7, 8]. The restrained electrostatic potential (RESP) atomic charges of the DMF molecule was calculated through the Multiwfn program[9, 10] based on the wavefunctions obtained at the B3LYP-D3(BJ)/def2-TZVP level of theory using the ORCA program[11] .

Free Energy Calculations.

To calculate the free energies of GO across PVDF chains, two PVDF chains with proper interval and one GO nanosheet were solvated in the DMF solvent. The oxidized structure of GO was built based on the Lerf-Klinowski model[12], that is the hydroxyl and epoxy groups were randomly distributed on the carbon basal plane. The size of the GO is about 2 nm × 2 nm (Supplementary Fig. 35). Initially, the GO was put far away from the polymer chains. The system was placed in a rectangular box with dimensions sufficient to satisfy the minimum image convention and provide space for the steered molecular dynamics (SMD) simulations.

In order to generate starting structures for the SMD simulations, the built model was first energy minimized using the steepest descent method. Then the system was equilibrated in the constant pressure (NPT) ensemble for 50 ps with position restraints applied to PVDF chains and GO to preserve the fixed interval between two polymer chains. The temperature and the pressure were maintained at 300 K and 1.0 bar, respectively, using the Berendsen weak coupling method[13]. Periodic boundary condition was applied in all directions. Structure of the last frame of the trajectory was used as starting configuration for SMD simulations. In this study, the reaction coordinate corresponds to the y-axis. The GO was pulled across the PVDF chains along the y-axis over 500 ps, using a spring constant of 1000 kJ mol^{-1} nm^{-2}) and a pull rate of 0.01 nm ps^{-1}. From the SMD trajectory, snapshots were taken to generate the starting configurations for the umbrella sampling (US) windows. The window spacing was 0.2 nm, resulting in 25 windows. In each window, 10 ns of simulation was performed, resulting in a total simulation time of 250 ns for umbrella sampling. The weighted histogram analysis method, as implemented within GROMACS, was used to calculate the potential of mean force (PMF) free energy profiles[14]. The histograms of US windows overlap significantly along the reaction coordinate, as can be seen from Supplementary Fig. 32. This indicates that sufficient sampling has been obtained.

The periodic graphene and PVDF surfaces were built for the calculation of water molecule and hexane molecule desorption free energies. The adaptive-biasing force algorithm was employed to achieve the PMF. The reaction coordinate was set in the *z*-direction ranging from 0-14 Å, departing from the surface.

GO distribution in PVDF/GO system.

In order to understand the properties of the PVDF-GO system after the evaporation of the solvent, we built two representative models for periodic molecular dynamics simulations: (1) GO are trapped inside the intertwined PVDF chains (referred to as model 1, Supplementary Fig. 36a); (2) GO are on the surface of the polymer chains (referred to as model 2, Supplementary Fig. 36b). In each model, 30 β-phase polymer chains containing 30 monomers each forming the polymer. The size of the GO is ca. 2 nm × 2 nm. 5 ns simulations at the NVT ensemble was performed to keep the volumes of the systems constant. The energies of the equilibrated systems were calculated.

Friction Coefficient Calculation.

The model of water confined between graphene sheets was built to calculate water-solid friction coefficient. The size of the graphene sheet is 3.69 Å × 3.69 Å. The distance between the two opposite graphene sheets was 3 nm. There were 1222 water molecules within the channel. For comparison, the PVDF model was built. There were 1167 water molecules for the PVDF model.

Equilibrium MD simulations of 10 ns at NVT ensemble were performed on the two models. Periodic boundary conditions (PBCs) were applied in x, y and z directions. The temperature was maintained at 300 K. The graphene sheets and the carbon chains of PVDF sheets were fixed in the simulations. The water-solid friction coefficient λ was calculated from the autocorrelation function of interfacial forces according to the Green-Kubo formulation,

$$\lambda = \frac{1}{Ak_BT}\int_0^t \langle F_w(0)F_w(t)\rangle dt \tag{14}$$

Herein, A, k_B, T, and F_w represent the surface area, Boltzmann constant, absolute temperature, and instantaneous shear force exerted on the solid, respectively. The calculated water-graphene friction coefficient λ is 3.15 × 10^4 Ns m^{-3}, which is consistent with previous report[15]. The friction coefficient λ was related to the slip length l_s by using l_s=μ/λ, where μ is the viscosity of water (0.729 mPa s for the SPC/E water model at 300 K [16]. The slip length is widely used to describe the slip flow. The friction coefficients of hexane were calculated using the same method.

Density functional theory (DFT) simulations.

Interaction energy between water molecule and graphene/PVDF.

In order to study the interaction properties of water molecule with graphene, the periodic pure graphene model was built. For comparison, the PVDF surface model was also constructed. Since after electrospinning the *β*-phase crystalline PVDF is mainly formed, the *β* (020) crystal surface was cleaved to study the interaction of water molecule with PVDF membrane [17]. The vacuum of 20 Å along the z-axis was added to avoid imaging interactions for all periodic models.

The interaction energy was calculated using the DFT method through the Vienna Ab-initio Simulation Package (VASP)[18]. The projector augmented wavefunction (PAW) method[19] was employed to describe the electron-core interactions. The Perdew-Burke-Ernzerhof (PBE) functional in a plane wave pseudopotential implementation within the generalized gradient approximation (GGA) was applied to deal with the electron exchange-correlation[20]. This method has been widely used for main group elements [21, 22]. Grimme's semi-empirical correction for the dispersion potential (DFT-D3) was applied to treat the long-range dispersion effect[23]. Plane wave cutoff energy of 450 eV was used for all the models. The Monkhorst-Pack technique was used to automatically generate the gamma centered 3×3×1 k-points grid in the Brillouin zone[24]. The electronic self-consistent iteration convergence criterion was 1.0×10^{-6} eV.

The convergence criteria for the force was 0.02 eV/Å for each free atom. The figures were generated using the visual molecular dynamics (VMD) program[5] and the visualization for electronic and structural analysis (VESTA) program[25].

The interaction energy between water molecule and the surfaces was calculated according to the following equation:

$$\Delta E_{\text{int}} = E_{\text{sur\_ads}} - E_{\text{sur}} - E_{\text{ads}} \tag{15}$$

E_{sur_wat} is the energy of the surface with adsorbent, and E_{sur} and E_{ads} are the energies of the isolated surface and the isolated adsorbent, respectively. The more negative of the ΔE_{int}, the stronger of the interaction. Several initial configurations of the adsorbent on the surface were considered to get the adsorption geometry with the minimum interaction energy. All the interaction energies in this study were calculated using the same way as described here.

In order to get a deep understanding to the surface properties, we performed the quantitative analysis on the Bader defined molecular van der Waals (vdW) surfaces (electron density (ρ) = 0.001 a.u. isosurface) of the graphene surface and the PVDF surface using the Multiwfn program[10]. The wavefunction was obtained using the CP2K package[26]. The electron exchange-correlation was treated through the GGA PBE method. Grimme's semi-empirical correction for the dispersion potential (DFT-D3) was applied to treat the long-range dispersion effect. The short range molecularly optimized double-ζ valence polarized Gaussian basis functions was used to describe the electron-core interactions[27]. A 500 Ry cut-off was used for the plane wave expansion.

Interaction energy between GO and PVDF.

GO with randomly distributed hydroxyl groups and epoxy groups based on the Lerf-Klinowski model was used to study the interaction between GO and PVDF surface. On each base plane of GO, the number of hydroxyl group equals to that of epoxy group. The (020) crystal surface

for both the α-phase and the β-phase crystalline PVDF was cleaved. The GO nanosheet was put over the PVDF surface. The vacuum of 20 Å along the z-axis was added to avoid imaging interactions for all periodic models.

The geometry optimization and single point calculation were all performed using the CP2K package[26]. The electron exchange-correlation was treated through the GGA PBE method. Grimme's semi-empirical correction for the dispersion potential (DFT-D3) was applied to treat the long-range dispersion effect[23]. The short range molecularly optimized double-ζ valence polarized Gaussian basis functions was used to describe the electron-core interactions[27]. A 500 Ry cut-off was used for the plane wave expansion. The wavefunction obtained from geometry optimization of the system was used for the independent gradient model based on Hirshfeld partition (IGMH) analysis to interpret the interaction between GO and PVDF. The IGMH isosurface was color mapped by ρ*sign(λ_2). ρ is the electron density at the weak interaction critical point, sign(λ_2) is sign of the second largest eigenvalue λ_2 of the electron density Hessian matrix. The region with green color represents mainly the weak attractive van der Waals interaction. The cyan-to-blue region represents the relatively strong attractive interactions, including hydrogen bonds and electrostatic attraction. The brown-to-red region represents the prominent repulsive interactions, including steric hinderance and electrostatic repulsion.

Interaction energy between Na^+/Cl^- and graphene/PVDF.

All edge carbon atoms of graphene sheet and PVDF chain were saturated by hydrogen atoms to mitigate edge effects. Firstly, the graphene sheet and PVDF chain were geometrically optimized. Then, the Na^+ or Cl^- ion was placed over or beside the optimized graphene sheet or PVDF chain. The complex was then subjected to geometry optimization. The optimized complex structures, as shown in Fig. 5d (main text) and Supplementary Fig. 56, were used to calculate interaction energies. All the geometry optimizations and single point calculations were conducted under the SMD

(Solvation Model Based on Density) implicit water condition using the ORCA program[28]. The geometry optimizations were performed at the B97-3c level of theory. The single point calculations were performed at the PBE-D3(BJ)/def2-TZVP level of theory.

Supplementary Discussion

Water slip length calculation

We assume a square cross-sectional structure of channels in our graphene membrane. When the channel length (L) is significantly greater than the channel height (h), the fluid velocity u depends solely on the vertical coordinate *z [29, 30]*. By solving the Navier–Stokes equation for planar Poiseuille flow, we obtain:

$$\frac{d^2u(z)}{dz^2} = -\frac{P}{\eta L} \tag{16}$$

where u(z) is the velocity profile along z, P denotes the applied pressure. η is the water viscosity.

From Equation (1), we obtain:

$$\frac{du(z)}{dz} = -\frac{P}{\eta L}z + c_1 \tag{17}$$

$$u(z) = -\frac{P}{2\eta L}z^2 + c_1 z + c_2 \tag{18}$$

where c_1 and c_2 are the integration constants, which can be determined according to appropriate boundary conditions. Here, the system followed the no-slip boundary conditions at the channel walls within graphene membrane, i.e., $u(0)= 0$, *and* $u(h)=0$.

Applying these conditions, the velocity profile for planar Poiseuille flow becomes:

$$u(z)= - \frac{P}{2\eta L}z^2+\frac{P}{2\eta L}zh+u_{slip} \tag{19}$$

Differentiating Equation (4) with respect to z, we obtain the velocity gradient:

$$\frac{du(z)}{dz}= - \frac{P}{\eta L}z+\frac{P}{2\eta L}h+\frac{du_{slip}}{dz} \tag{20}$$

where u_{slip} is the slip velocity at the boundary walls (z=0 or z=h), which is independent of z and is related to the slip length δ as follows:

$$\frac{u_{slip}}{\delta}=\left|\frac{du(z)}{dz}\right|_{z=0} \tag{21}$$

Combining Equations (5) and (6), we get

$$u_{slip}=\frac{Ph\delta}{2\eta L} \tag{22}$$

The ratio of driving force/resistance for fluid flow is expressed as[29, 31]

$$\frac{\Delta P}{R}=\rho_0 h\int_0^h u_z\, d_z \tag{23}$$

where R denotes the flow resistance, which dependents on the channel geometry, fluid viscosity, and slip effects, and ΔP is the pressure difference driving the flow.

Combining Equations (4), (7), and (8), we obtain

$$\frac{\Delta P}{R}=\rho_0 \frac{Ph^4}{12\eta L}\left(1+\frac{6\delta}{h}\right) \tag{24}$$

The total driving pressure P comprises capillary pressure (P_{cap}) and disjoining pressure (P_{disj}) [32]:

$$P=P_{cap}+P_{disj}=\frac{2\sigma\cos \varnothing}{h}+\frac{A}{6\pi h^3} \tag{25}$$

where $P_{cap}=2\sigma cos\phi/h$ arises from the curvature of the water-solid meniscus, and $P_{disj}=A/6\pi h^3$ originates from the interfacial interactions (primarily van der Waals forces) between water and the substrate.

Substituting Equation (10) into (9), we obtain

$$\frac{\Delta P}{R}=\rho_0\frac{\left(\frac{2\sigma\cos\varnothing}{h}+\frac{A}{6\pi h^3}\right)h^4}{12\eta L}\left(1+\frac{6\delta}{h}\right) \tag{26}$$

where:

- σ is the surface tension of water (0.0662 N m^{-1} at 60°C),
- θ is the water contact angle: 141.1° for graphene, and 129.5° for PVDF membranes,
- A is the Hamaker constant (≈115 zJ for the graphene–water system[30] and ≈40 zJ for the PVDF–water system[33]),
- η is the water viscosity (0.467 mPa s at 60°C),
- ρ_o is the water density (0.9832 g cm^{-3} at 60°C).

Based on these parameters, we derived the relationship between the slip length ratio of water–graphene over water–PVDF (δg/δp) and the slip length of water–graphene (δg), as shown in Supplementary Fig. 57. The slip length ratio δg/δp is found to be 1.96 when the slip length of water–graphene is assumed to be 200 nm (ref. [29]).

Theoretical Calculation of GO Layer Thickness

The theoretical thickness of the graphene oxide (GO) layer was calculated based on the volume of a cylindrical shell. It was assumed that a uniform GO layer (shell) completely covers the PVDF nanofiber (core), forming a coaxial double-cylinder structure. The volume of the GO layer (V_G) can be calculated using the following equation:

$$V_G=\pi\,(r+l)^2h-\pi r^2h=\pi\,[\,(r+l)^2-r^2]\times h \tag{27}$$

where:

- r is the radius of the PVDF nanofiber (in nm),
- l is the theoretical thickness of the GO layer (in nm),
- h is the length of the GO nanofiber (in nm).

The mass ratio of GO (m_G) over PVDF (m_P) can be expressed as:

$$\frac{m_G}{m_P}=\frac{\rho_G\times V_G}{\rho_P\times V_P}=\frac{[\,(r+l)^2-r^2]\times h}{r^2h}\times\frac{\rho_G}{\rho_P}=\frac{(r+l)^2-r^2}{r^2}\times\frac{\rho_G}{\rho_P} \tag{28}$$

Solving for the theoretical GO layer thickness l, we obtain:

$$l=\left(\sqrt{\frac{m_G}{m_P}*\frac{\rho_P}{\rho_G}+1}-1\right)\times r \tag{29}$$

where:

- ρ_P=1.78 g cm^{-3} (density of PVDF),
- ρ_G=1.15 g cm^{-3} (density of GO),
- r=150 nm (radius of PVDF nanofiber)

Supplementary Figures and Tables

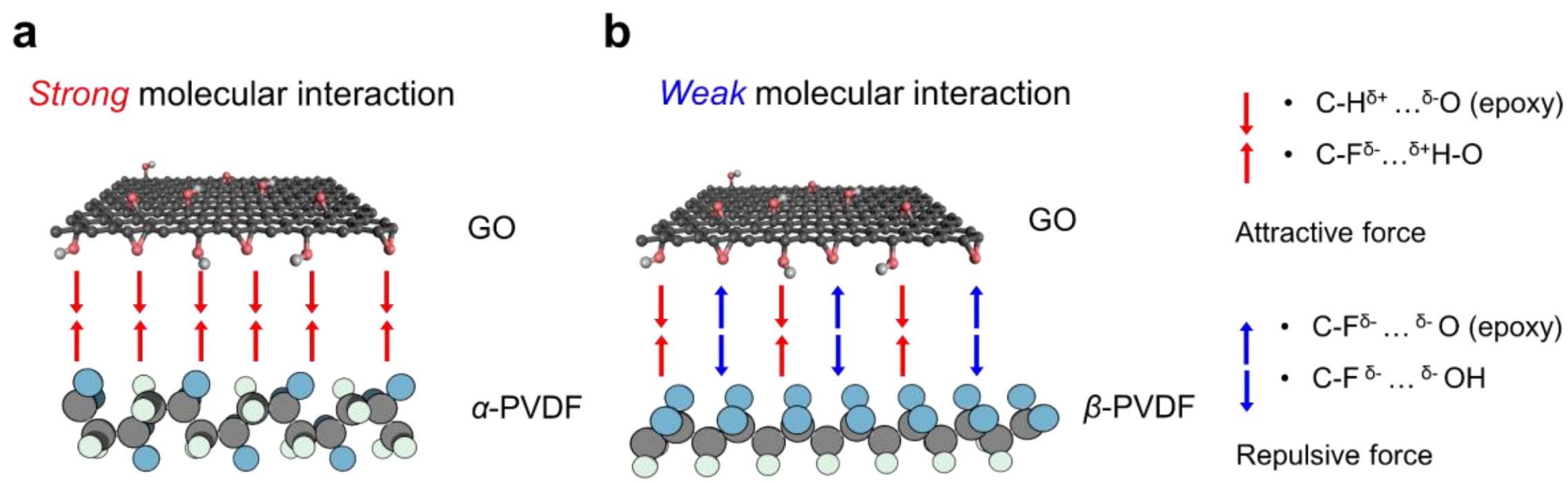

Supplementary Figure 1. Compared with α-PVDF, β-PVDF exhibits weaker molecular interaction with GO.

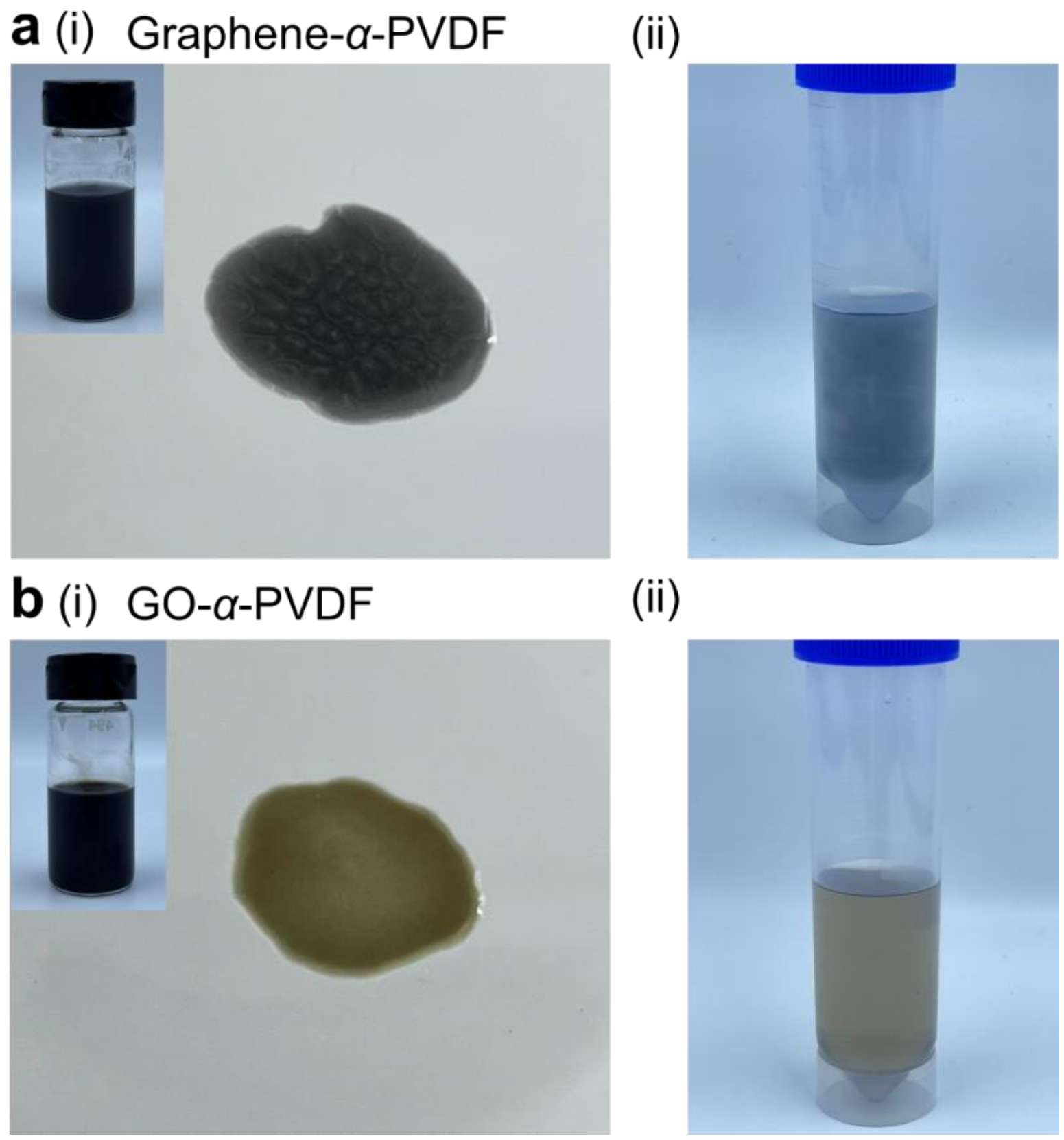

Supplementary Figure 2. Digital photos of graphene-α-PVDF solution (a) and GO-α-PVDF solution (b). In panel (i), the nanosheet concentration is 3 mg mL^{-1}. To clearly visualize the distribution of the nanosheets, the black solution (shown in the inset sin panel (i)) was diluted 300-fold, as shown in panel (ii).

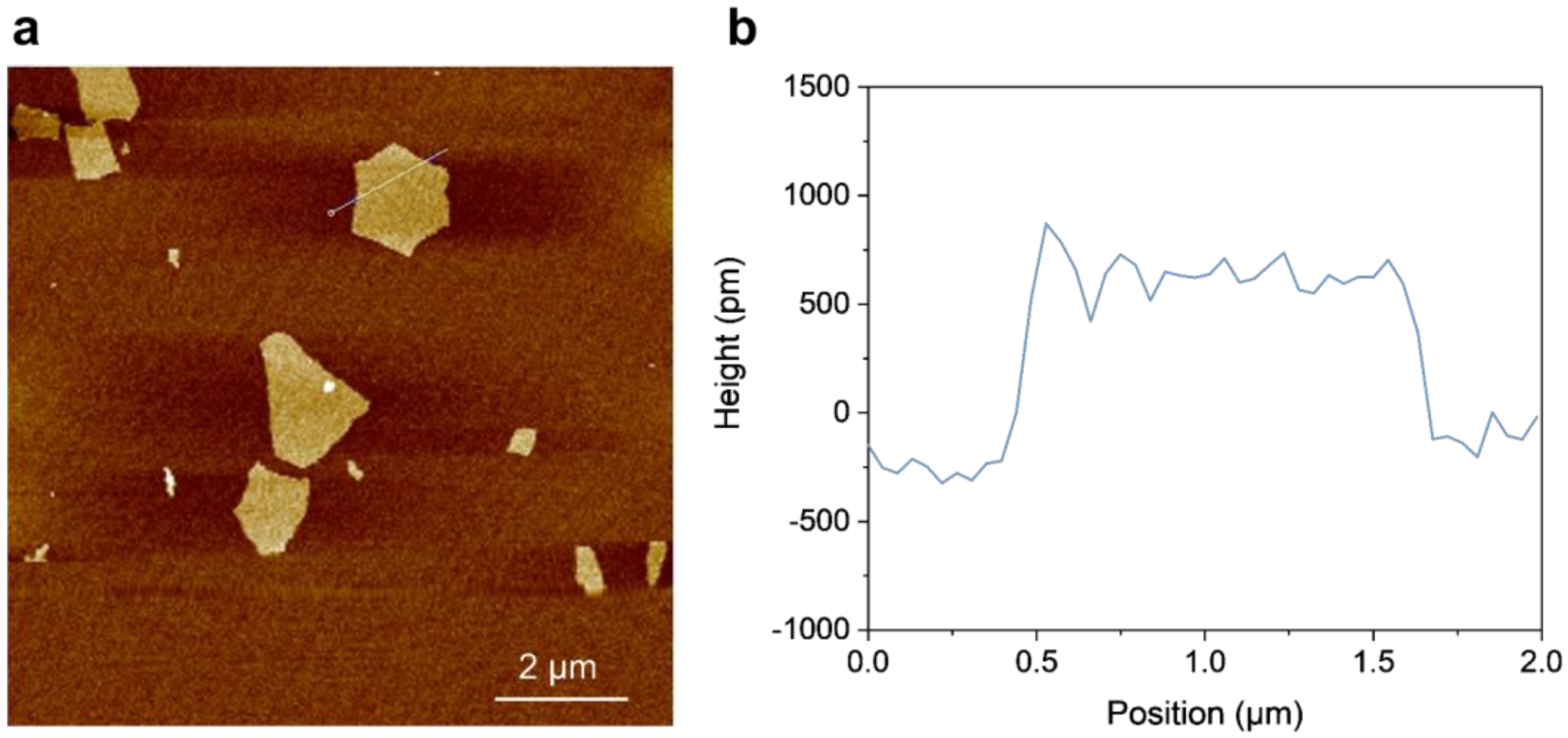

Supplementary Figure 3. (a) AFM image of GO nanosheets and (b) the corresponding height.

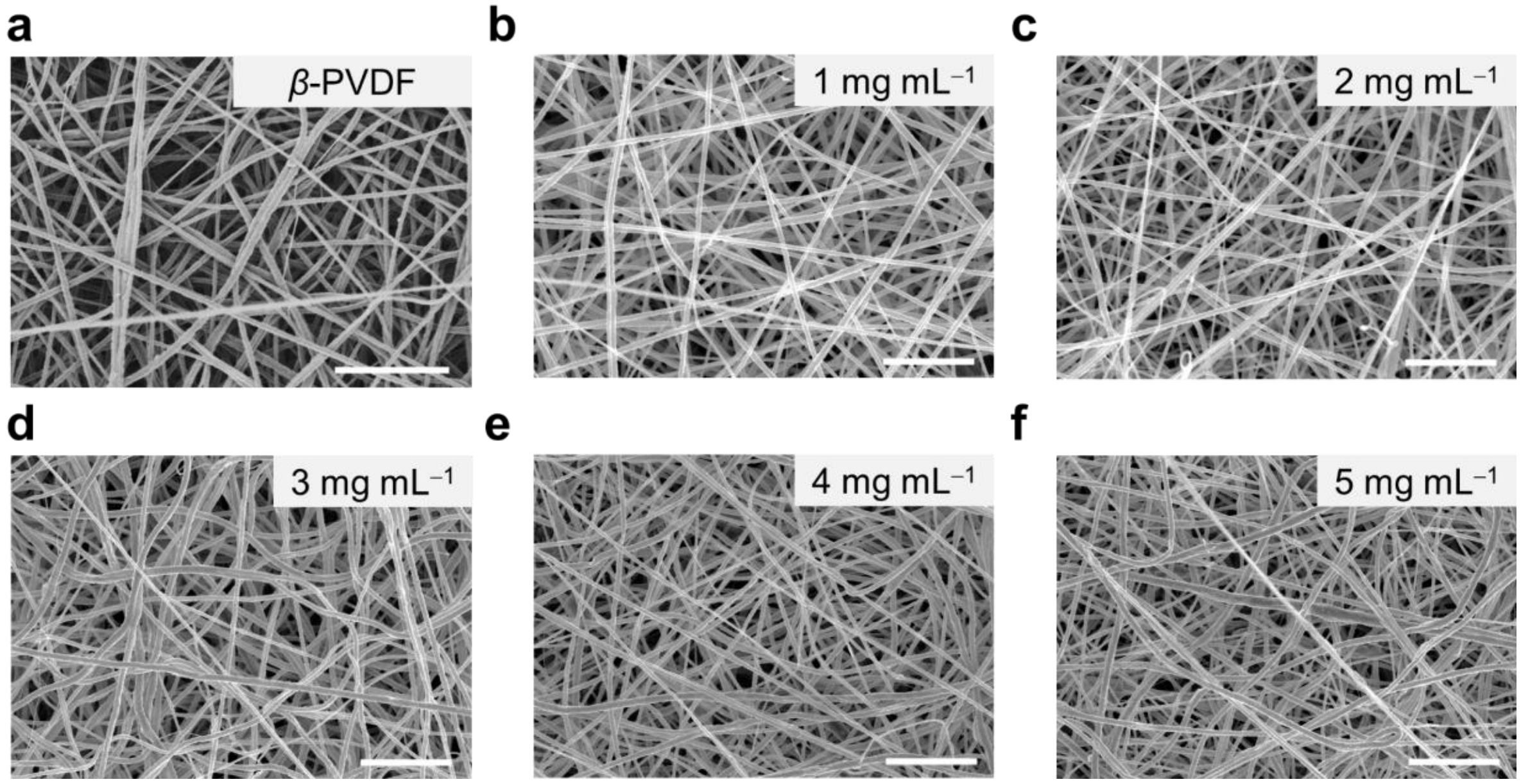

Supplementary Figure 4. SEM images of (a) PVDF and (b-f) graphene membranes fabricated using various GO concentrations: (b) 1 mg mL^{-1}; (c) 2 mg mL^{-1}; (d) 3 mg mL^{-1}; (e) 4 mg mL^{-1}; and (f) 5 mg mL^{-1}, respectively. The scale bar represents 5 μm.

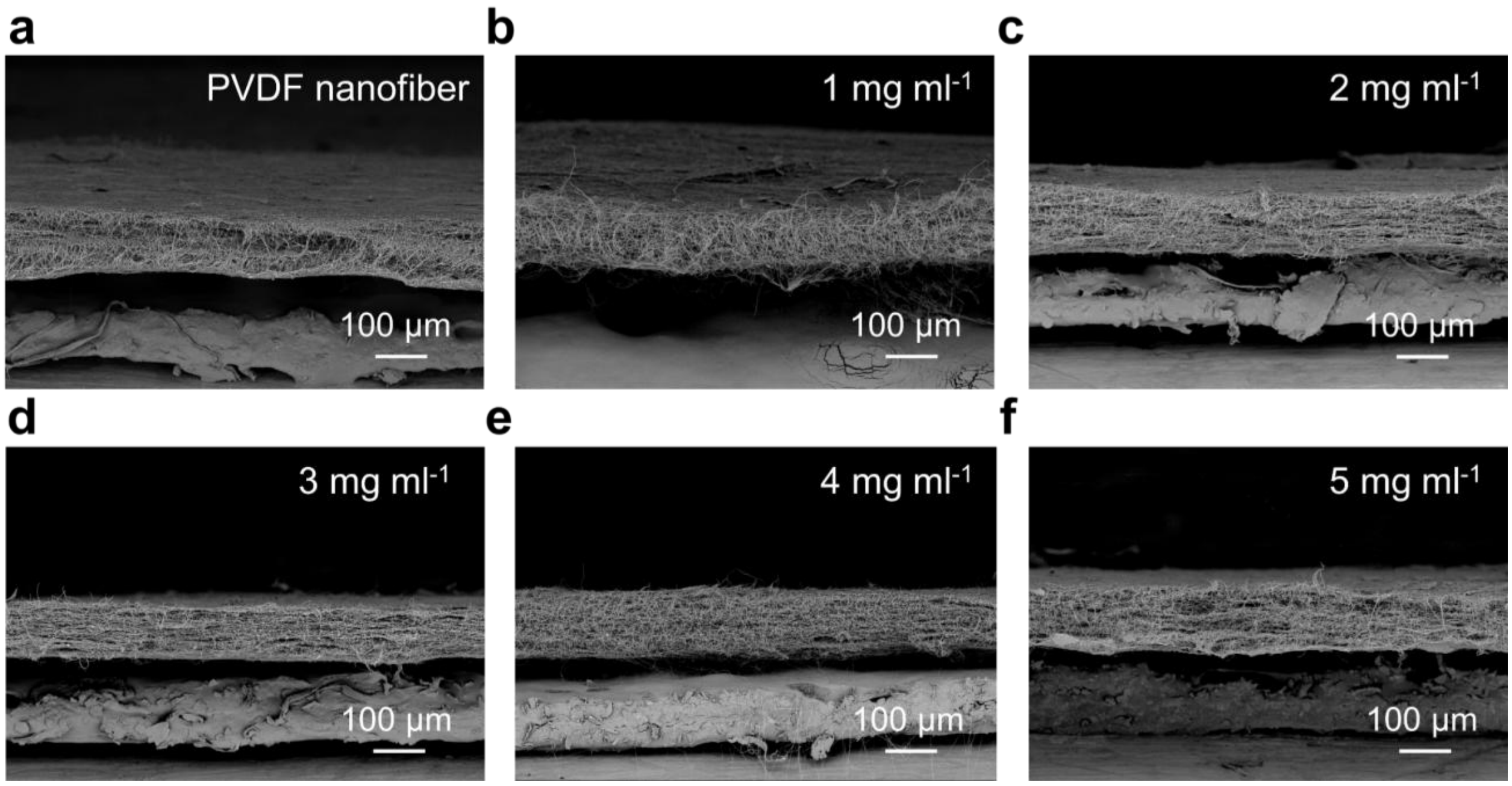

Supplementary Figure 5. Cross-sectional SEM images of pristine PVDF and graphene membranes fabricated using various GO concentrations: (b) 1 mg mL^{-1}; (c) 2 mg mL^{-1}; (d) 3 mg mL^{-1}; (e) 4 mg mL^{-1}; and (f) 5 mg mL^{-1}, respectively.

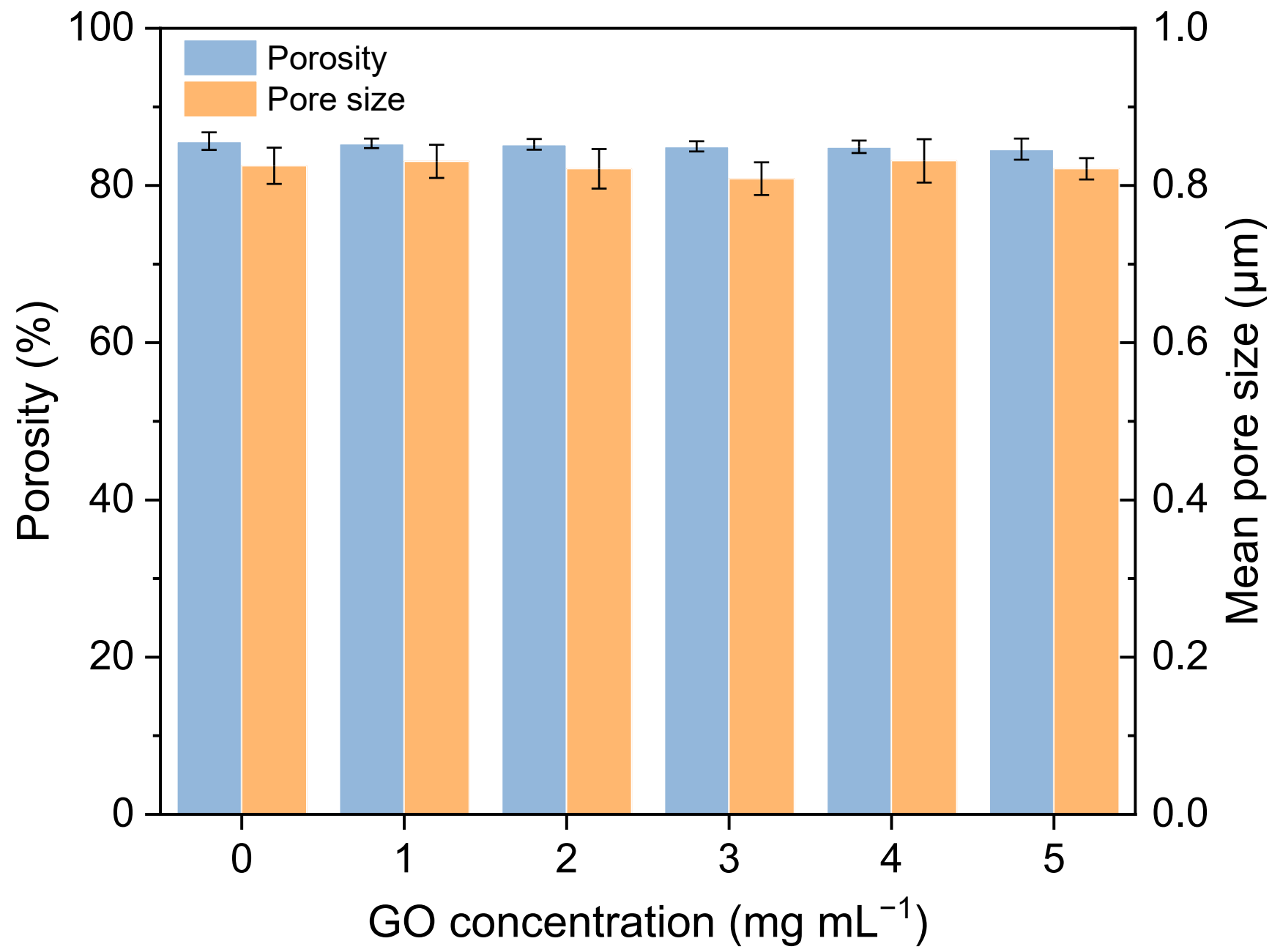

Supplementary Figure 6. Porosity and mean pore size of PVDF and graphene membranes fabricated using various GO concentrations.

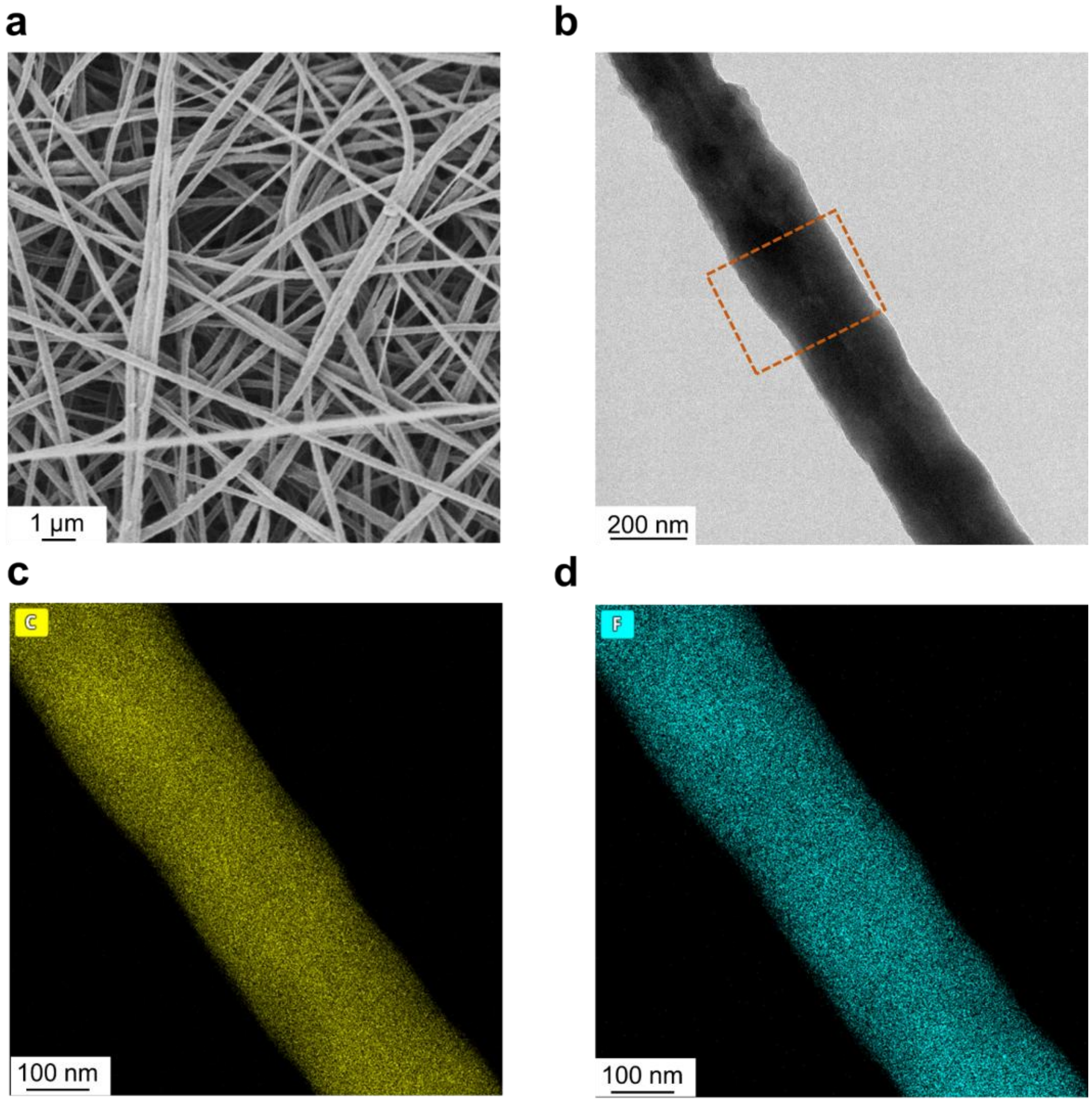

Supplementary Figure 7. (a) SEM, (b) TEM images, (c) and (d) EDX mapping of PVDF nanofibers. The dashed box in (b) indicates the area chosen for (c) and (d).

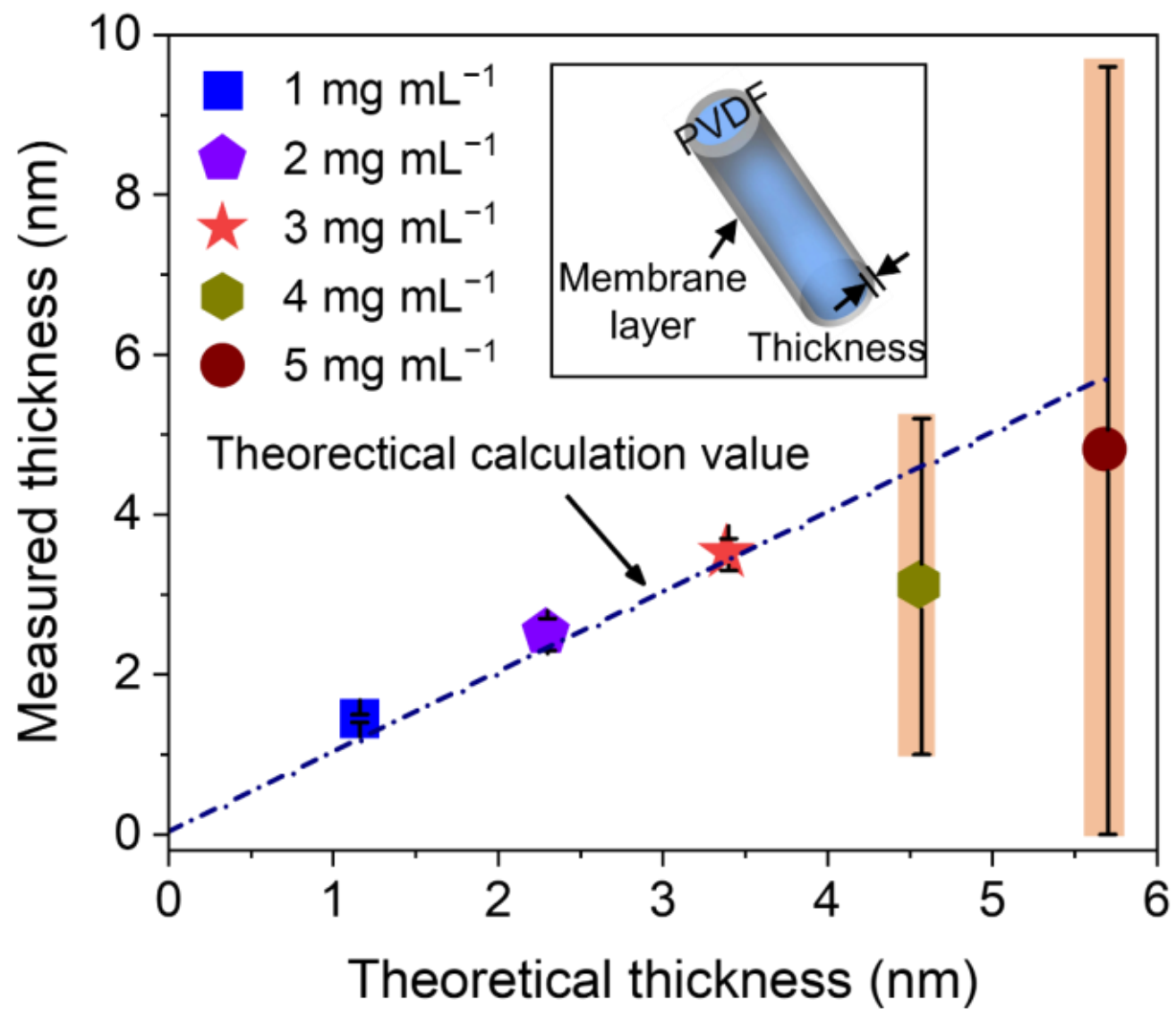

Supplementary Figure 8. Experimental and theoretical thickness of the membrane layer on the PVDF nanofiber. The theoretical thickness was estimated using a simplified core-shell model (inset).

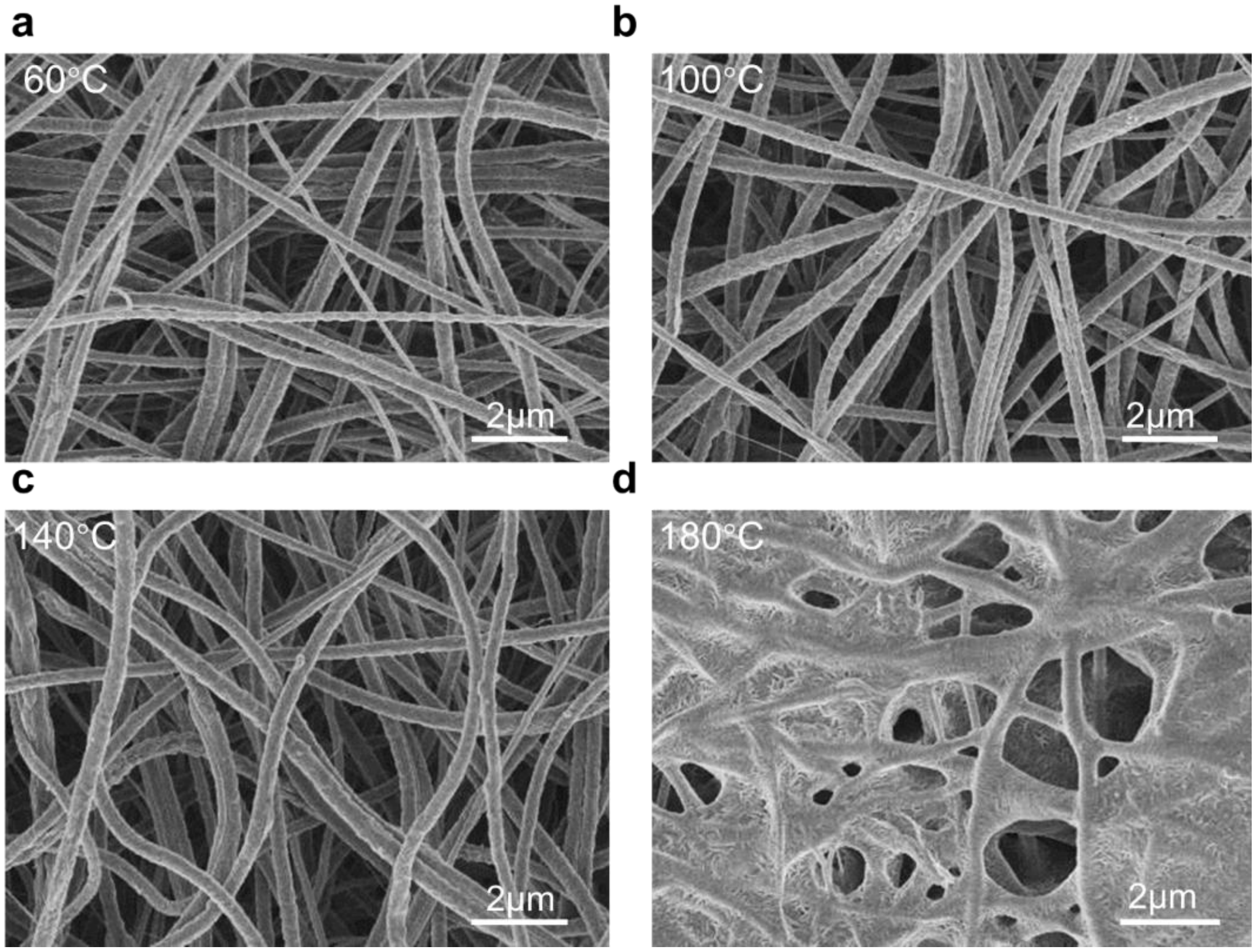

Supplementary Figure 9. SEM images of the graphene membranes treated at different temperatures: (a) 60°C ; (b) 100°C; (c) 140°C; (d) 180°C.

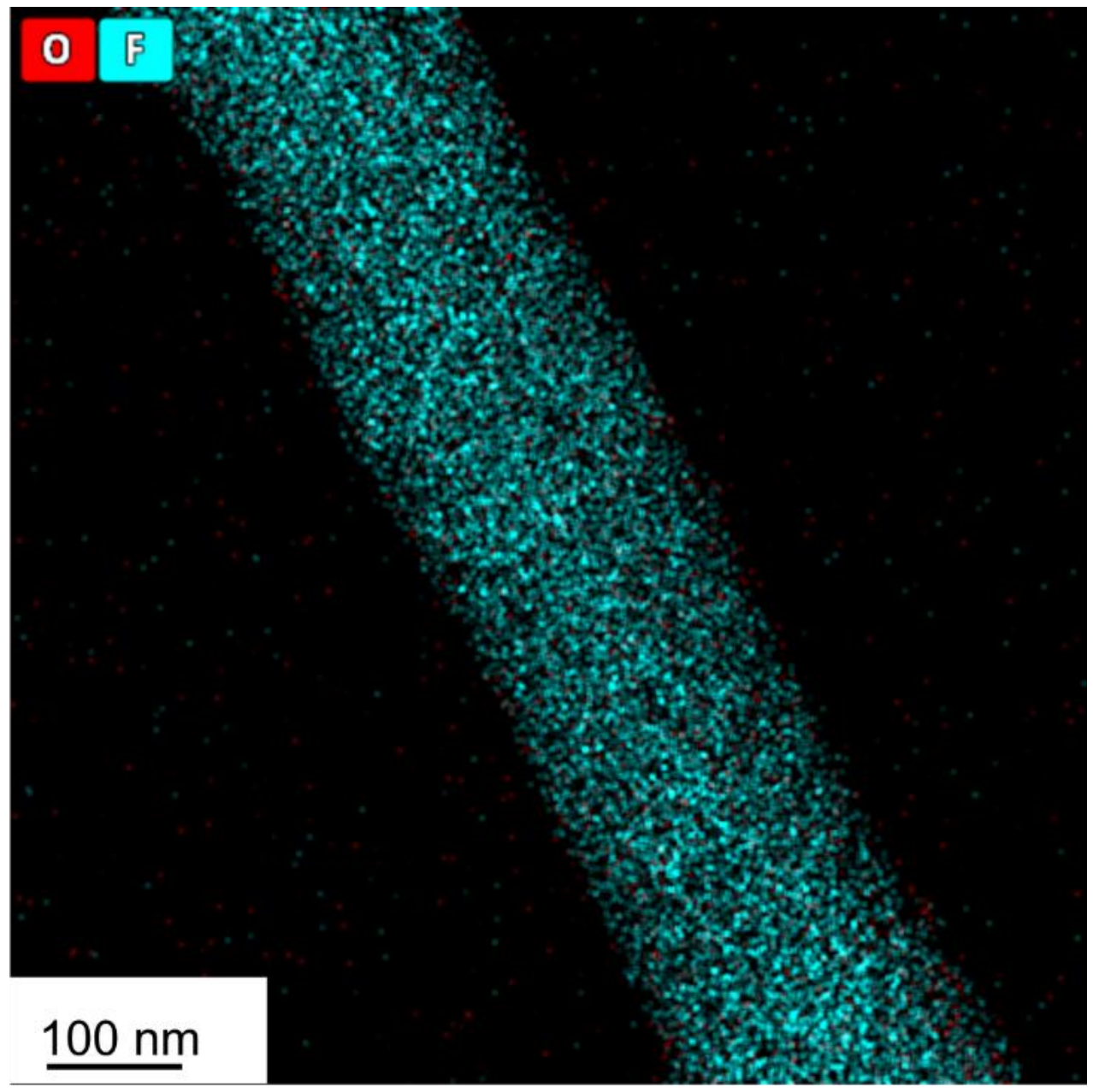

Supplementary Figure 10. EDX elemental mapping of a graphene nanofiber from the membrane fabricated with a GO concentration of 3 mg mL^{-1}, an applied electric field of 15 kV, and thermal reduction temperature of 140°C.

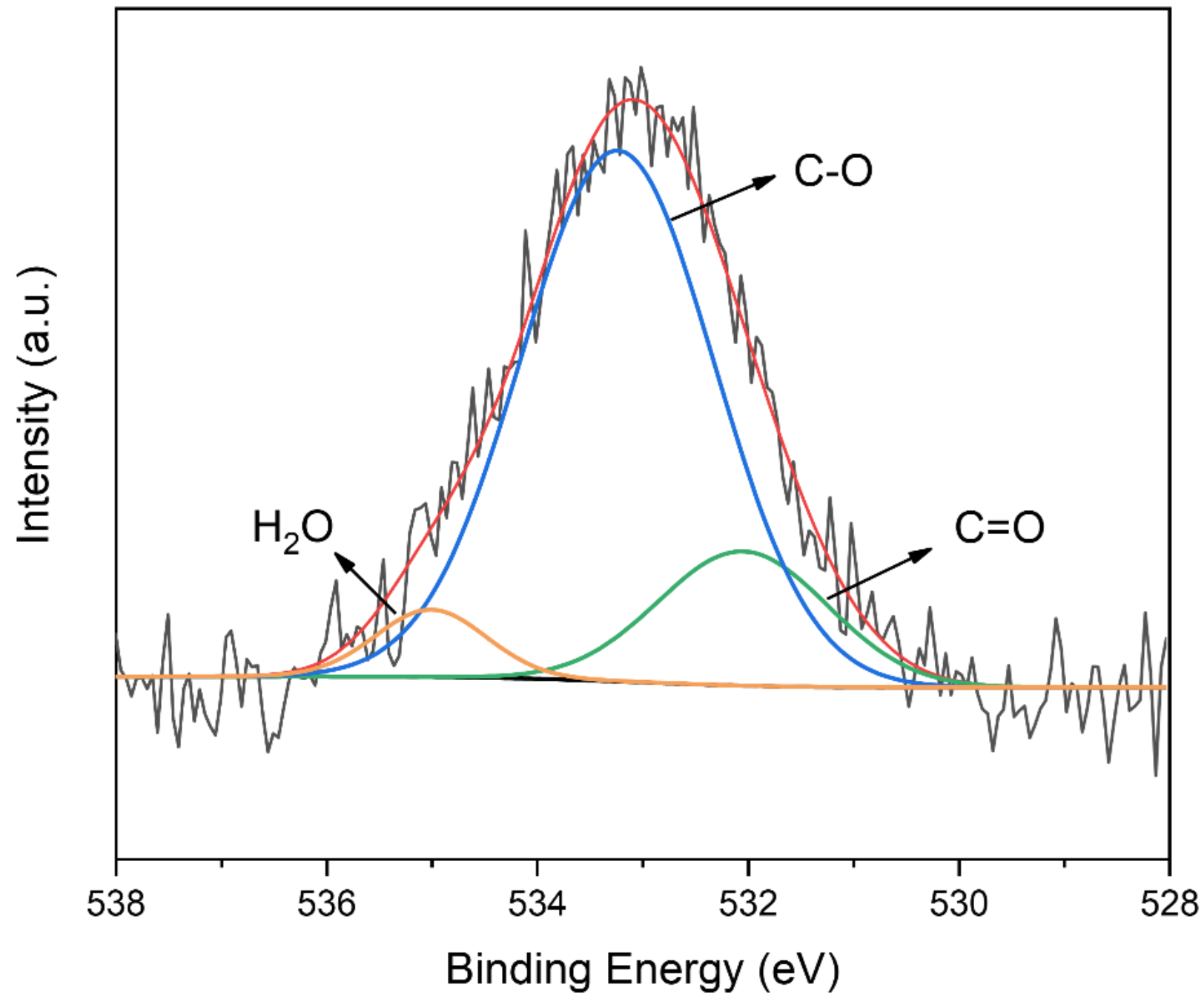

Supplementary Figure 11. XPS O1s spectra of the thermal reduced GO membrane. From the XPS results, we obtained the oxygen content of ~2%, which is calculated by normalizing its peak area to sensitivity factors and total detected elements.

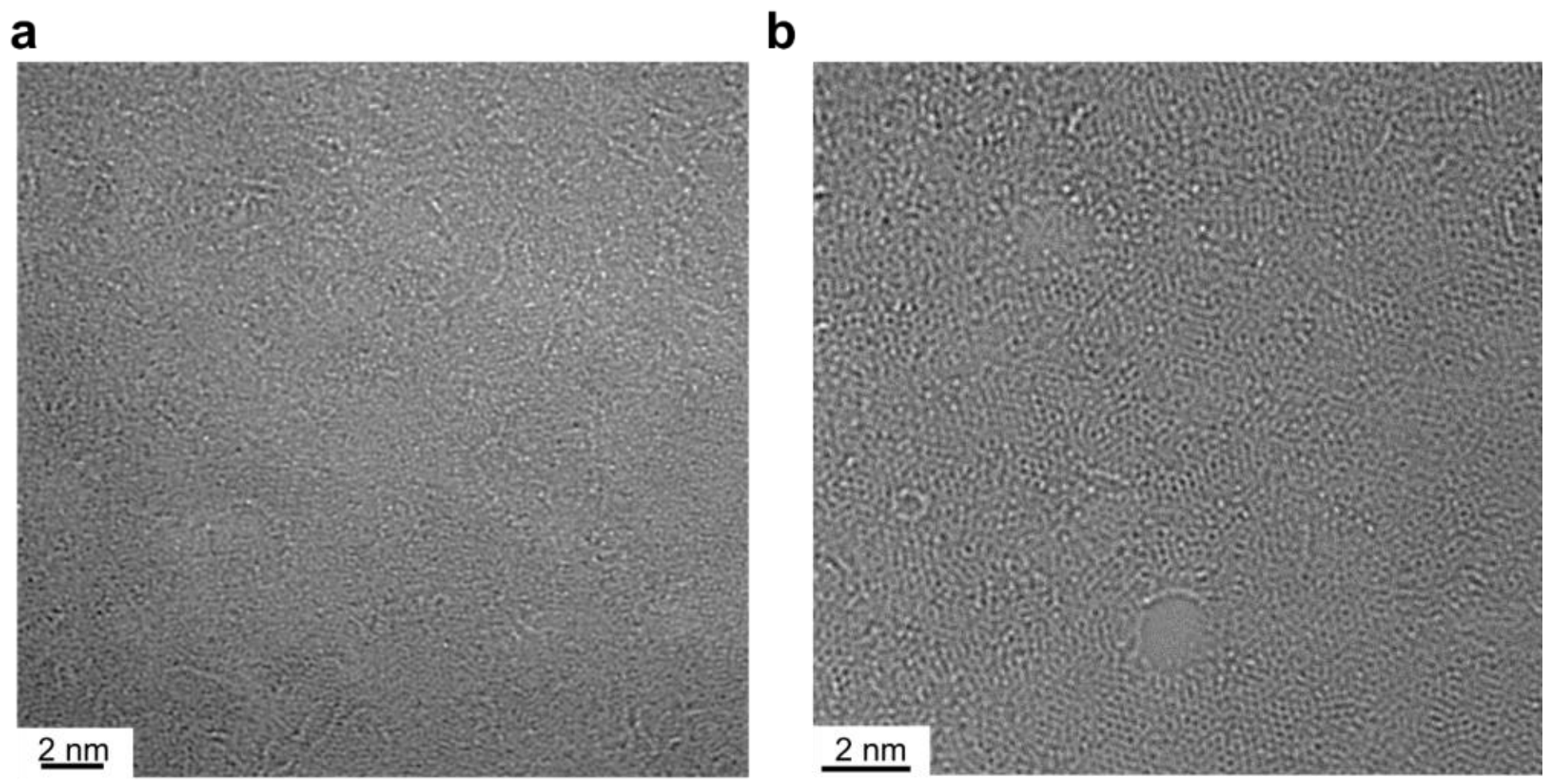

Supplementary Figure 12. Aberration-corrected transmission electron microscopy (AC-TEM) images of (a) GO nanosheets and (b) thermal reduced GO nanosheets.

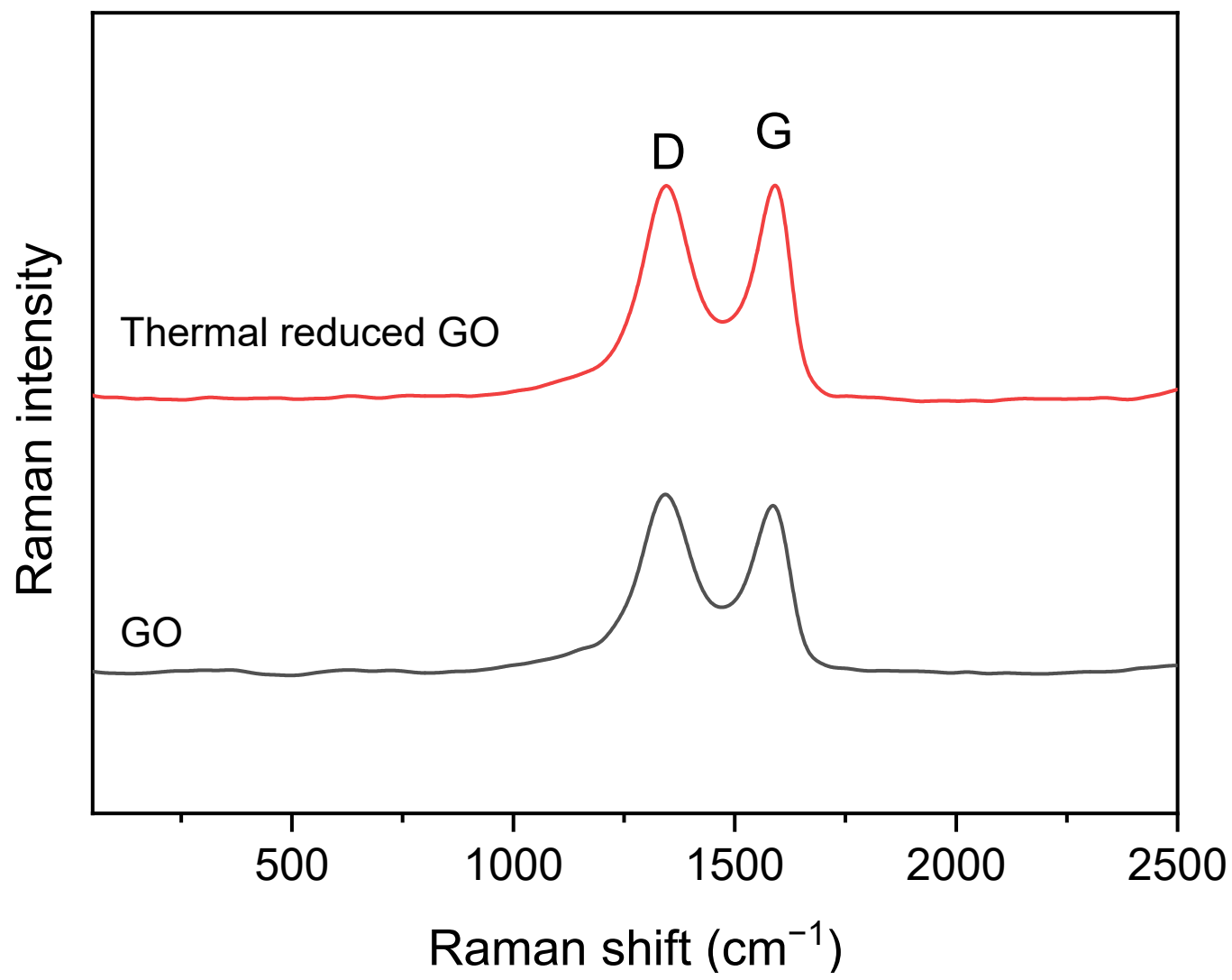

Supplementary Figure 13. Raman spectrums of GO and thermal reduced GO membranes.

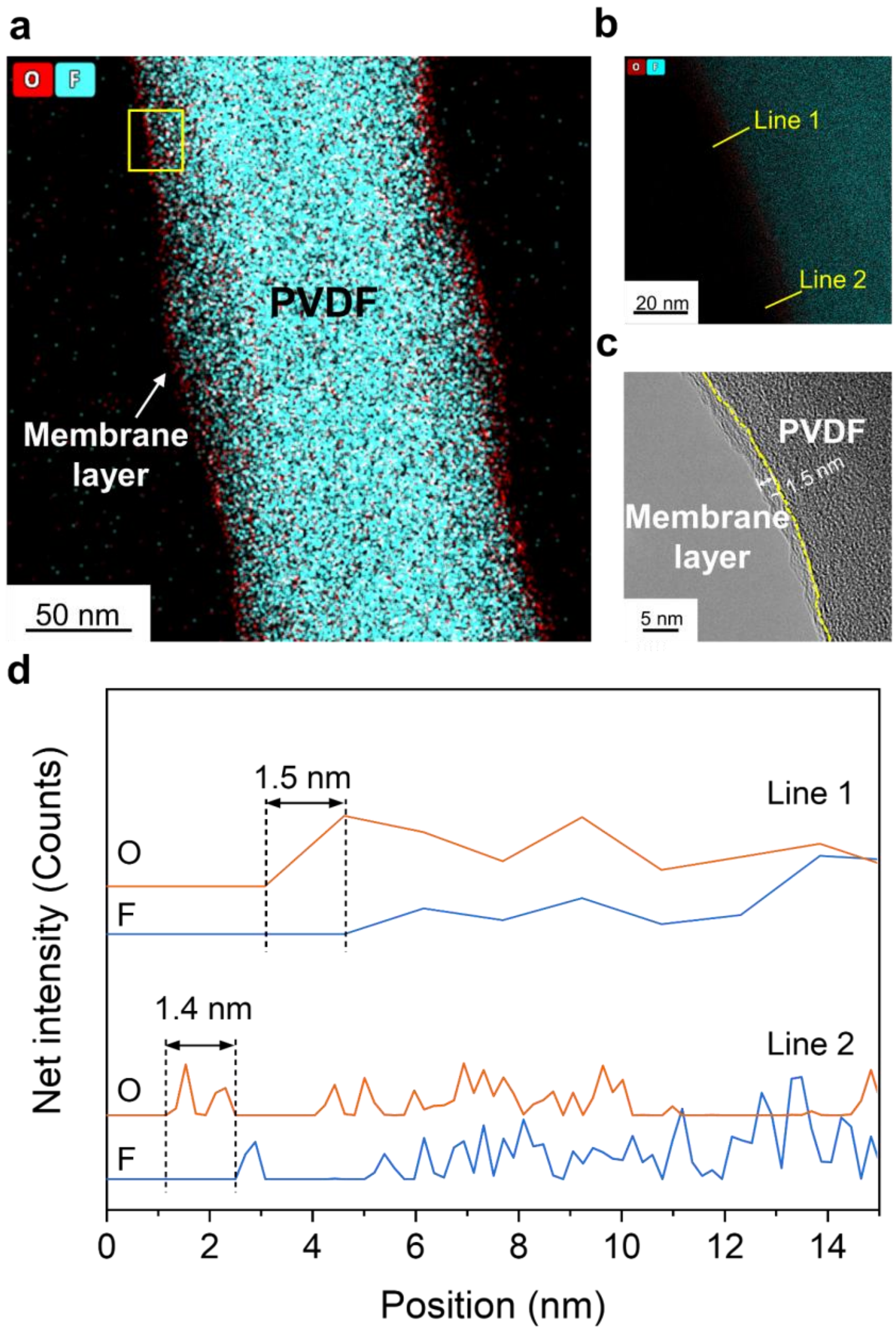

Supplementary Figure 14. TEM characterizations of GO membrane fabricated with a GO concentration of 1 mg mL^{-1} and an electric field of 15 kV. (a-b) EDX mapping, with the yellow box in (a) indicating the area chosen for (b). c, HRTEM image of the enlarged GO layer. (d) Distribution of O and F elements along line 1 and line 2 in panel (b), with the yellow line in (b) representing the detection depth.

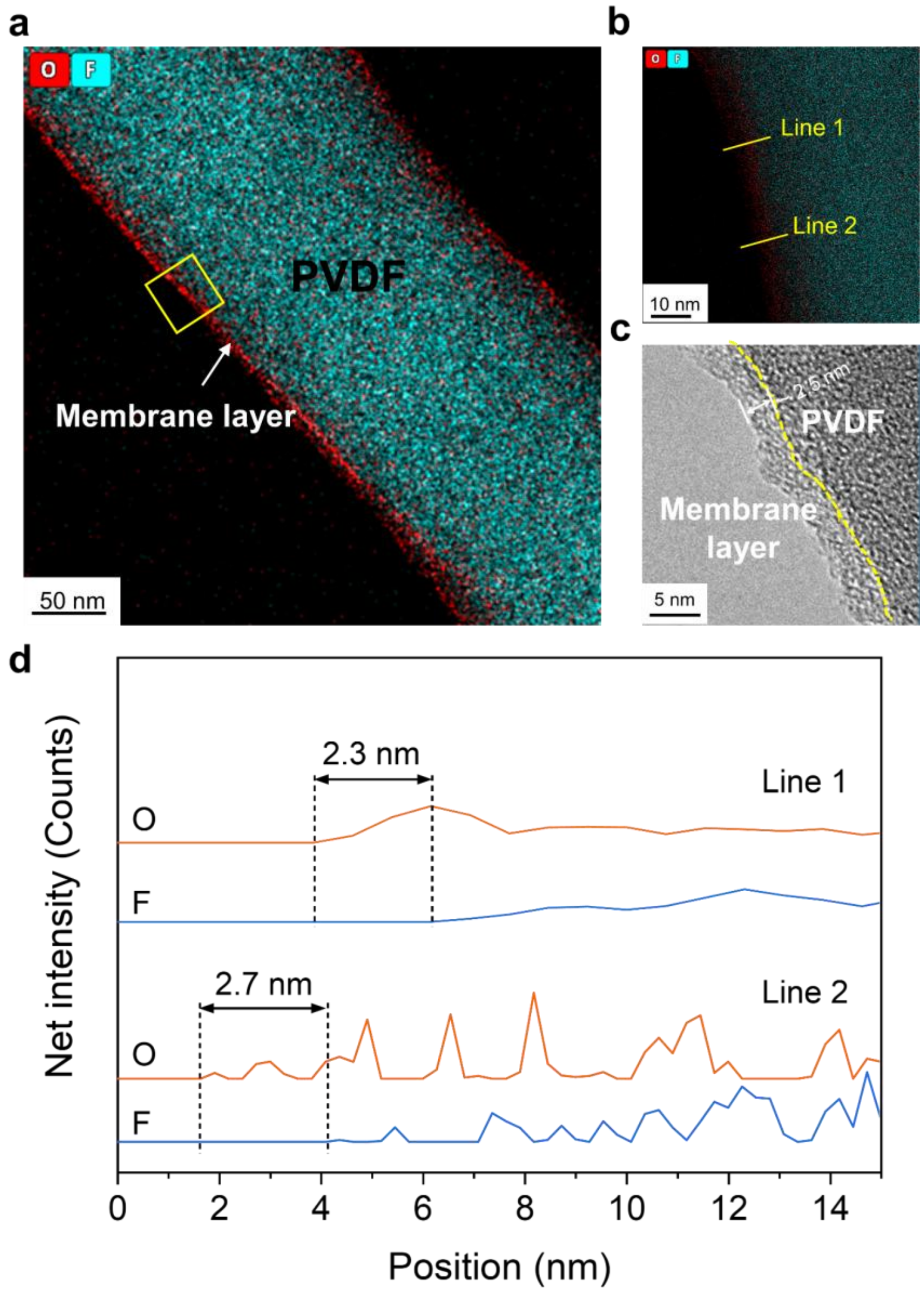

Supplementary Figure 15. TEM characterizations of GO membrane fabricated with a GO concentration of 2 mg mL^{-1} and an electric field of 15 kV. (a-b) EDX mapping, with the yellow box in (a) indicating the area selected for (b). (c) HRTEM image of the enlarged GO layer. (d) Distribution of O and F elements along lines 1 and 2 in panel (b), with the yellow line in (b) representing the detection depth.

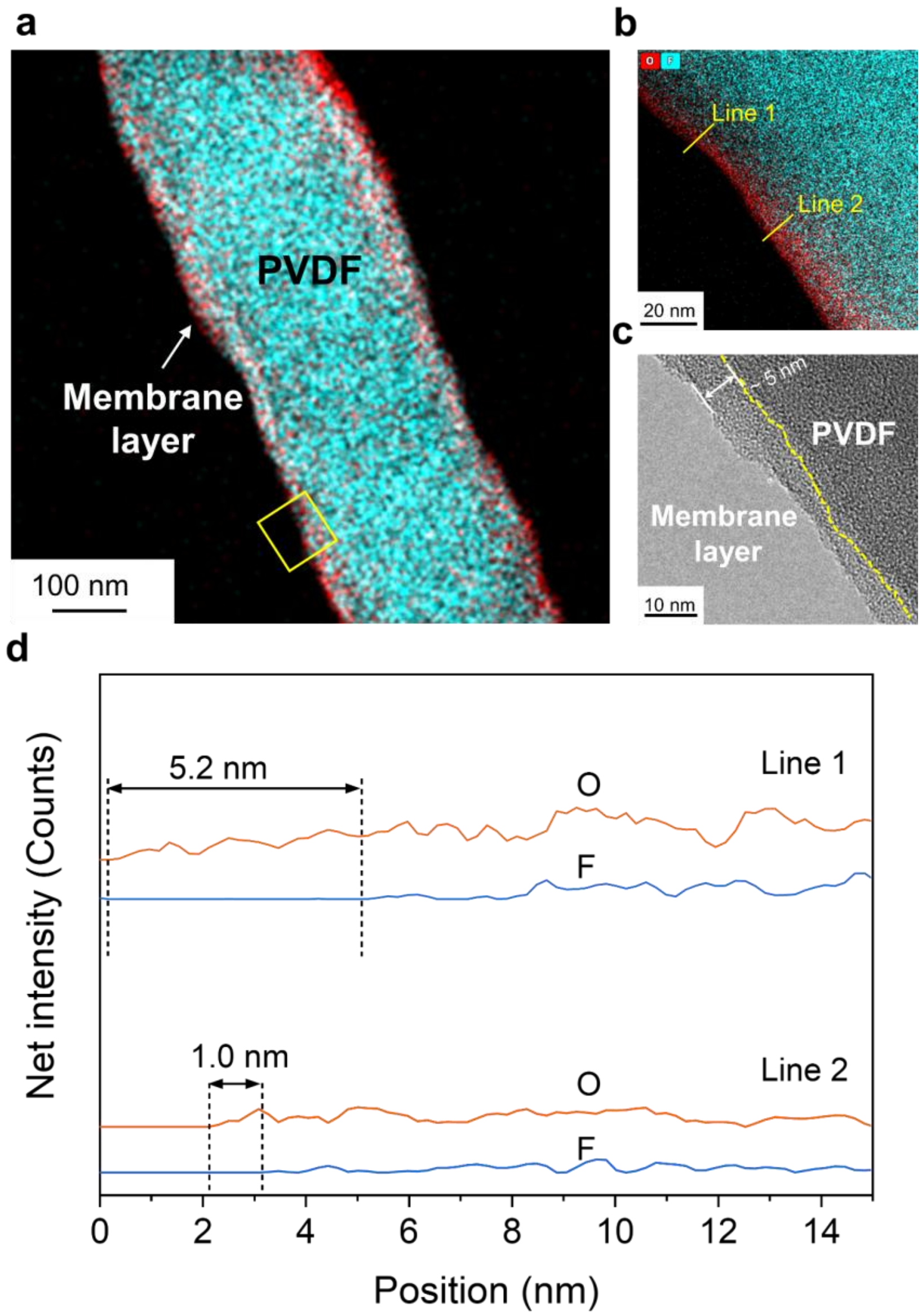

Supplementary Figure 16. TEM characterizations of GO membrane fabricated with a GO concentration of 4 mg mL^{-1} and an electric field of 15 kV. (a-b) EDX mapping, with the yellow box in (a) indicating the area selected for (b). (c) HRTEM image of the enlarged GO layer. (d) Distribution of O and F elements along lines 1 and 2 in panel (b), with the yellow line in (b) representing the detection depth.

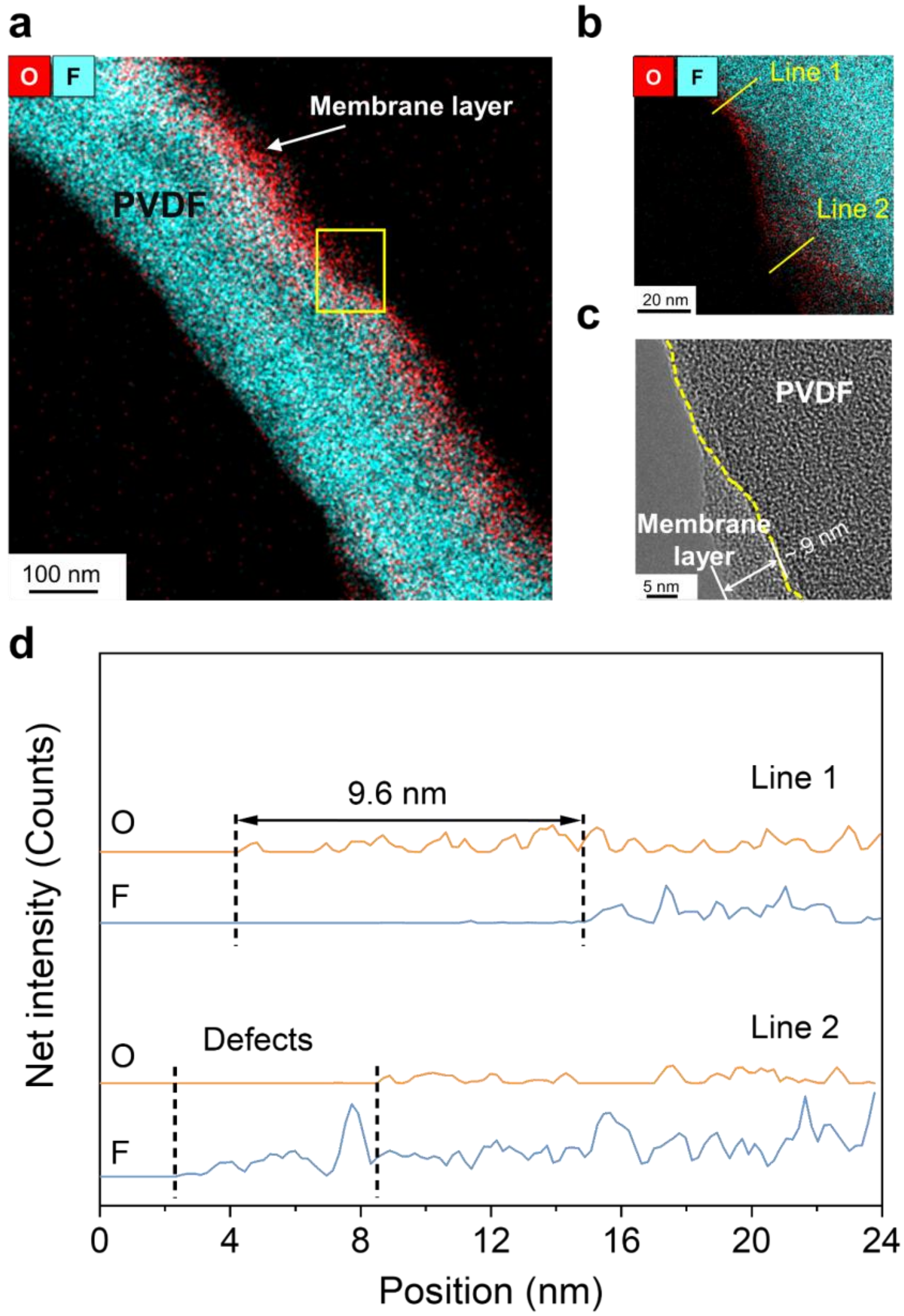

Supplementary Figure 17. TEM characterizations of GO membrane fabricated with a GO concentration of 5 mg mL^{-1} and an electric field of 15 kV. (a-b) EDX mapping, with the yellow box in (a) indicating the area selected for (b). (c) HRTEM image of the enlarged GO layer. (d) Distribution of O and F elements along lines 1 and 2 in panel (b), with the yellow line in (b) representing the detection depth.

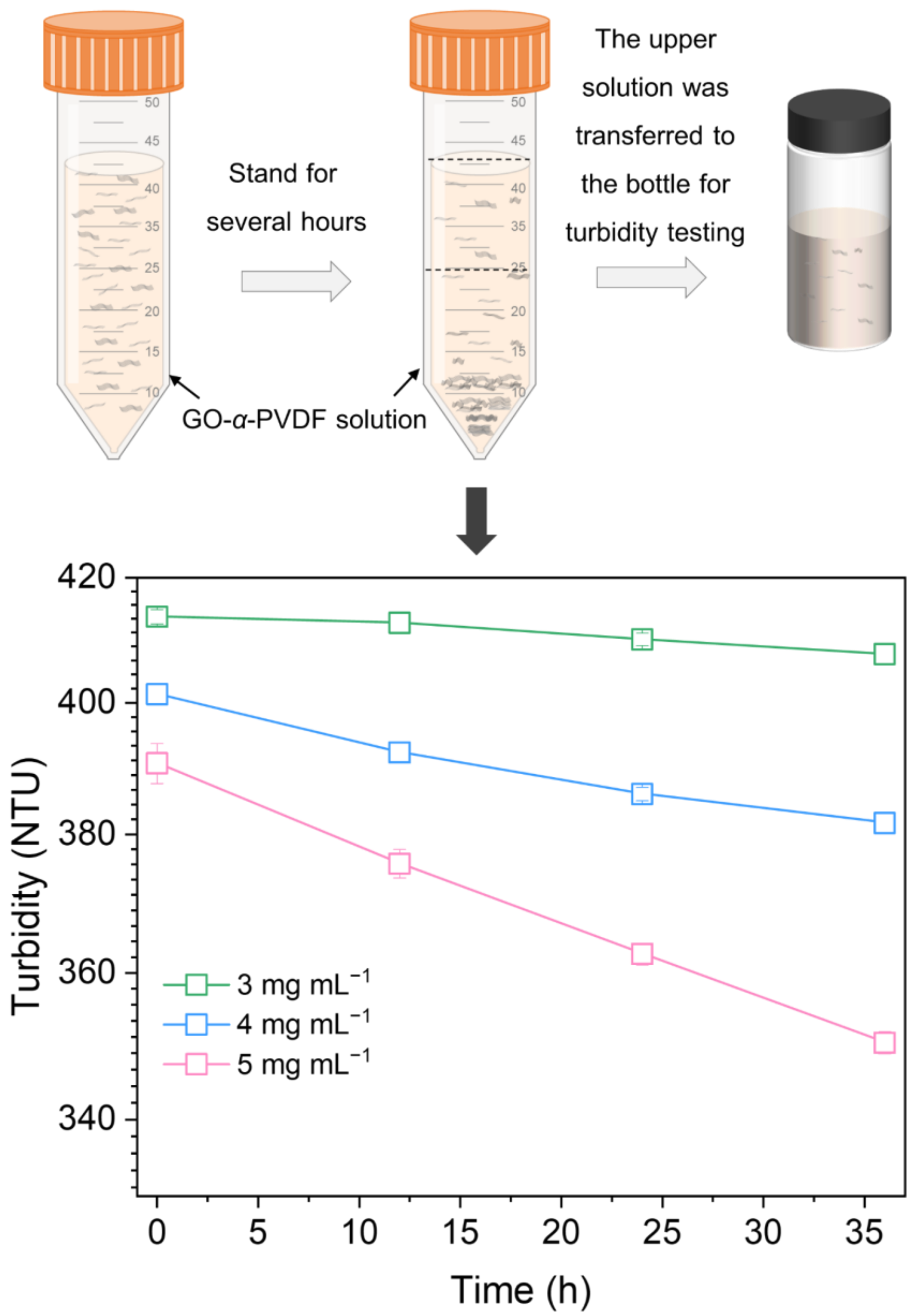

Supplementary Figure 18. Turbidity of GO-α-PVDF solution with GO concentrations of 3-5 mg mL^{-1} during standing for different time.

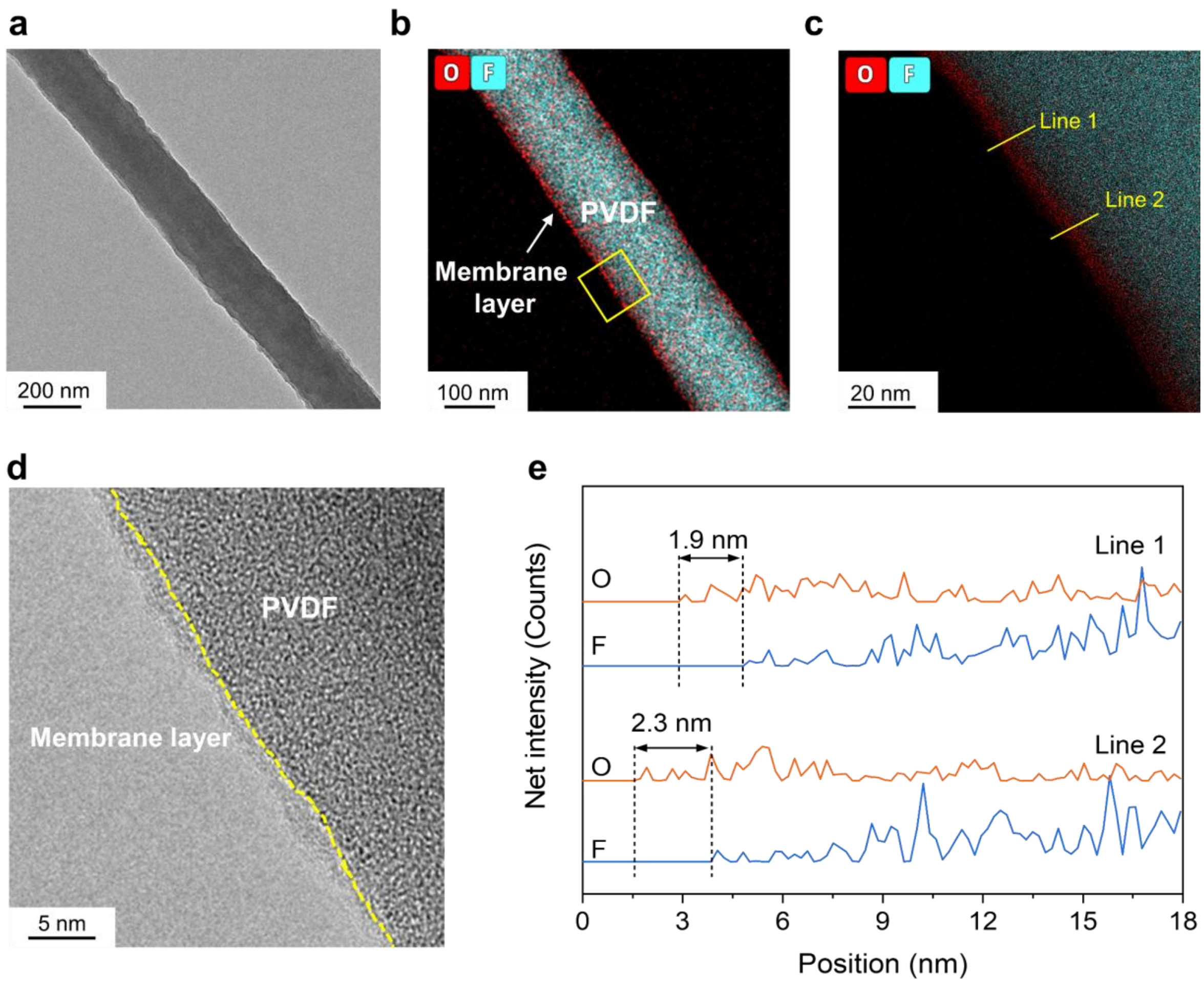

Supplementary Figure 19. TEM characterizations of GO membrane fabricated with a GO concentration of 3 mg mL^{-1} and an electric field of 10 kV. (a) HRTEM image of the GO membrane. (b-c) EDX mapping, with the yellow box in (b) indicating the area chosen for (c). (d) HRTEM image of the enlarged GO layer. (e) Distribution of O and F elements in lines 1 and 2 from panel (c). The yellow line in panel (c) represents the detection depth.

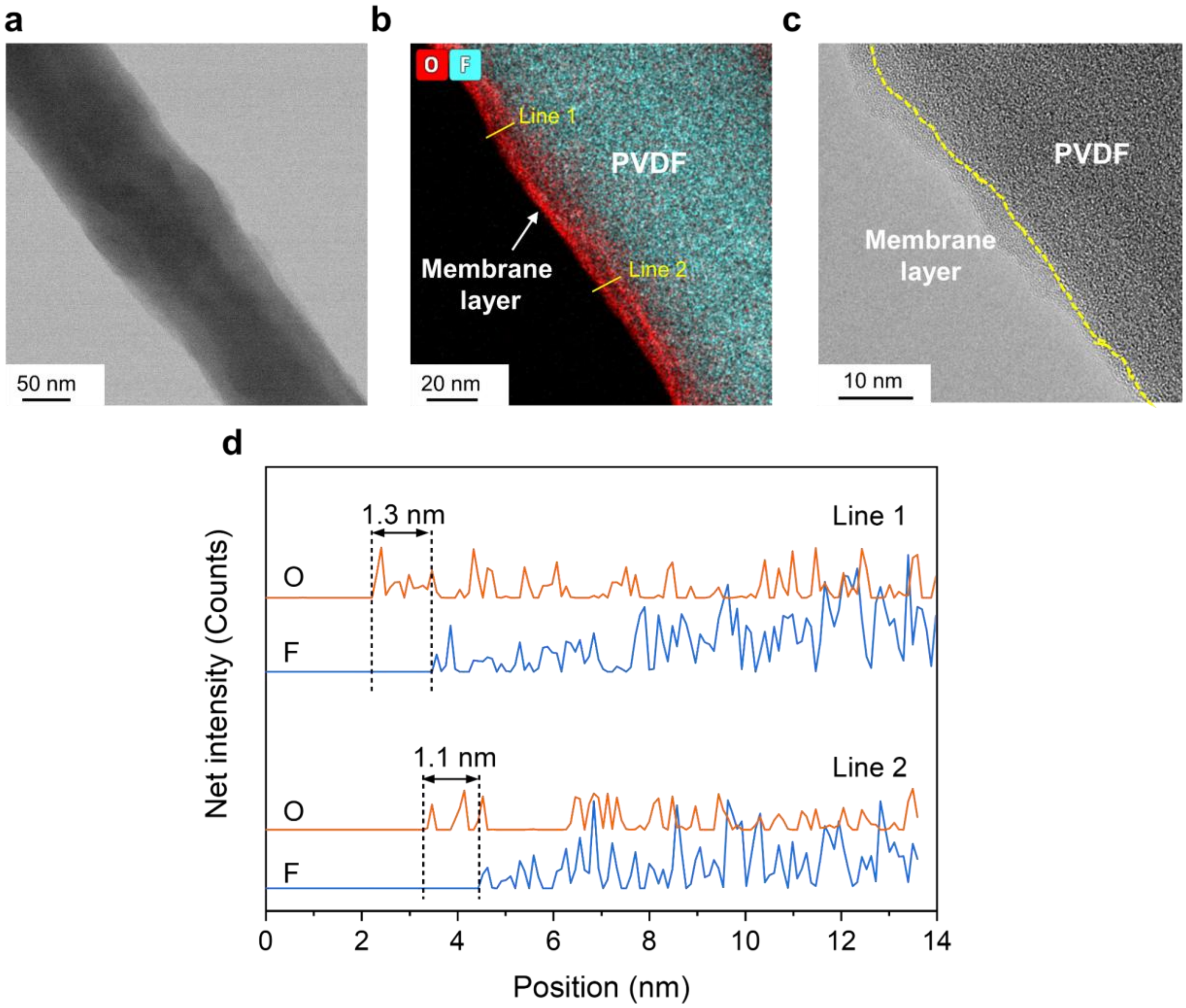

Supplementary Figure 20. TEM characterizations of GO membrane fabricated with a GO concentration of 3 mg mL^{-1} and an electric field of 20 kV. (a) HRTEM image of the GO membrane. (b) EDX mapping and (c) HRTEM image of the enlarged GO layer. (d) Distribution of O and F elements along lines 1 and 2 in panel (b). The yellow line in panel (b) represents the detection depth.

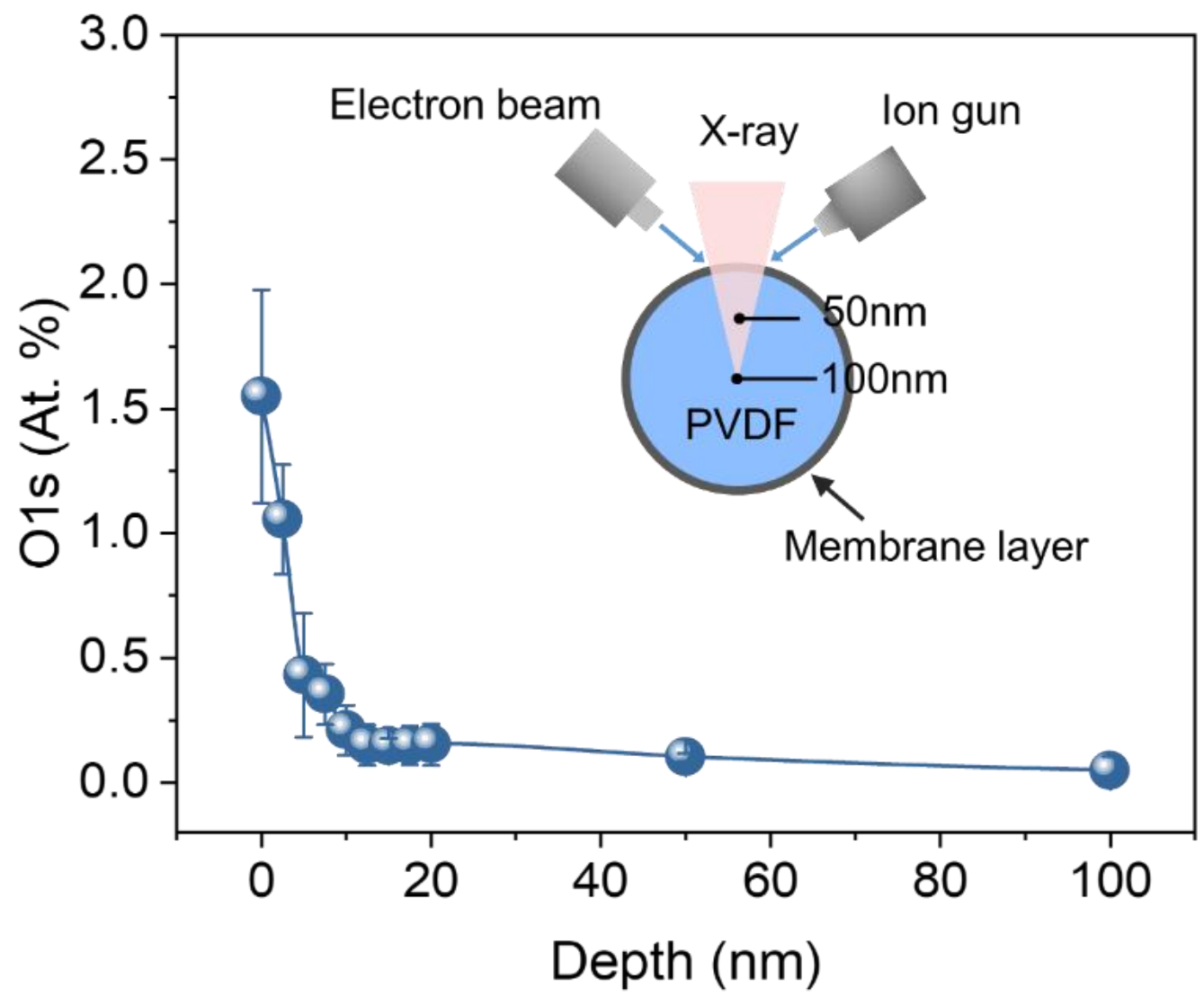

Supplementary Figure 21. Depth-dependent oxygen content of argon-ion-etched graphene membranes measured by XPS depth profiling.

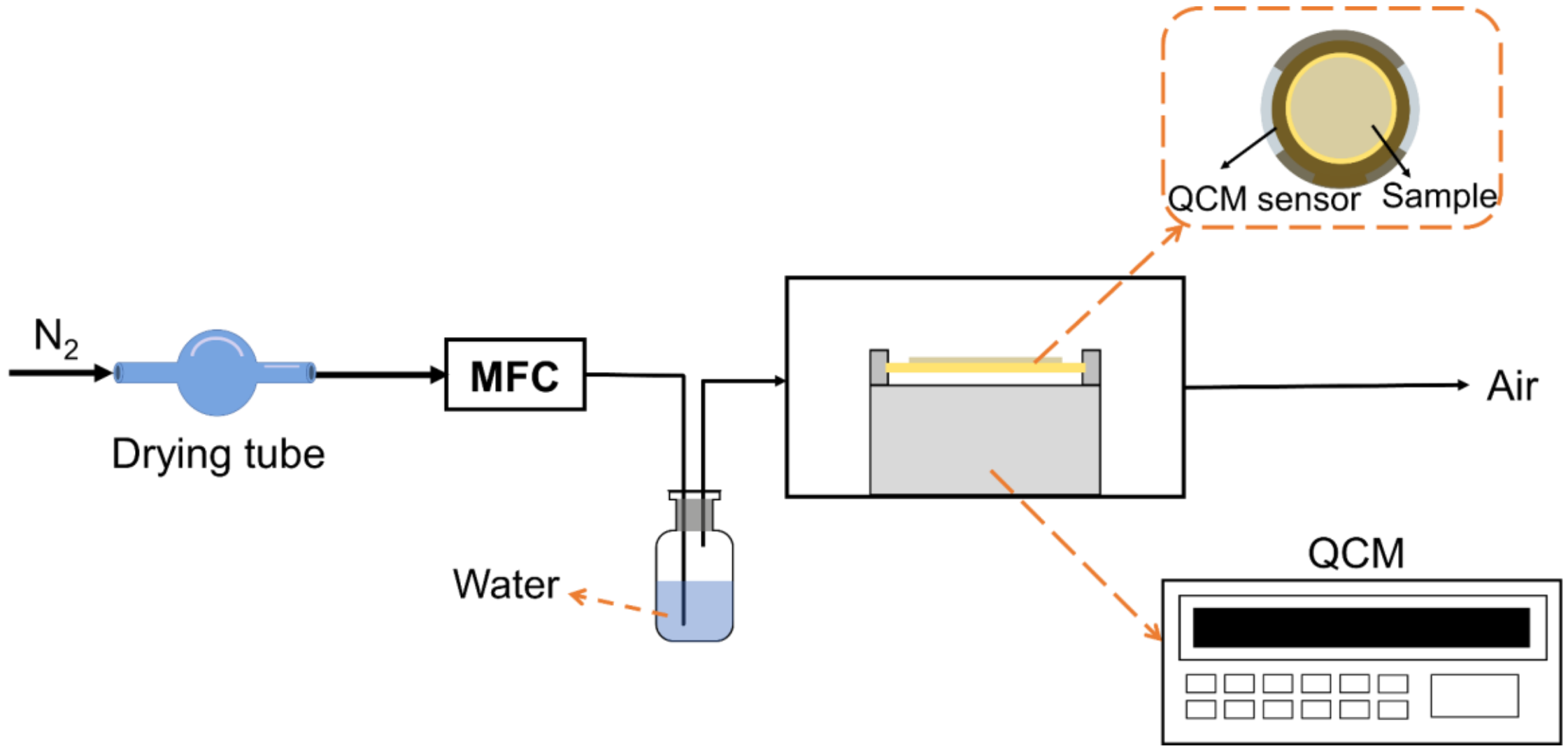

Supplementary Figure 22. Schematic diagram of a quartz crystal microbalance with dissipation (QCM-D) system.

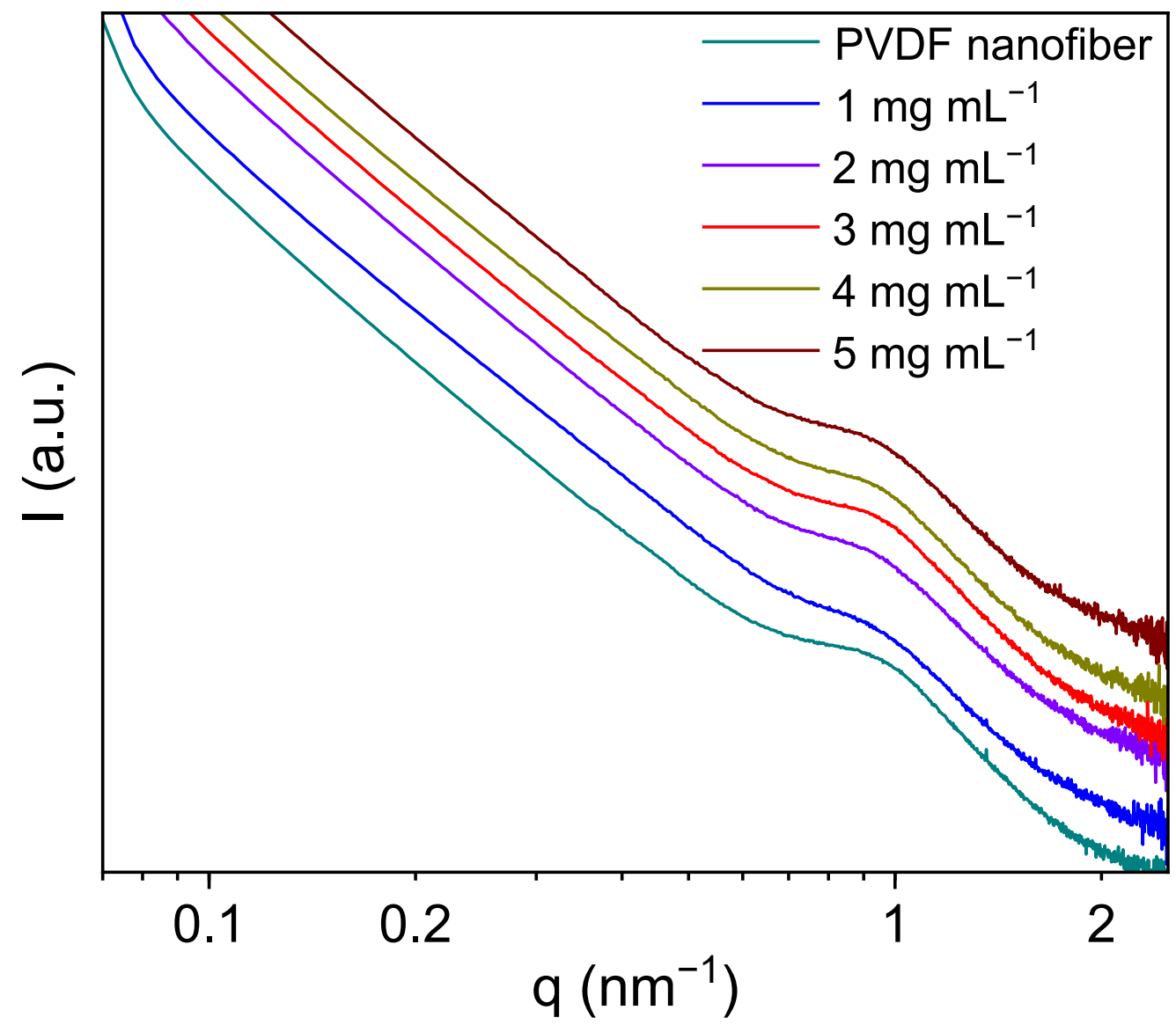

Supplementary Figure 23. One-dimensional SAXS spectra of PVDF and GO membranes with different GO concentrations.

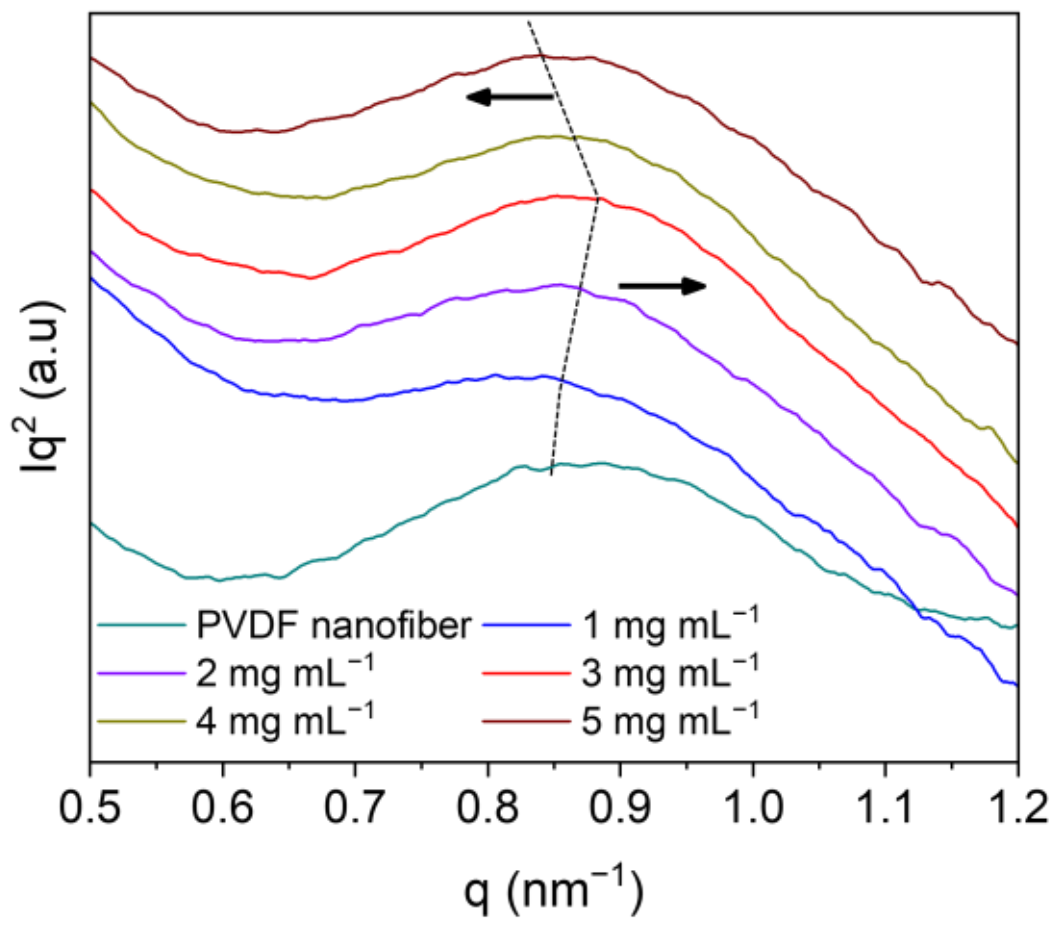

Supplementary Figure 24. Lorentz-corrected SAXs profile of PVDF and GO membranes with different GO concentrations.

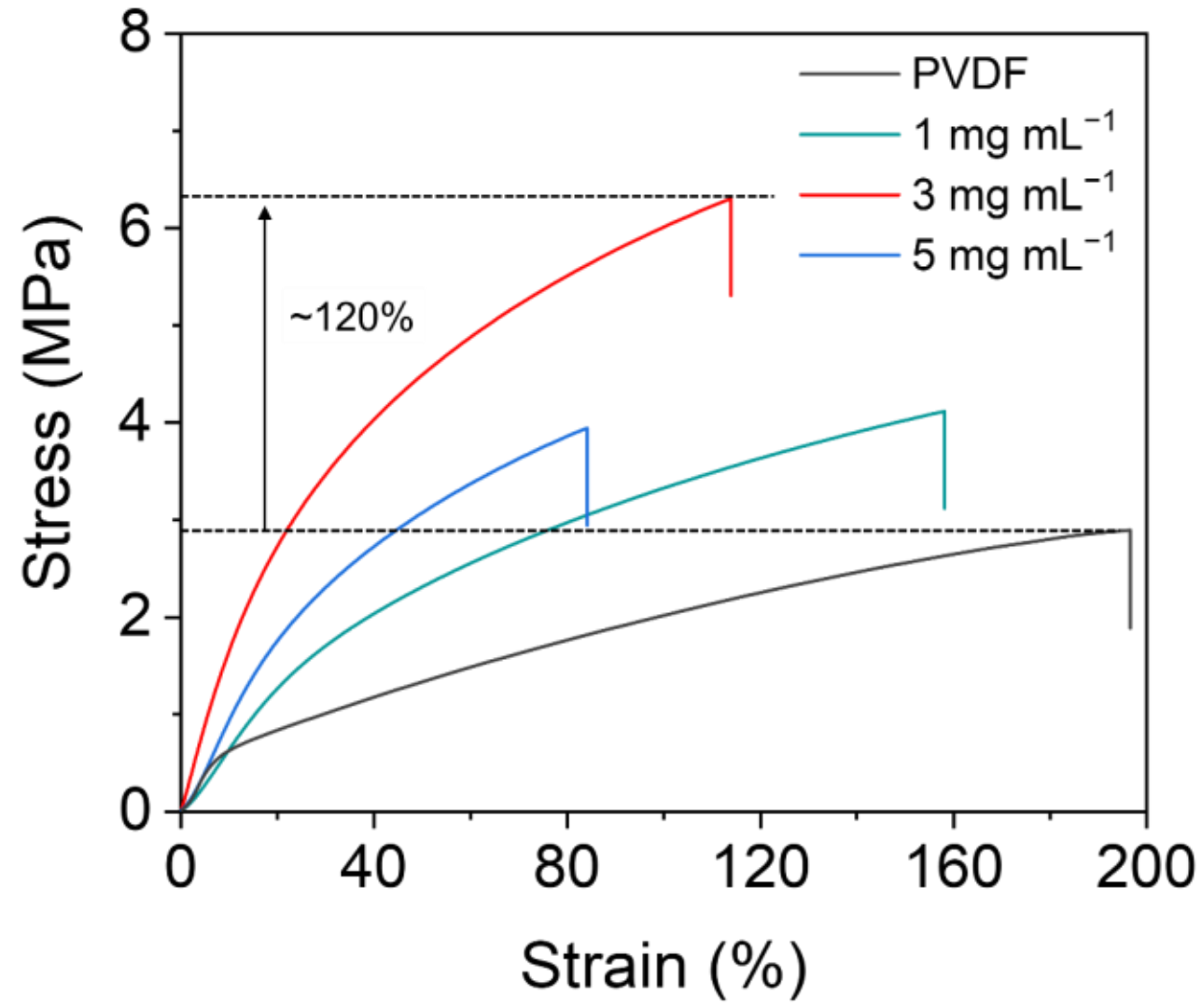

Supplementary Figure 25. Mechanical property of the PVDF membrane and graphene membranes fabricated with varying GO concentrations.

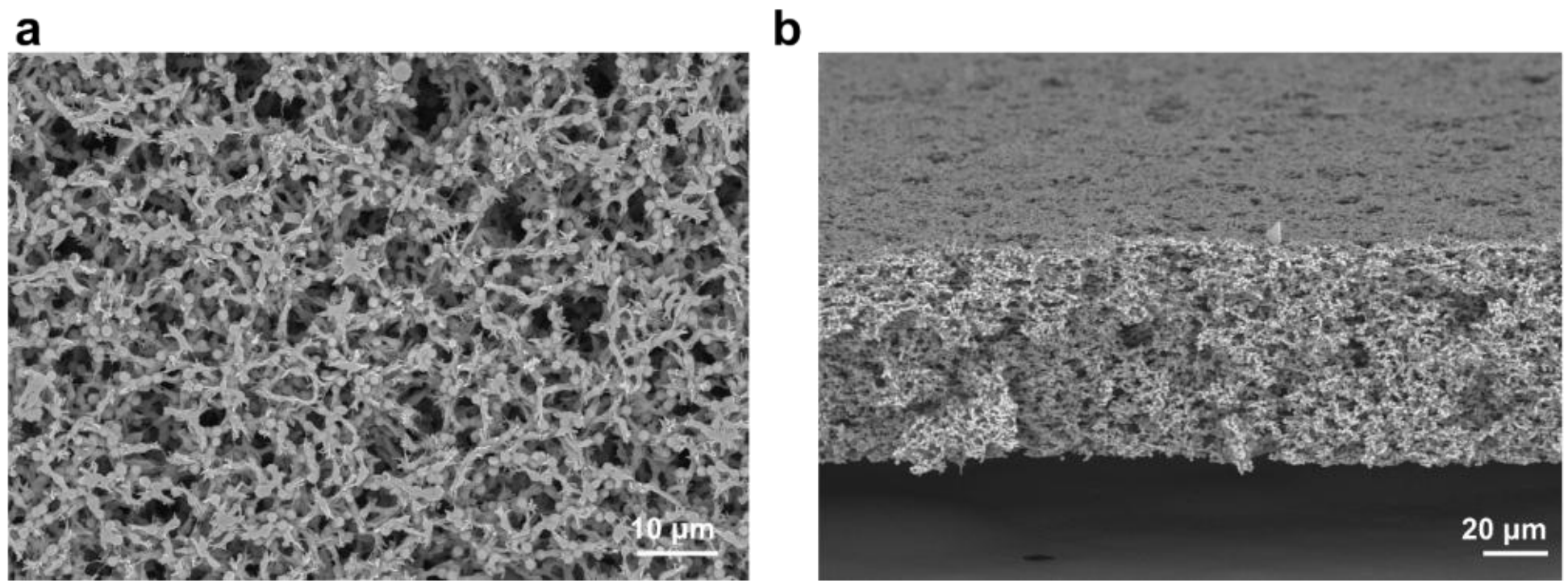

Supplementary Figure 26. SEM images of (a) surface and (b) cross-section of the graphene membrane prepared via the nonsolvent-induced phase separation (NIPS) method.

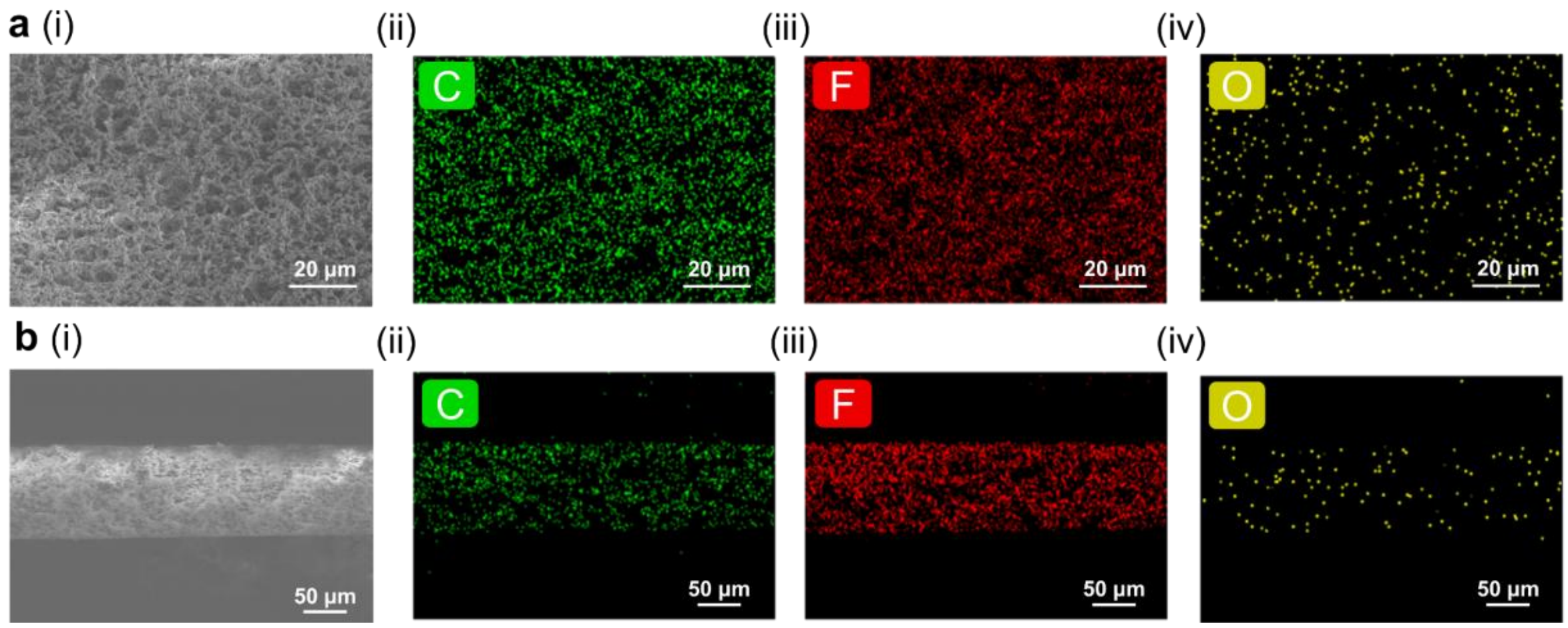

Supplementary Figure 27. SEM images of the graphene-β-PVDF membrane prepared by the nonsolvent-induced phase separation (NIPS) method: (a, i) surface view and (b, i) cross-sectional view. Panels (ii–iv) show the corresponding EDX elemental mappings: (ii) carbon (C), (iii) fluorine (F), and (iv) oxygen (O).

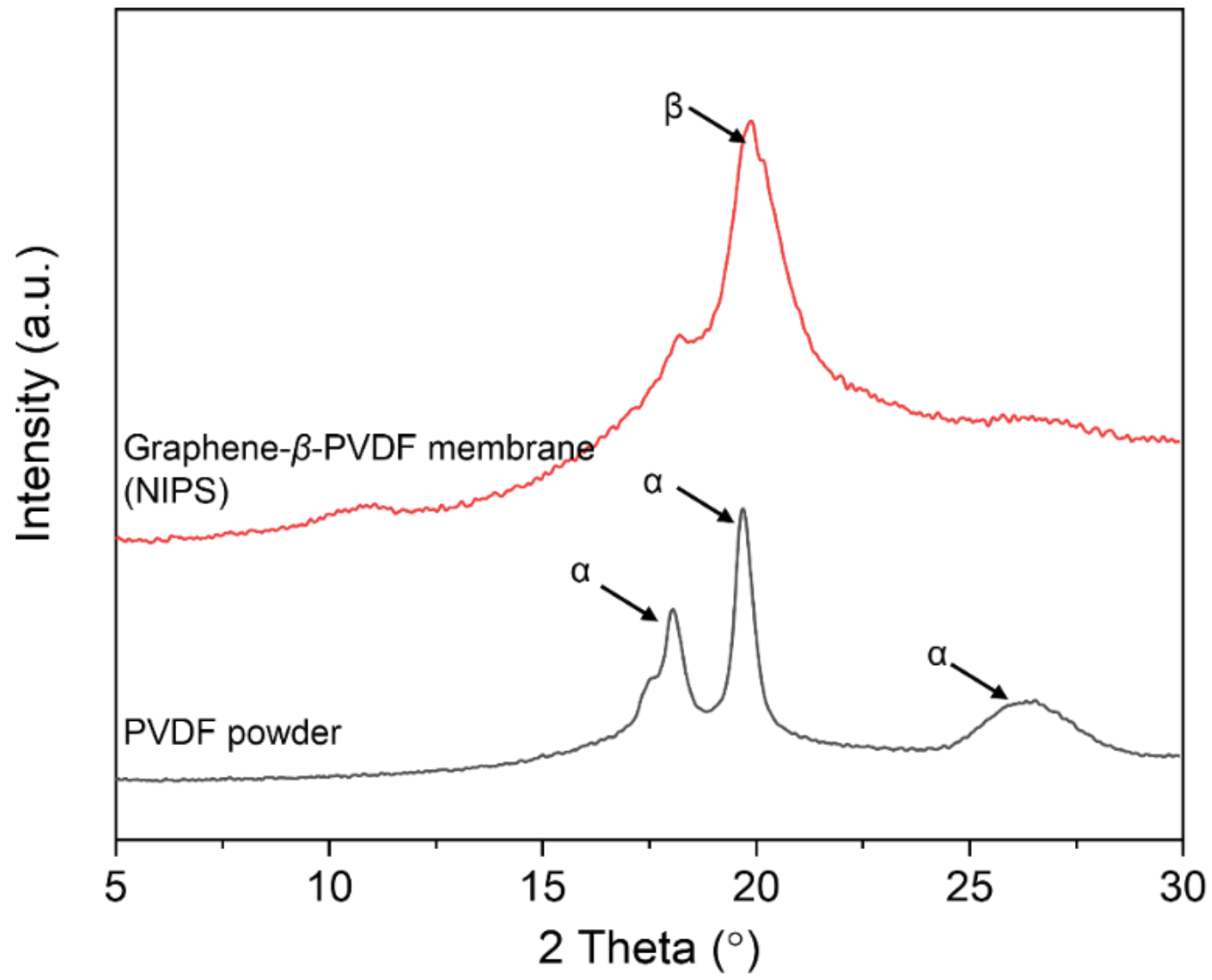

Supplementary Figure 28. XRD patterns of PVDF powder and the graphene-*β*-PVDF membrane prepared via the nonsolvent-induced phase separation (NIPS) method.

Although NIPS generated *β*-phase PVDF, the degree of chain rearrangement and orientation was insufficient to drive GO to completely segregate out of the PVDF chains. The chain packing difference induced by NIPS and electrospinning was reflected in our observation that DMF (a solvent of *α*-phase PVDF) can redisperse the NIPS-fabricated membrane, but cannot redisperse the electrospun membrane after 1 h immersion (Supplementary Figure 29), further confirming that GO nanosheets remained embedded within the polymer matrix.

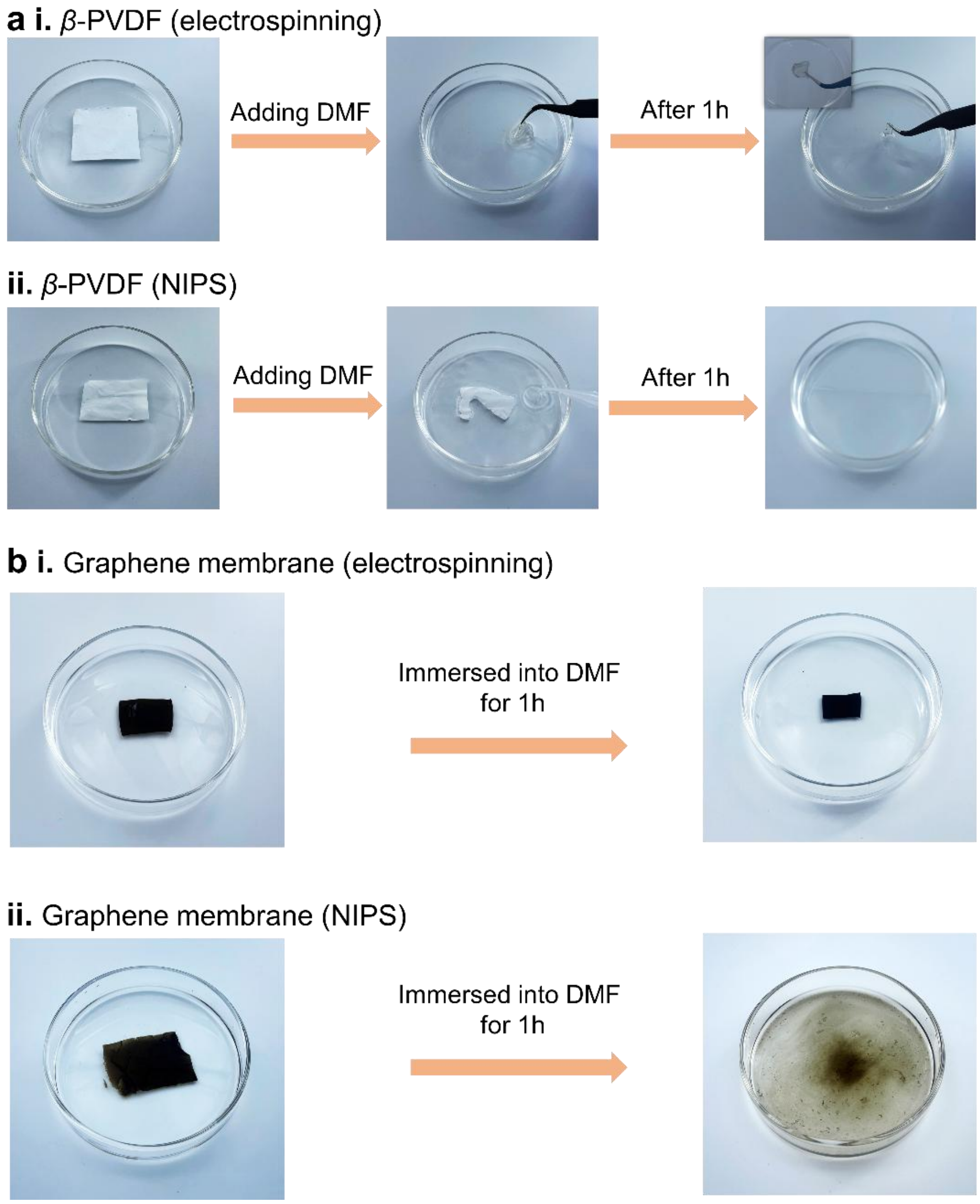

Supplementary Figure 29. (a) Digital photographs of PVDF membranes fabricated by (i) electrospinning and (ii) the NIPS method, before and after immersion in DMF for 1 hour. The inset in (i) shows the dried PVDF (electrospun) membrane post-immersion. (b) Digital photographs of (i) graphene membrane prepared by electrospinning and (ii) graphene-β-PVDF membranes prepared by NIPS, before and after immersion in DMF for 1 hour.

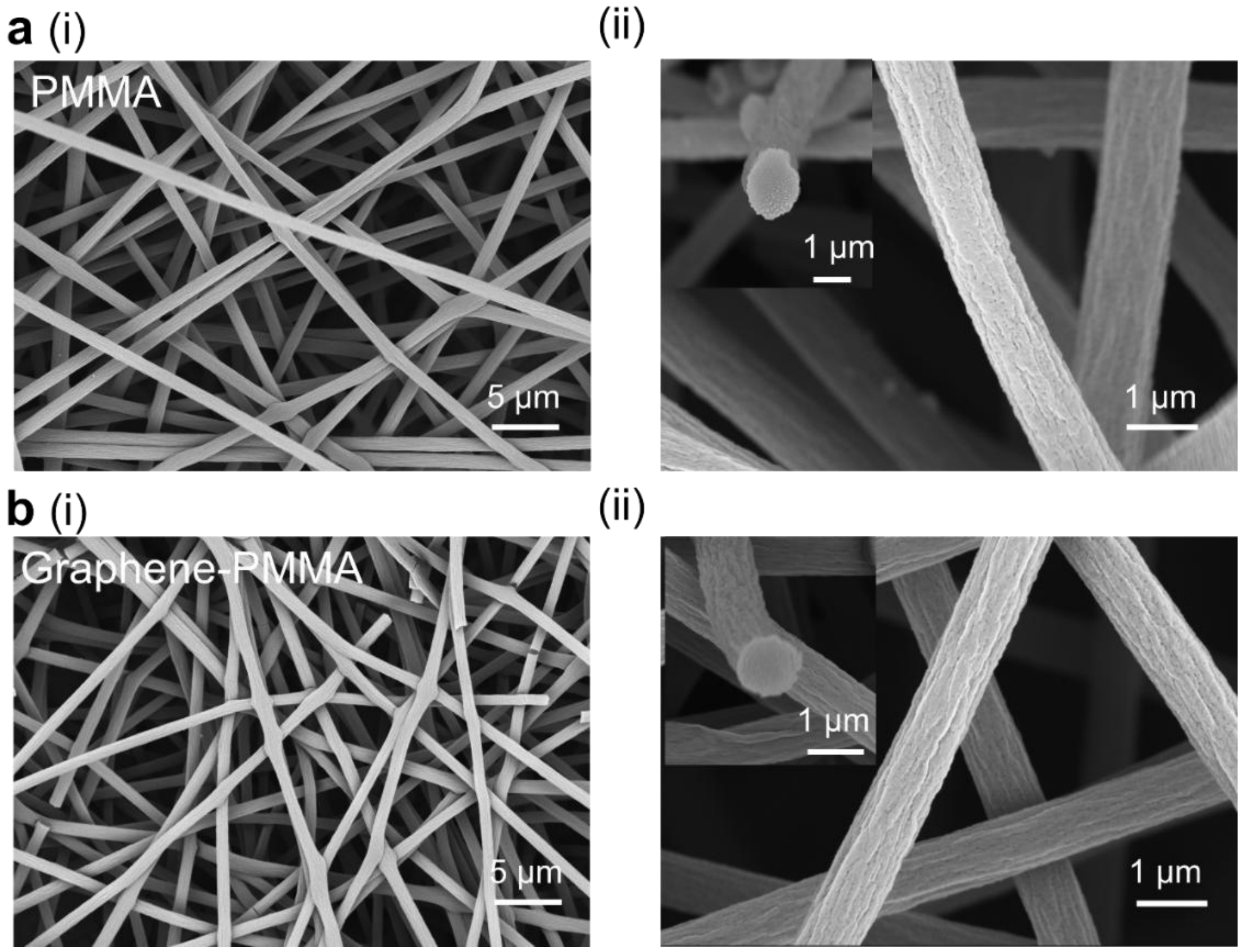

Supplementary Figure 30. SEM images of (a, i) PMMA and (b, i) graphene-PMMA membrane. (ii) Corresponding magnified SEM images. In panel (ii), the inset shows the cross-sectional SEM image of the nanofiber.

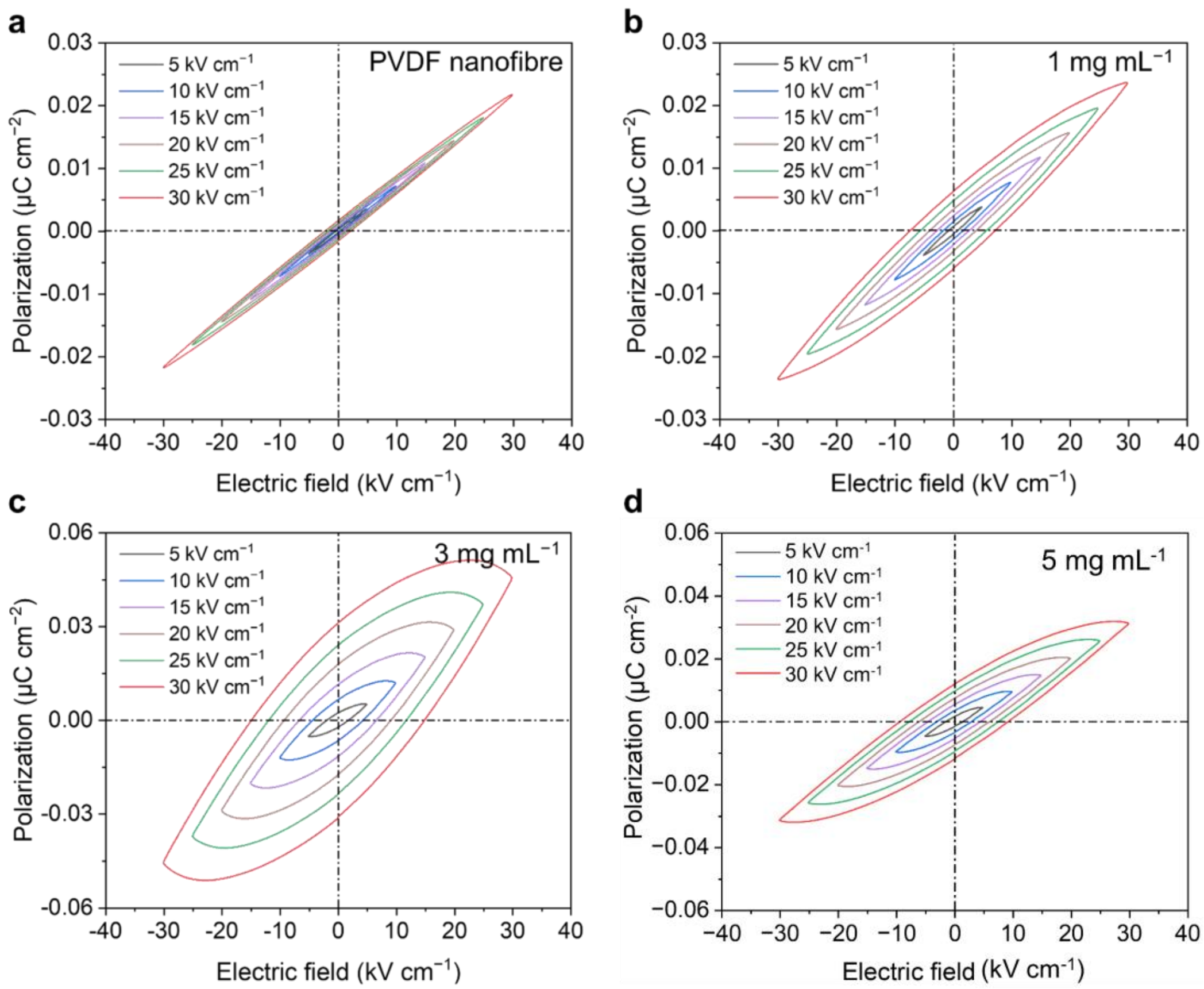

Supplementary Figure 31. Polarization versus electric field (*P*-*E*) hysteresis loops of (a) PVDF and (b-d) GO membranes with different GO concentrations.

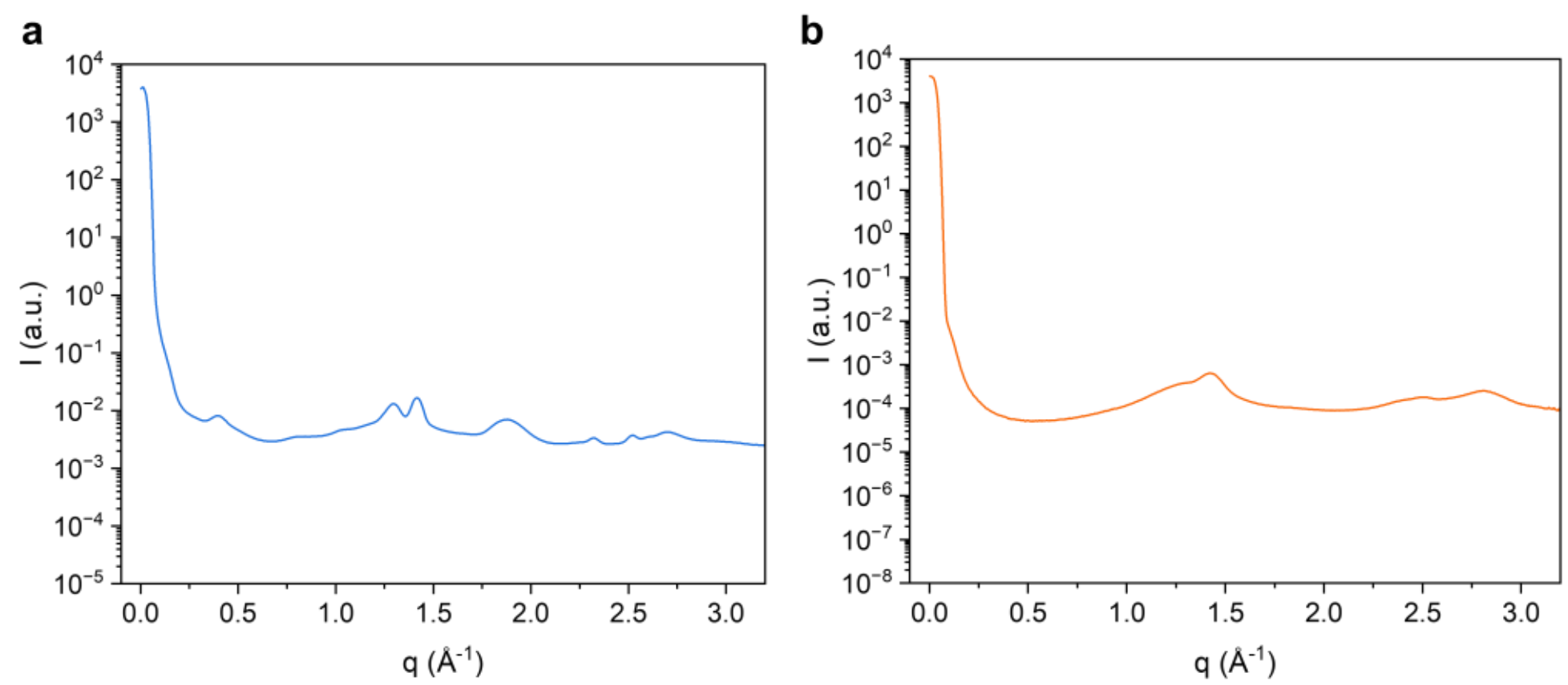

Supplementary Figure 32. WAXs I-q profiles of (a) PVDF powder and (b) GO membrane with a GO concentration of 3 mg mL^{-1}.

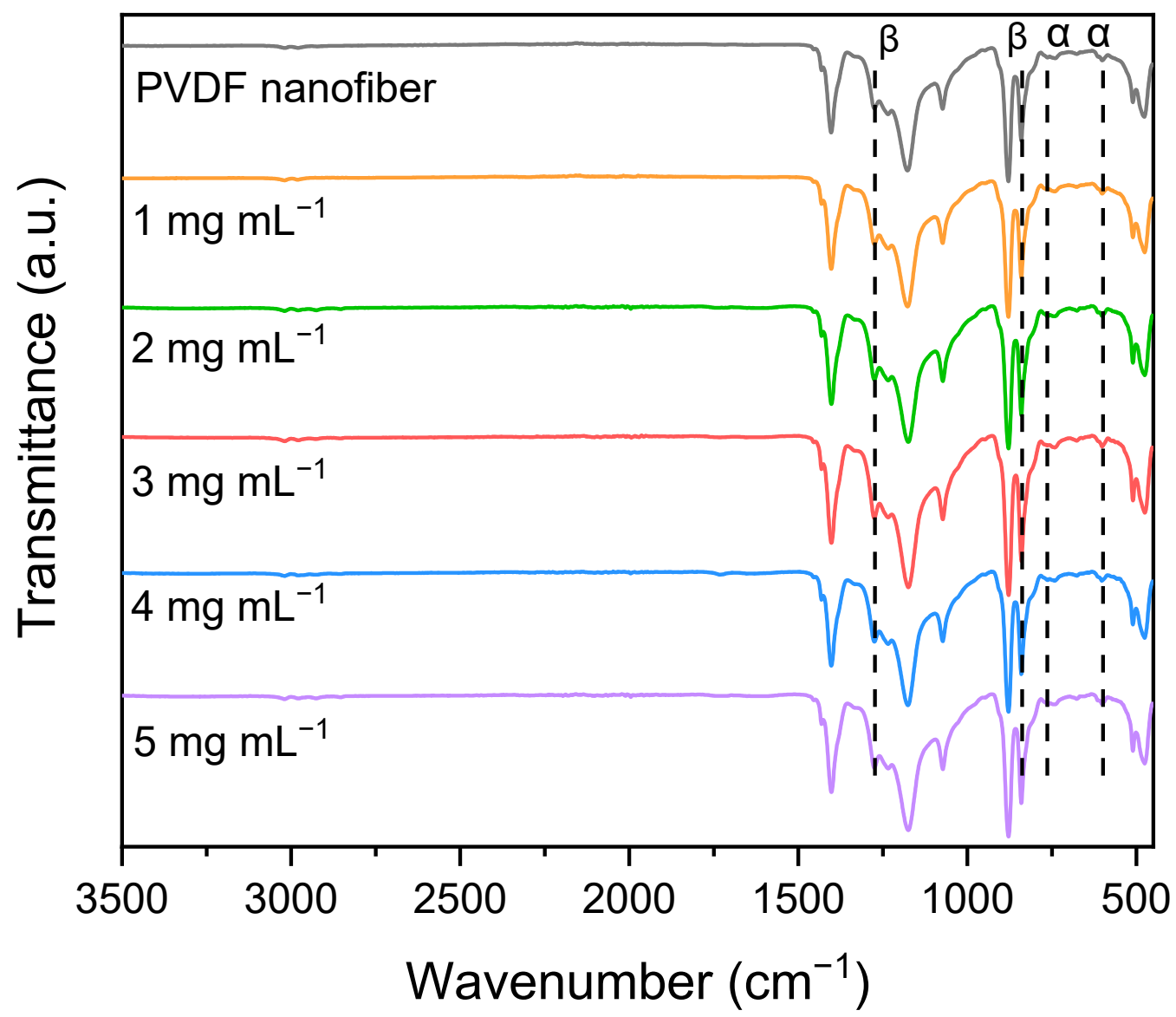

Supplementary Figure 33. FTIR spectra of PVDF and GO membranes prepared at different GO concentrations.

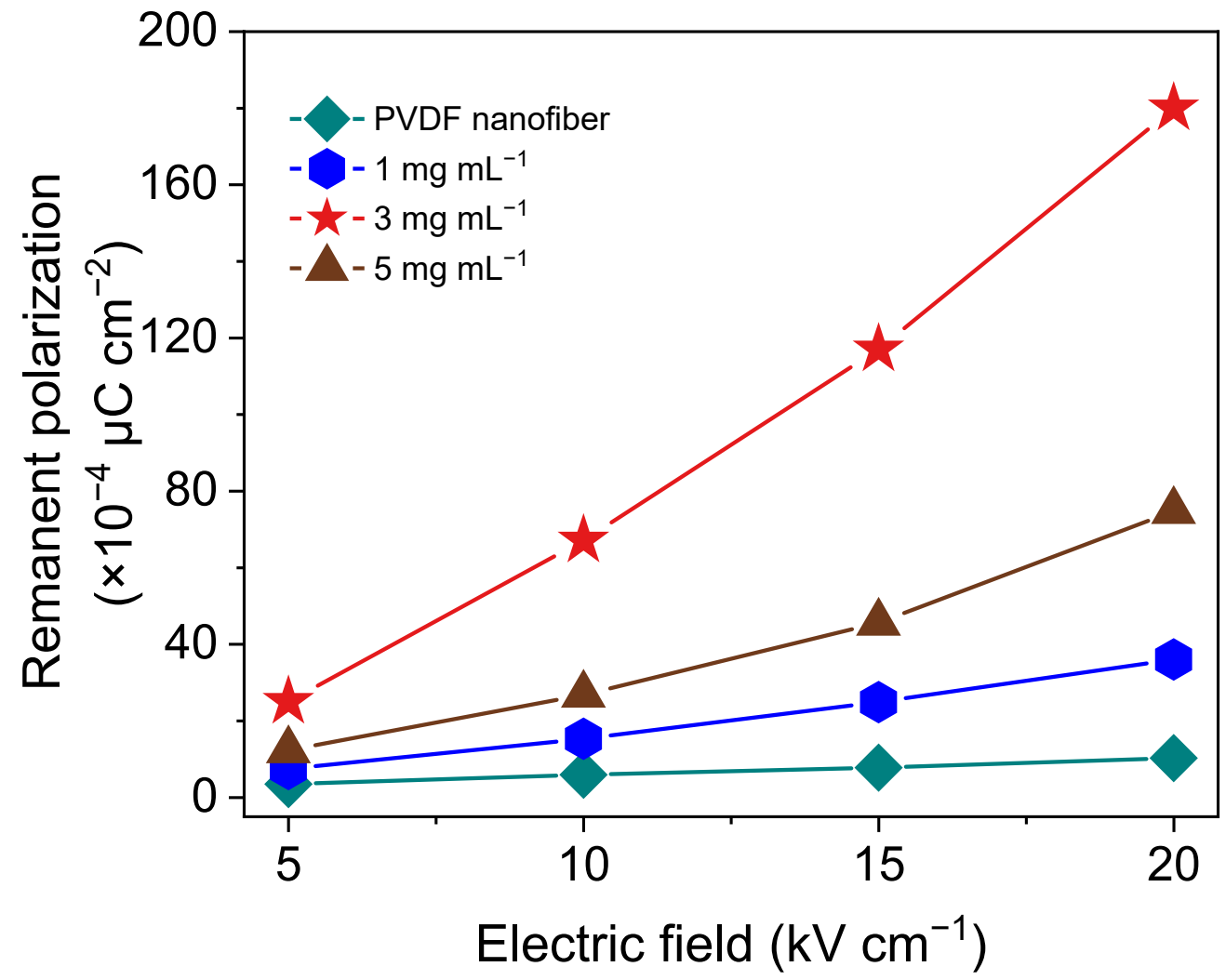

Supplementary Figure 34. Remanent polarization of PVDF and GO membranes prepared at different GO concentrations, which was obtained according to Supplementary Figure 31.

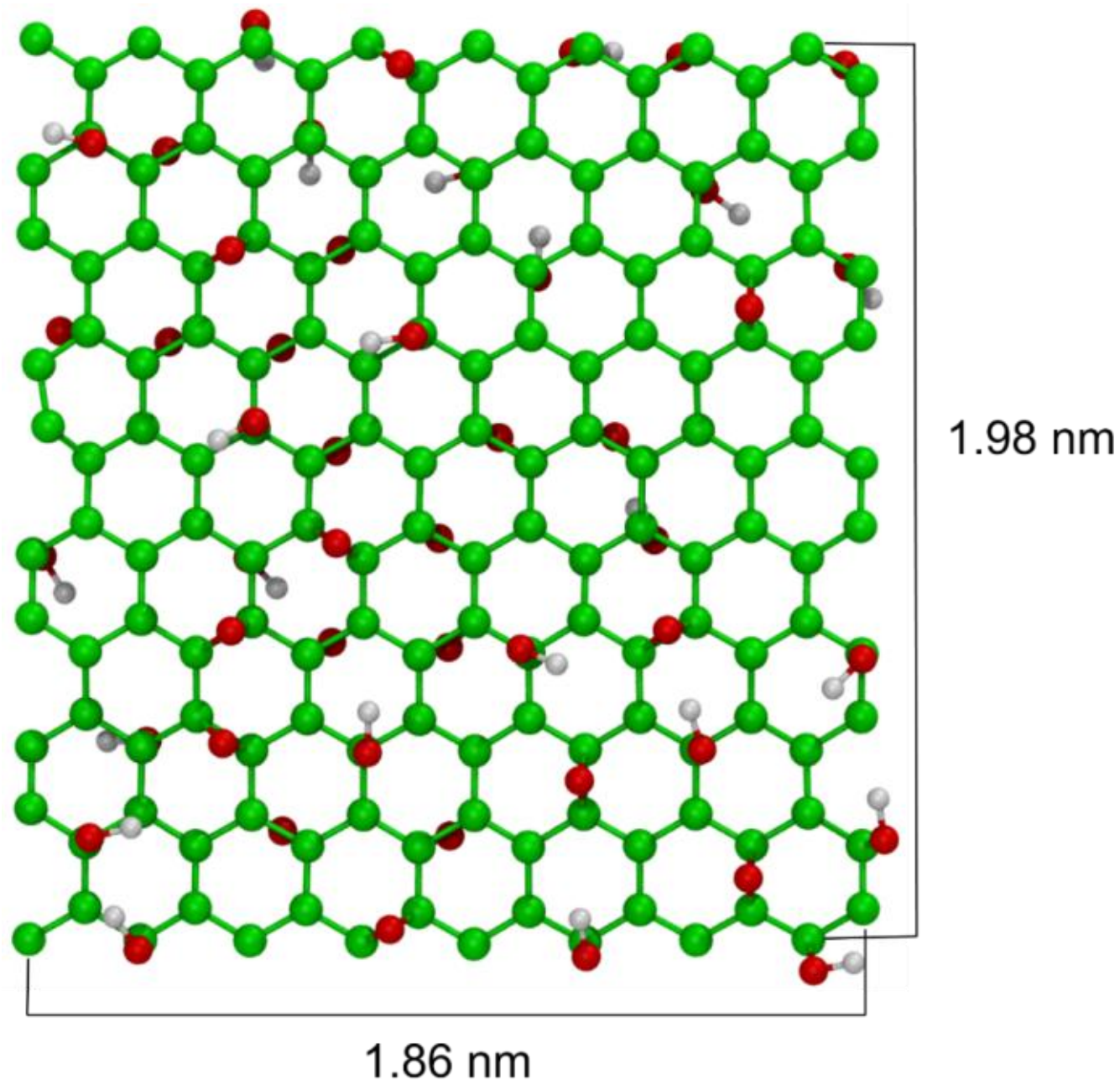

Supplementary Figure 35. Geometry of GO. The carbon, oxygen and hydrogen atoms are represented by green, red and white balls, respectively.

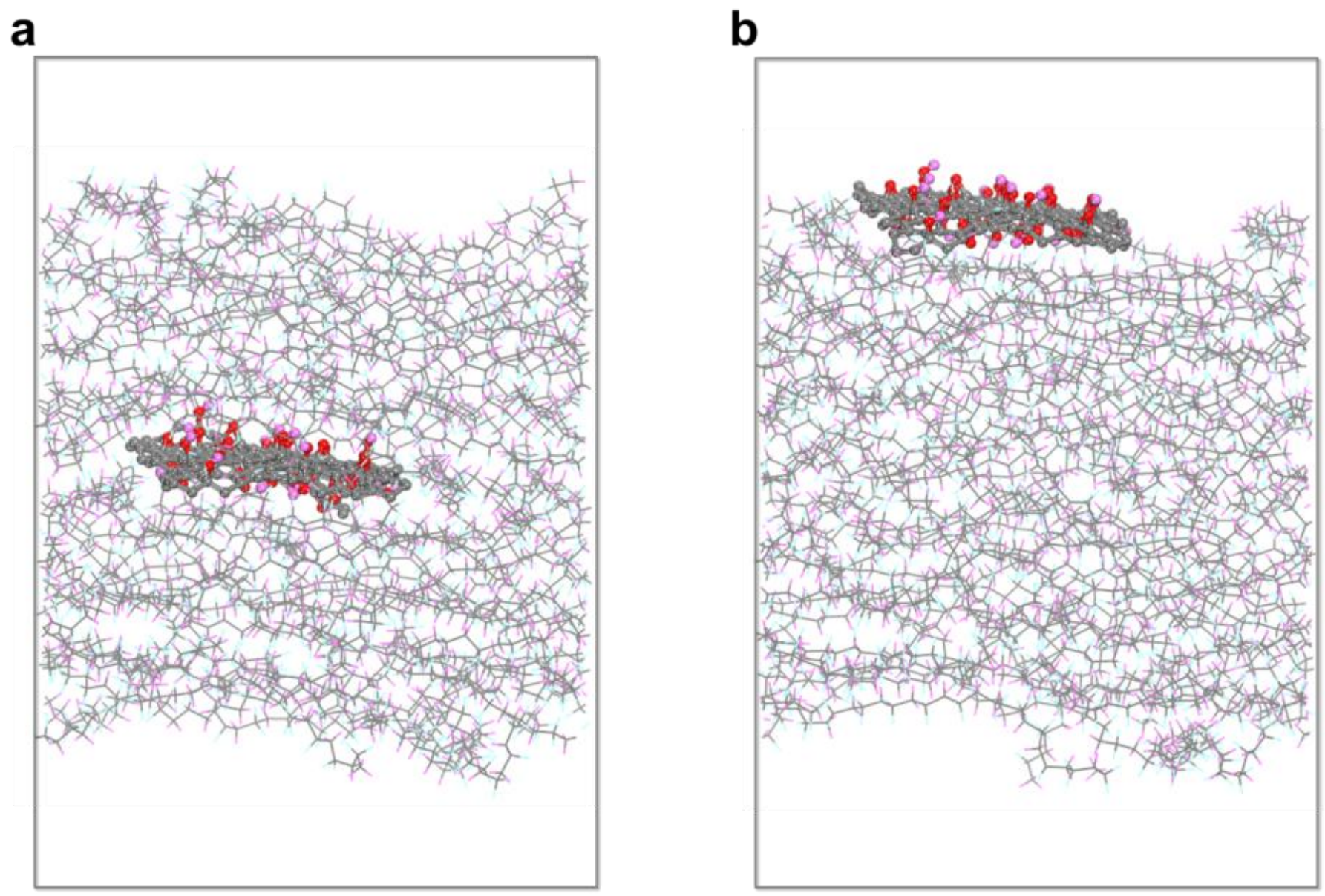

Supplementary Figure 36. Molecular simulations of the energy barrier of GO inside and outside of PVDF chains: Representative configurations of GO inside (a) and outside (b) PVDF used for the energy calculation.

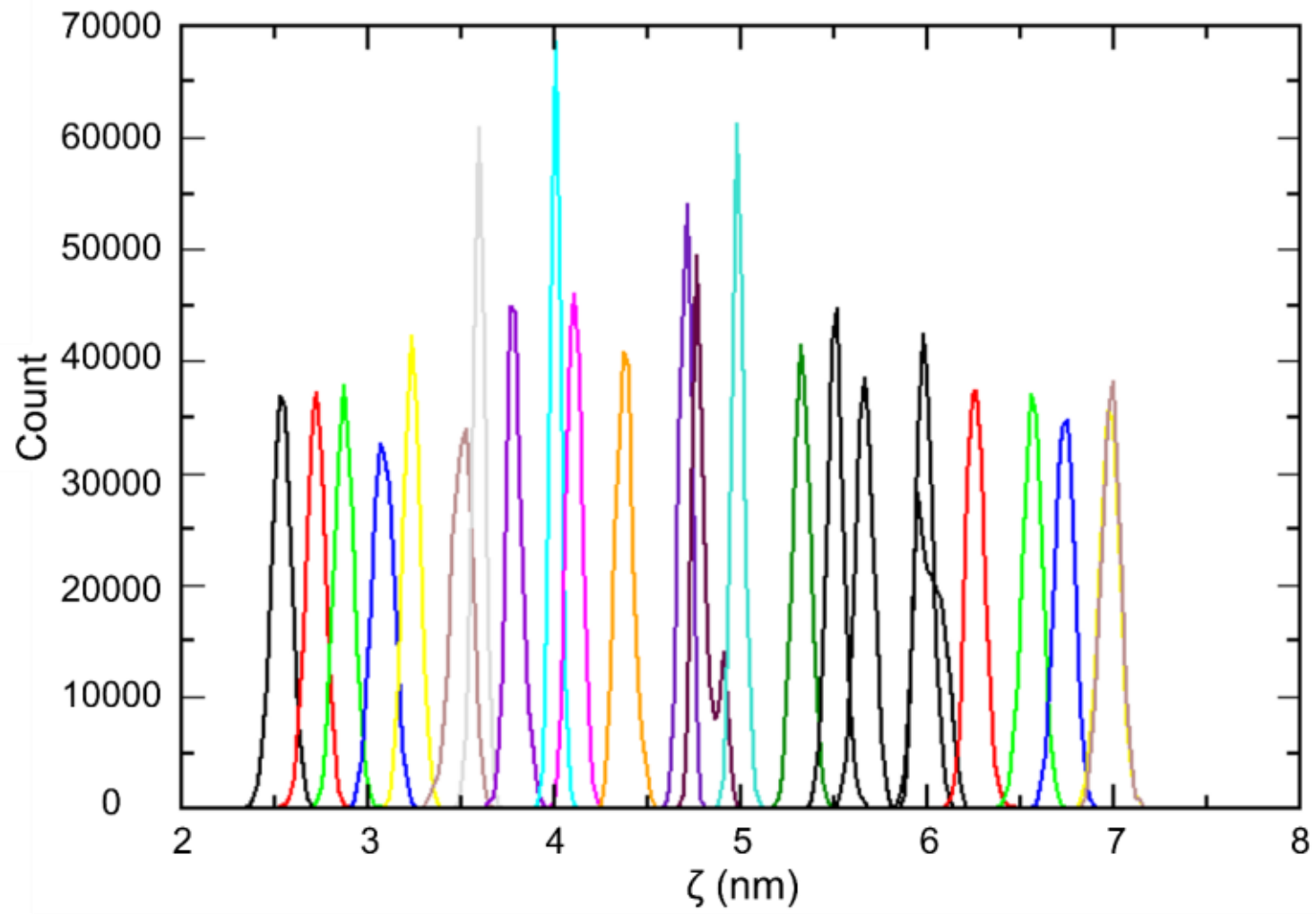

Supplementary Figure 37. Umbrella sampling histograms for GO transfer across PVDF chains. The good overlap of the sampling histograms between each window indicates that sufficient sampling has been obtained.

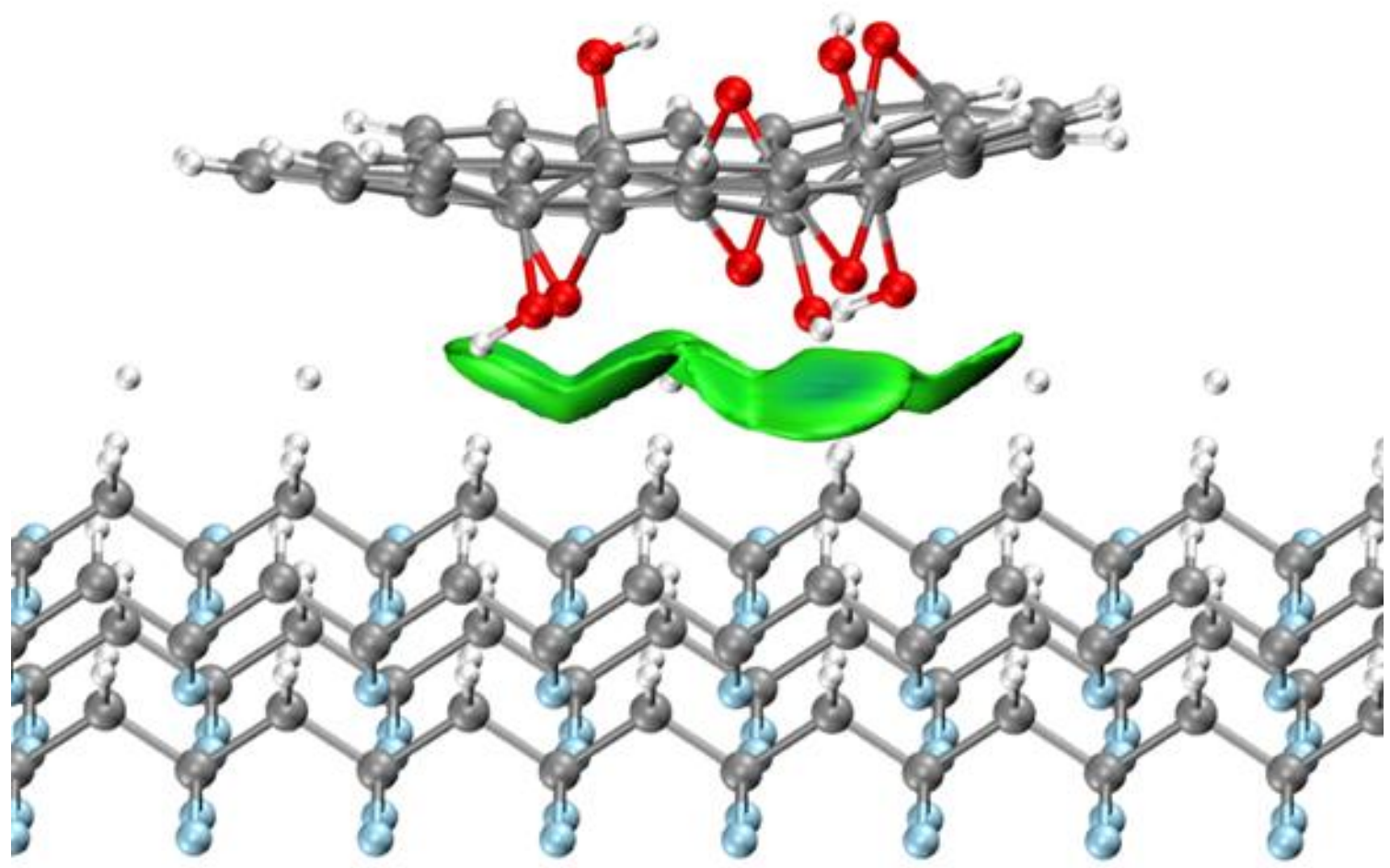

Supplementary Figure 38. The interaction energy calculated between GO and *β*-PVDF (when -CH_2 groups face GO) is −0.056 kcal mol^{-1} $Å^{-2}$ based on the DFT method, which is weaker compared to *α*-PVDF. The IGMH analysis was performed to reveal the interactions. The isovalue of the IGMH isosurface is 0.001 a.u. C atom is in grey, O atom is in red, F atom is in light cyan and H atom is in white. The isosurface was color mapped by sign(λ_2)ρ. ρ is the electron density at the weak interaction critical point, sign(λ_2) is sign of the second largest eigenvalue λ_2 of the electron density Hessian matrix. The region with green color represents mainly the weak attractive van der Waals interaction. The cyan-to-blue region represents the relatively strong attractive interactions, including hydrogen bonds and electrostatic attraction. The brown-to-red region represents the prominent repulsive interactions, including steric hinderance and electrostatic repulsion.

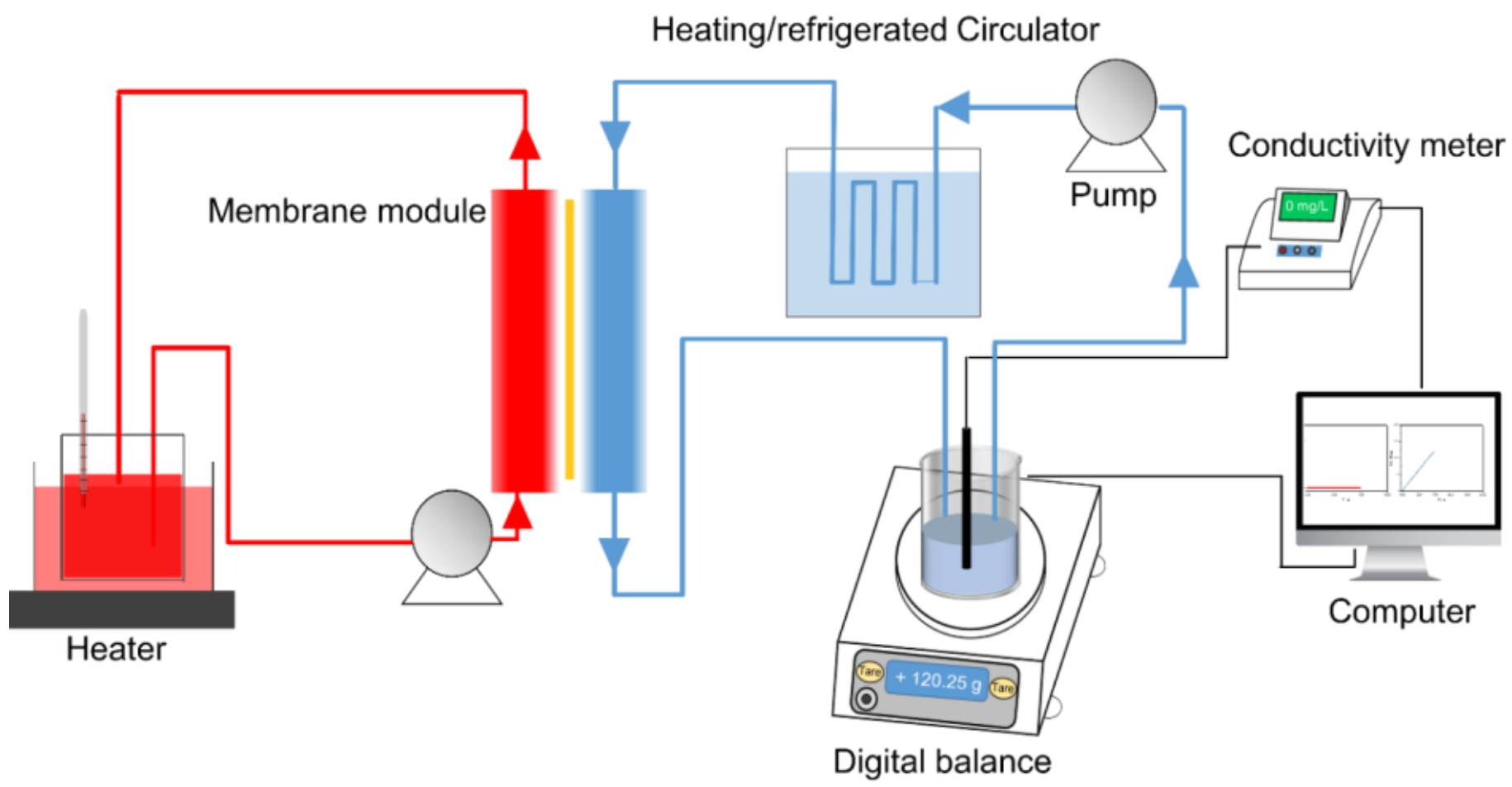

Supplementary Figure 39. Schematic illustration of the hydrophobic interfacial evaporation desalination setup.

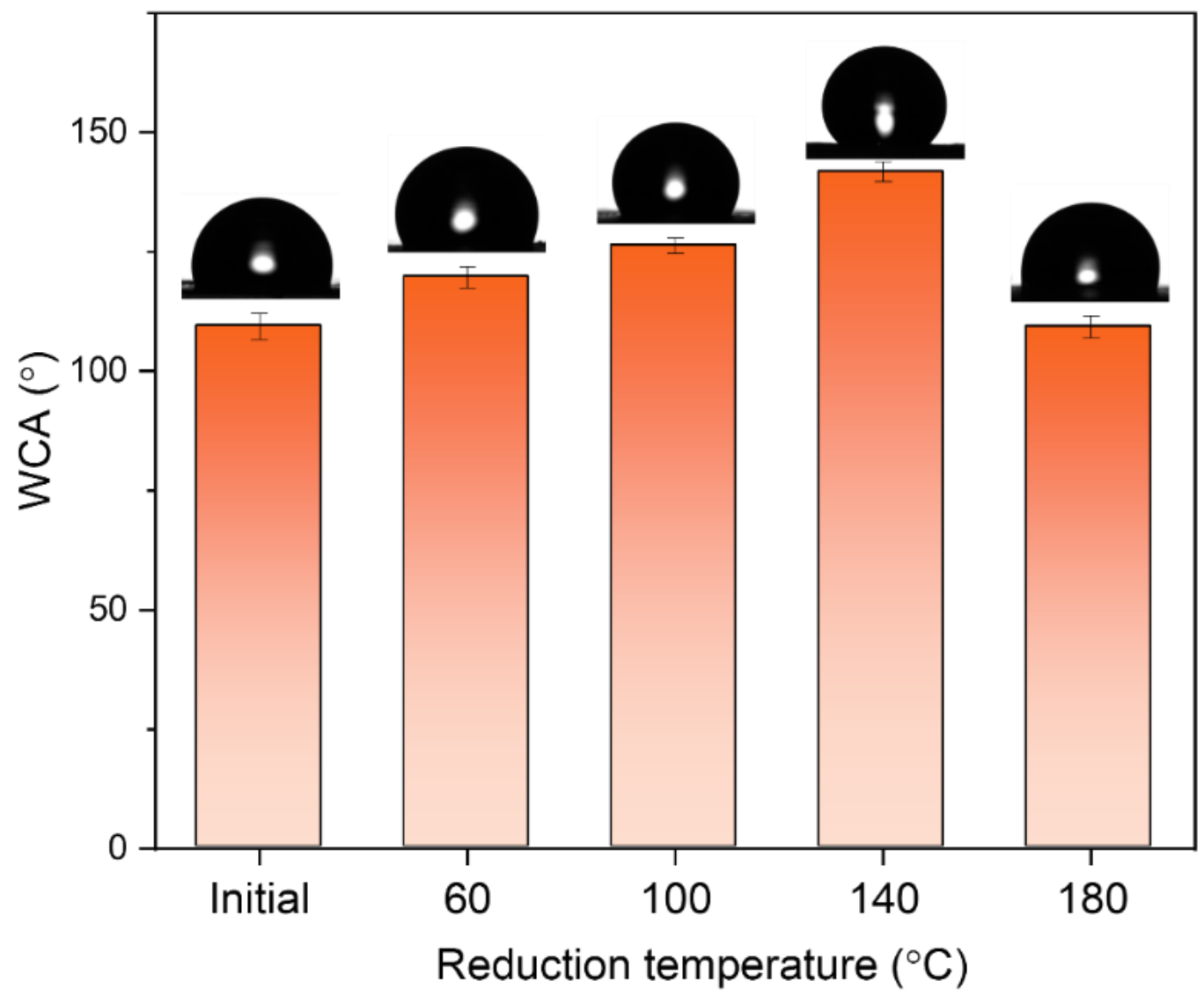

Supplementary Figure 40. Water contact angle (WCA) of graphene membranes treated at different temperatures.

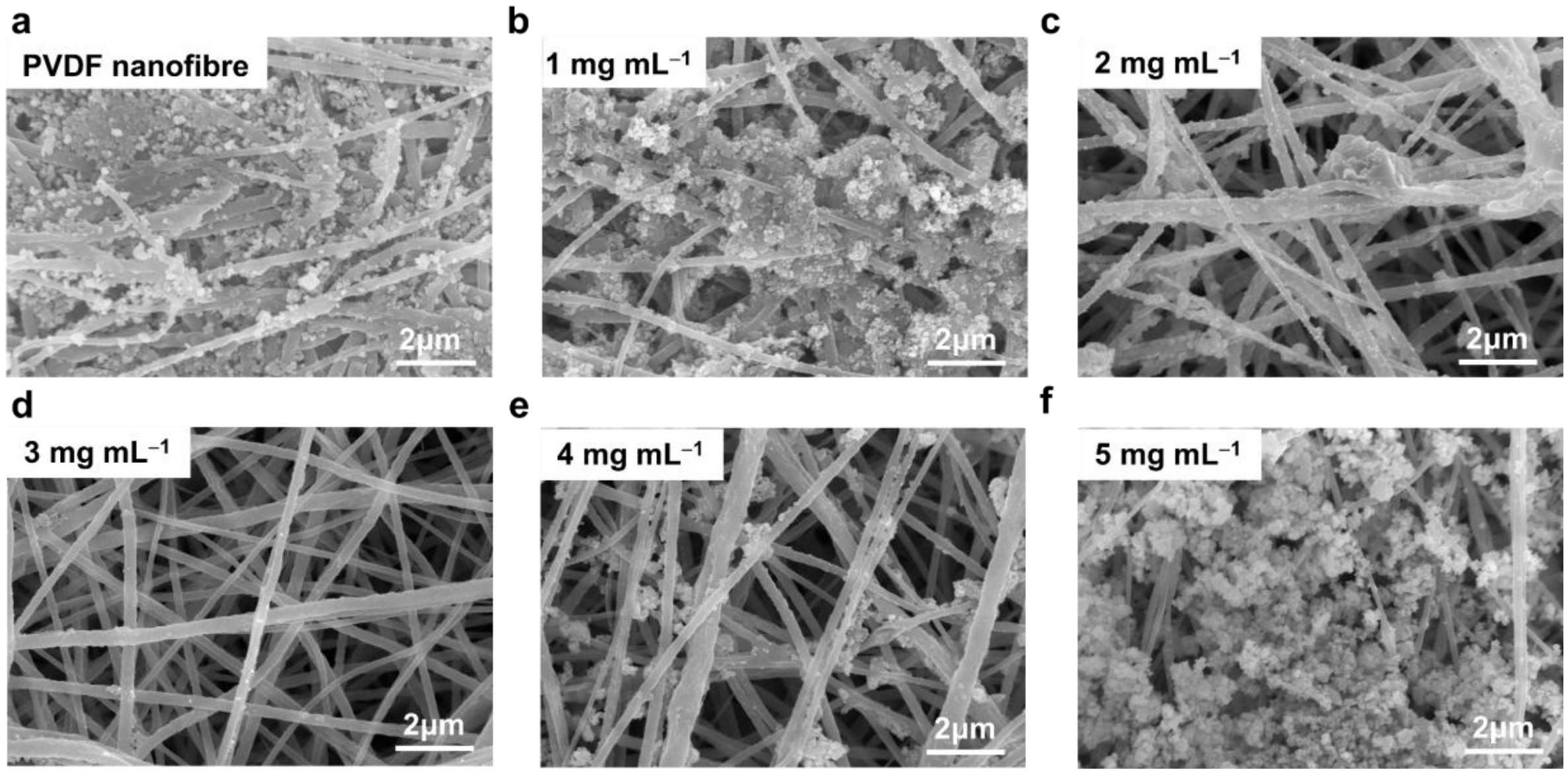

Supplementary Figure 41. SEM surface images of PVDF nanofiber membrane and graphene membranes fabricated by different nanosheets concentrations after desalination testing. The feed and permeate temperatures are 60°C and 20°C, respectively. The feed concentration is 150 g L^{-1}.

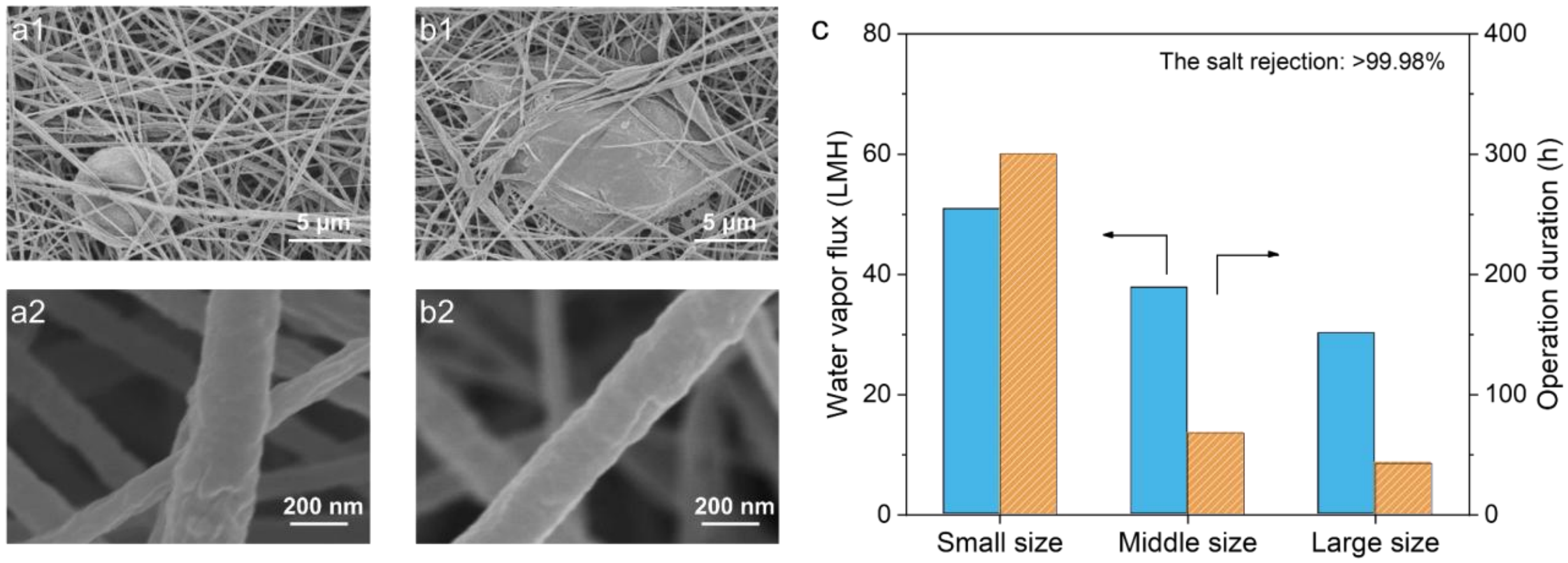

Supplementary Figure 42. SEM images of graphene membranes fabricated using (a1, a2) middle-sized (5–8 μm) and (b1, b2) large-sized (20–30 μm) nanosheets. (a1) and (b1) are the corresponding enlarged SEM images. (c) Water vapor flux and operation duration when the salts rejection is higher than 99.99% of graphene membranes generated from GO nanosheets with different lateral size, the feed temperature is 60°C and the feed concentration is 150 g L^{-1}.

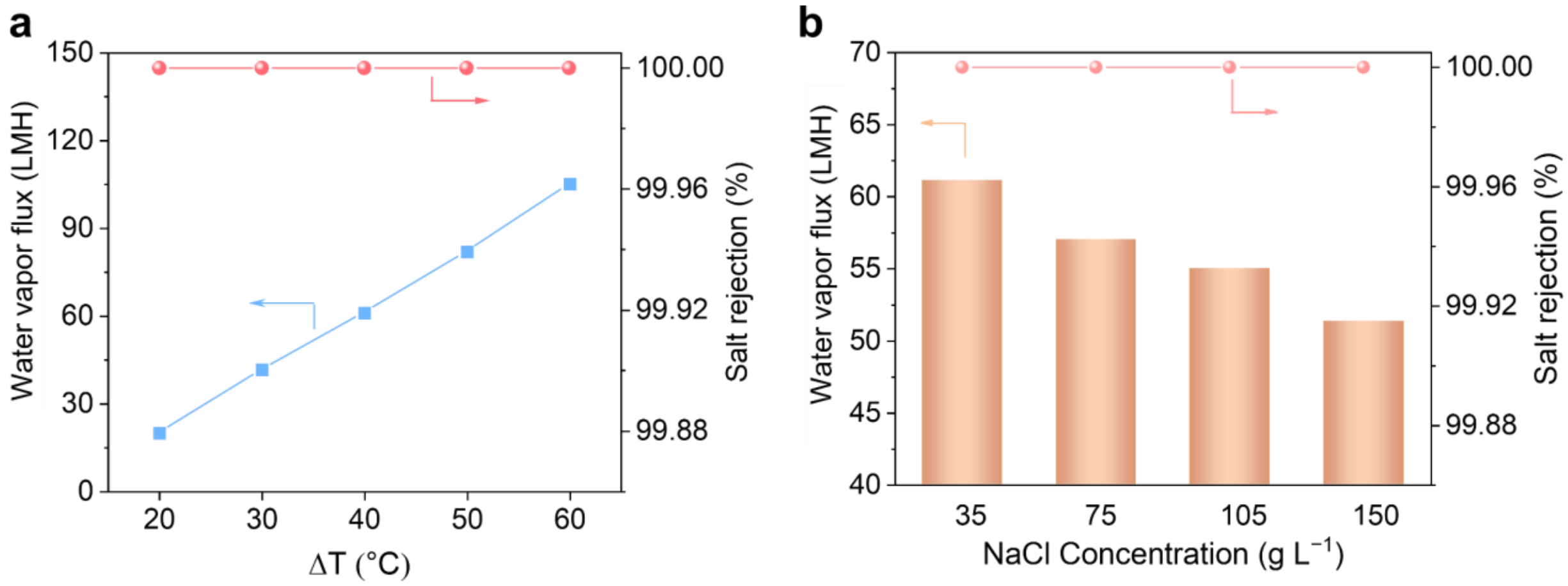

Supplementary Figure 43. Desalination performance of the graphene membrane. (a) Effect of operation temperature, with a fixed permeate temperature of 20°C and a feed NaCl concentration of 35 g L^{-1}. (b) Effect of feed NaCl concentration, with feed and permeate temperatures of 60°C and 20°C, respectively.

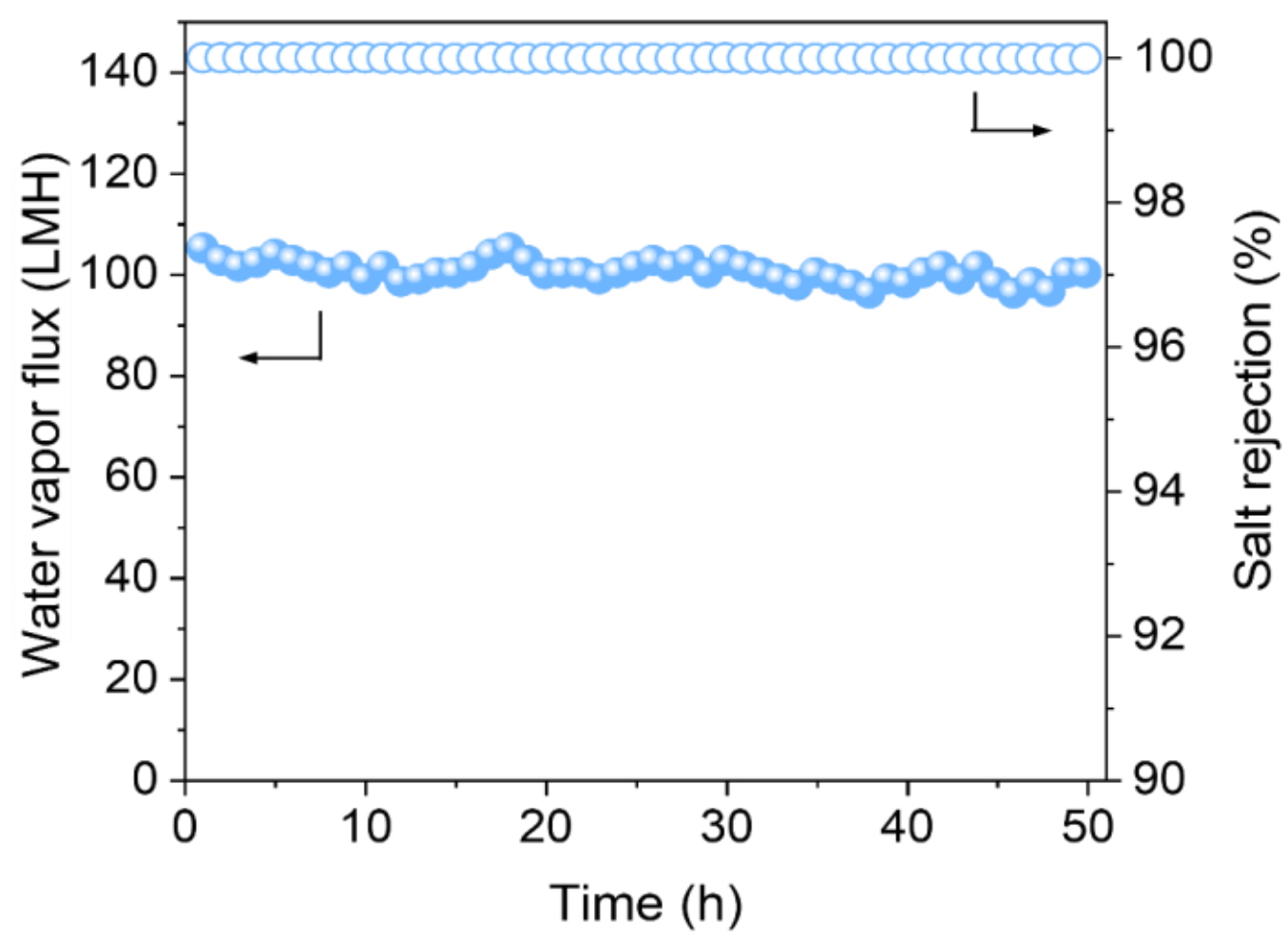

Supplementary Figure 44. Desalination performance of the graphene membrane, tested under feed and permeate temperatures of 80°C and 20°C, respectively, and with a feed NaCl concentration of 35 g L^{-1}.

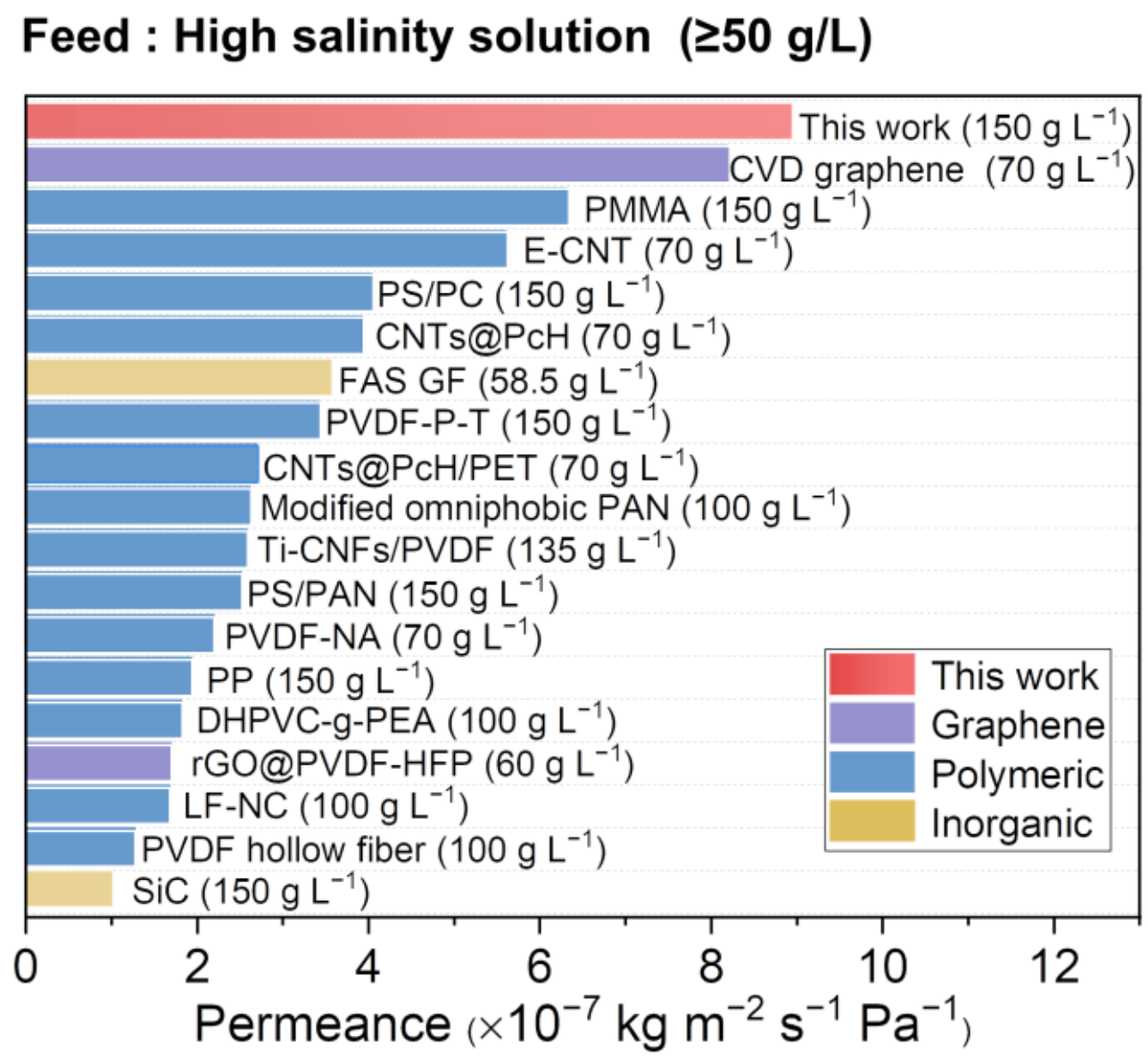

Supplementary Figure 45. Comparison of water permeance with state-of-the-art hydrophobic interfacial evaporation desalination membranes used for high-salinity water desalination.

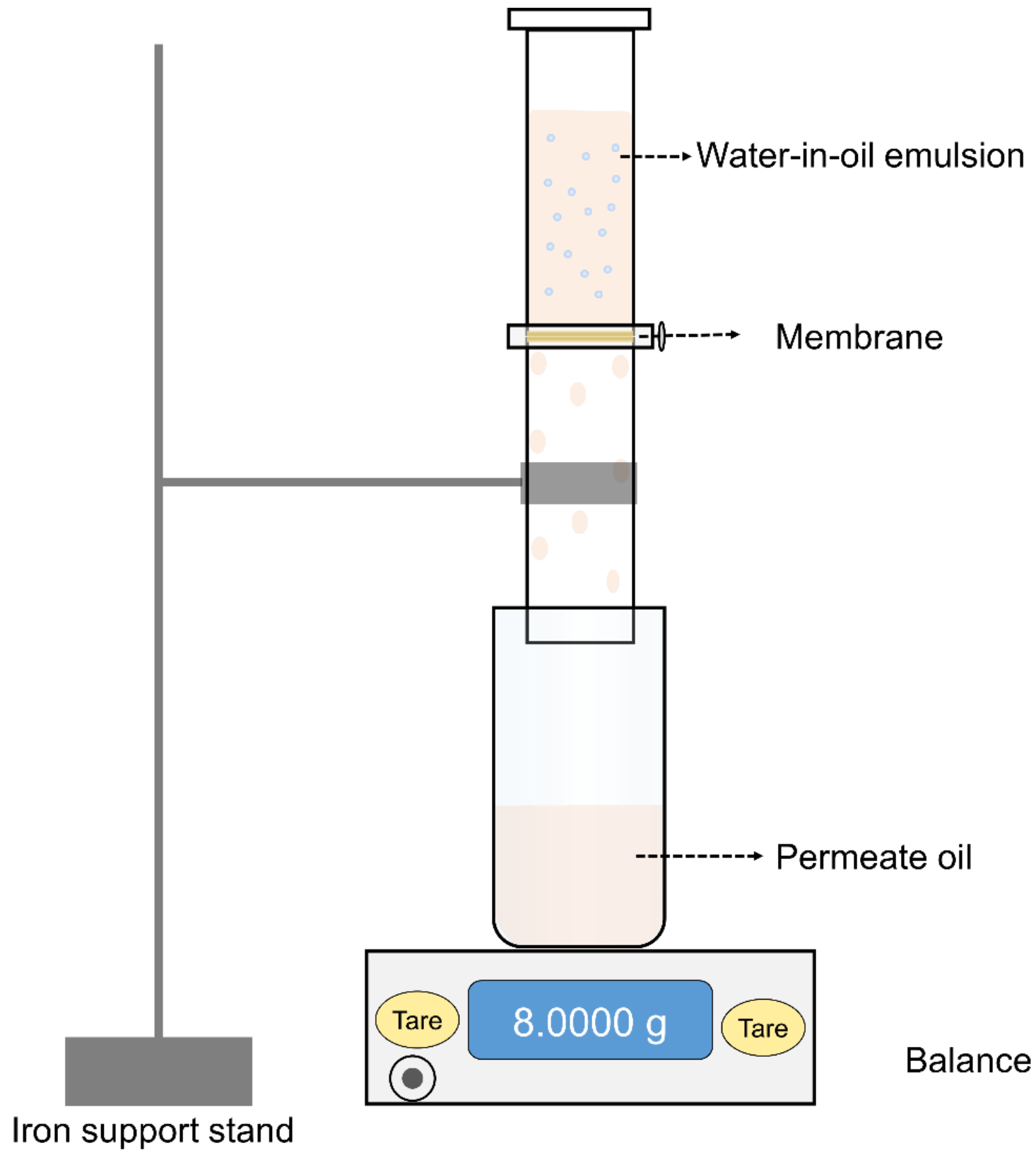

Supplementary Figure 46. Schematic illustration of the oil-water separation setup.

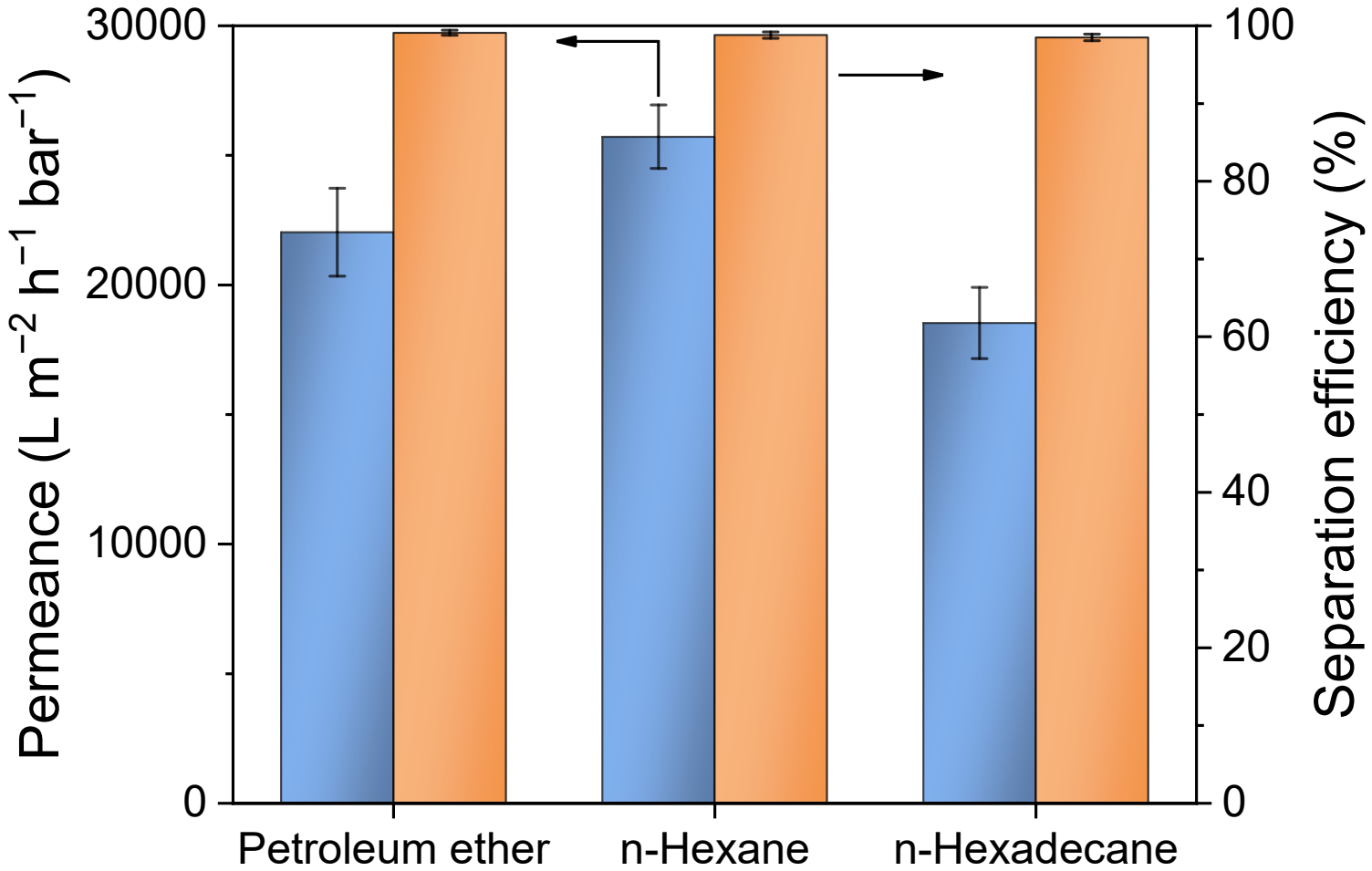

Supplementary Figure 47. Oil/water separation performance of the graphene membrane for water-in-oil emulsions based on petroleum ether, *n*-hexane, and *n*-hexadecane.

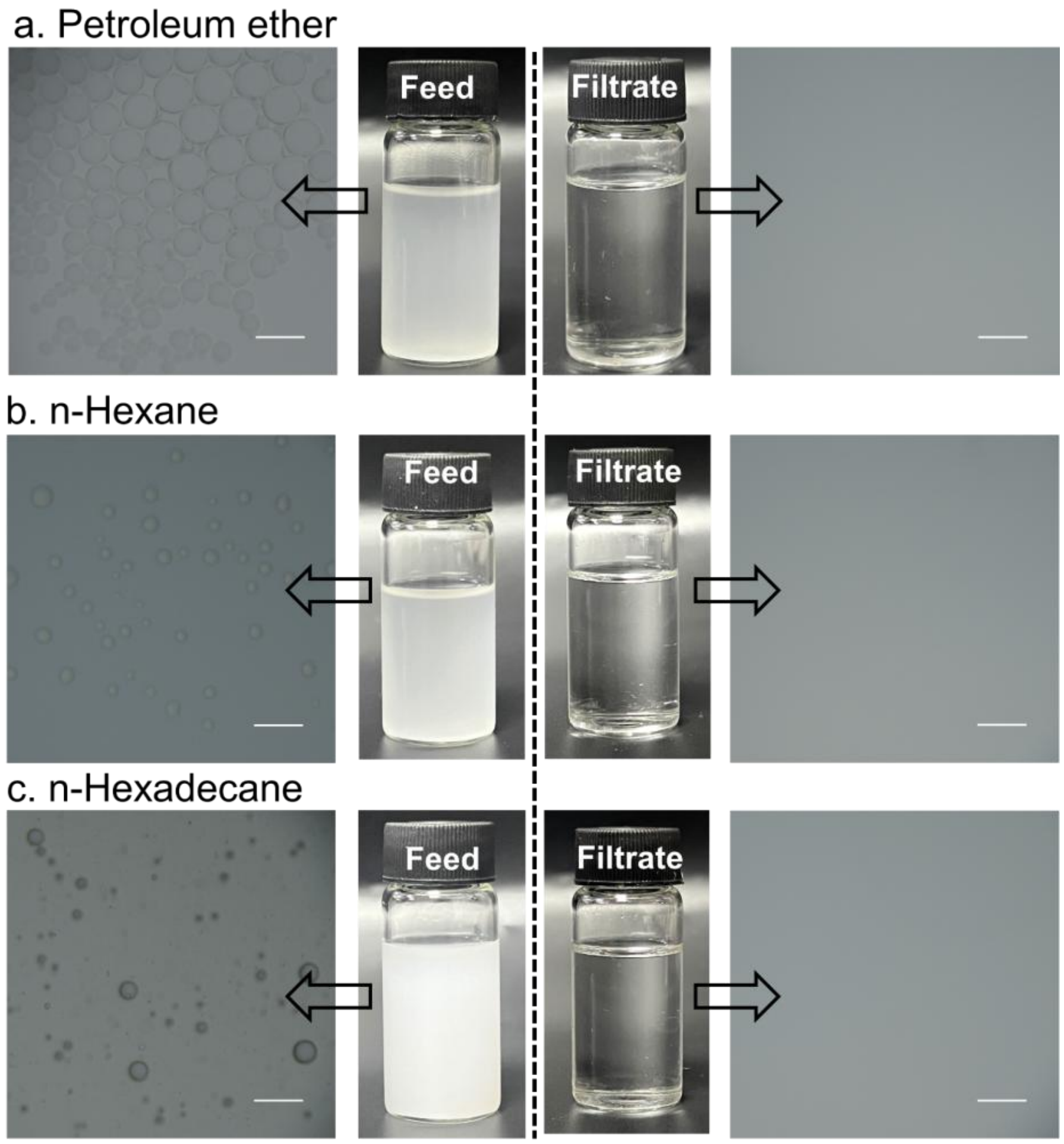

Supplementary Figure 48. Digital photographs and microscopy images of (a) water-in-petroleum ether, (b) water-in-*n*-hexane, and (c) water-in-*n*-hexadecane emulsions before and after filtration. The scale bar represents 50 μm.

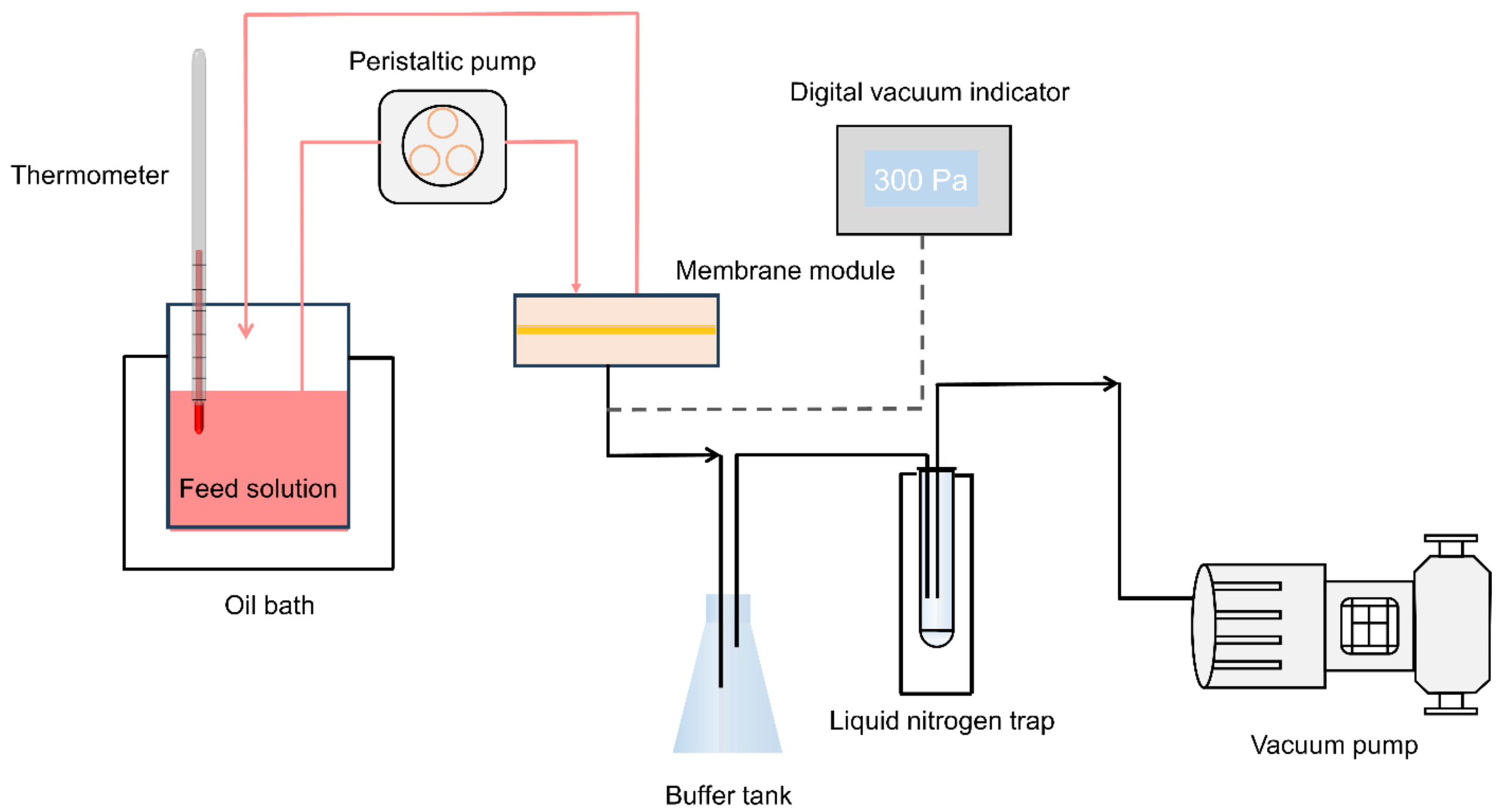

Supplementary Figure 49. Schematic illustration of home-made pervaporation setup.

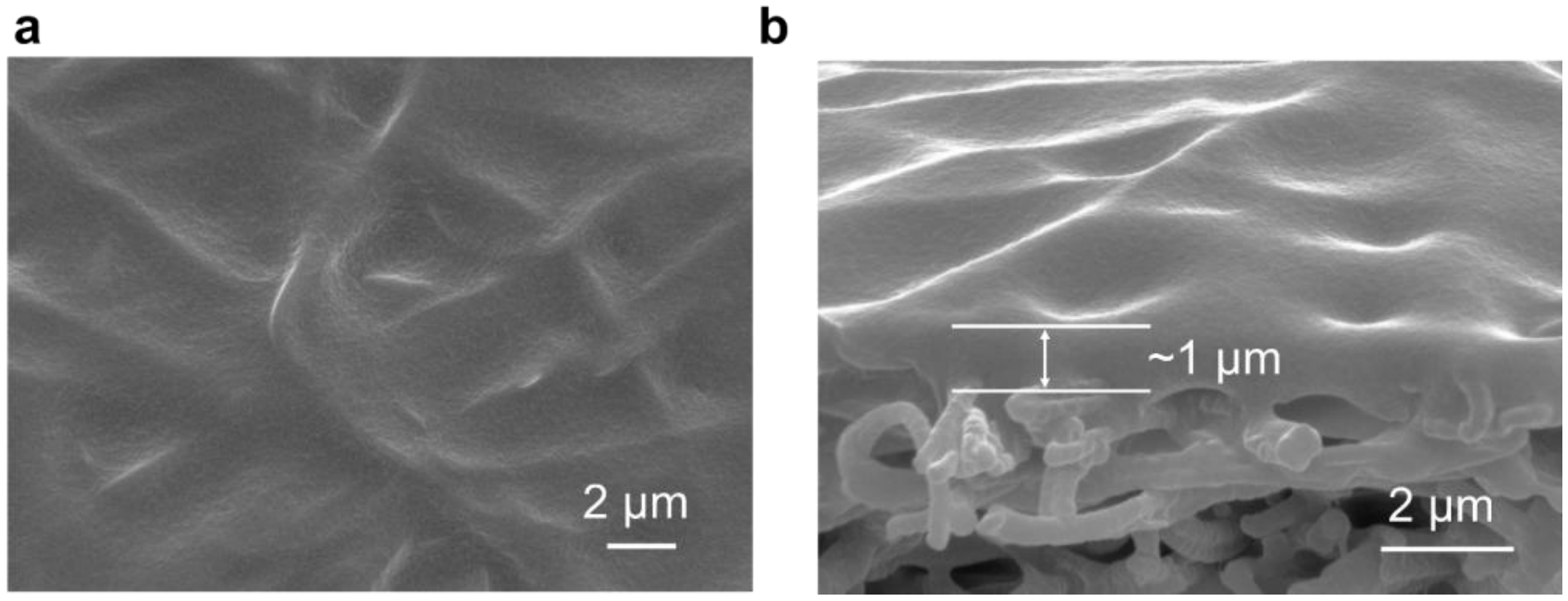

Supplementary Figure 50. SEM surface (a) and cross-sectional (b) images of PDMS@graphene membrane.

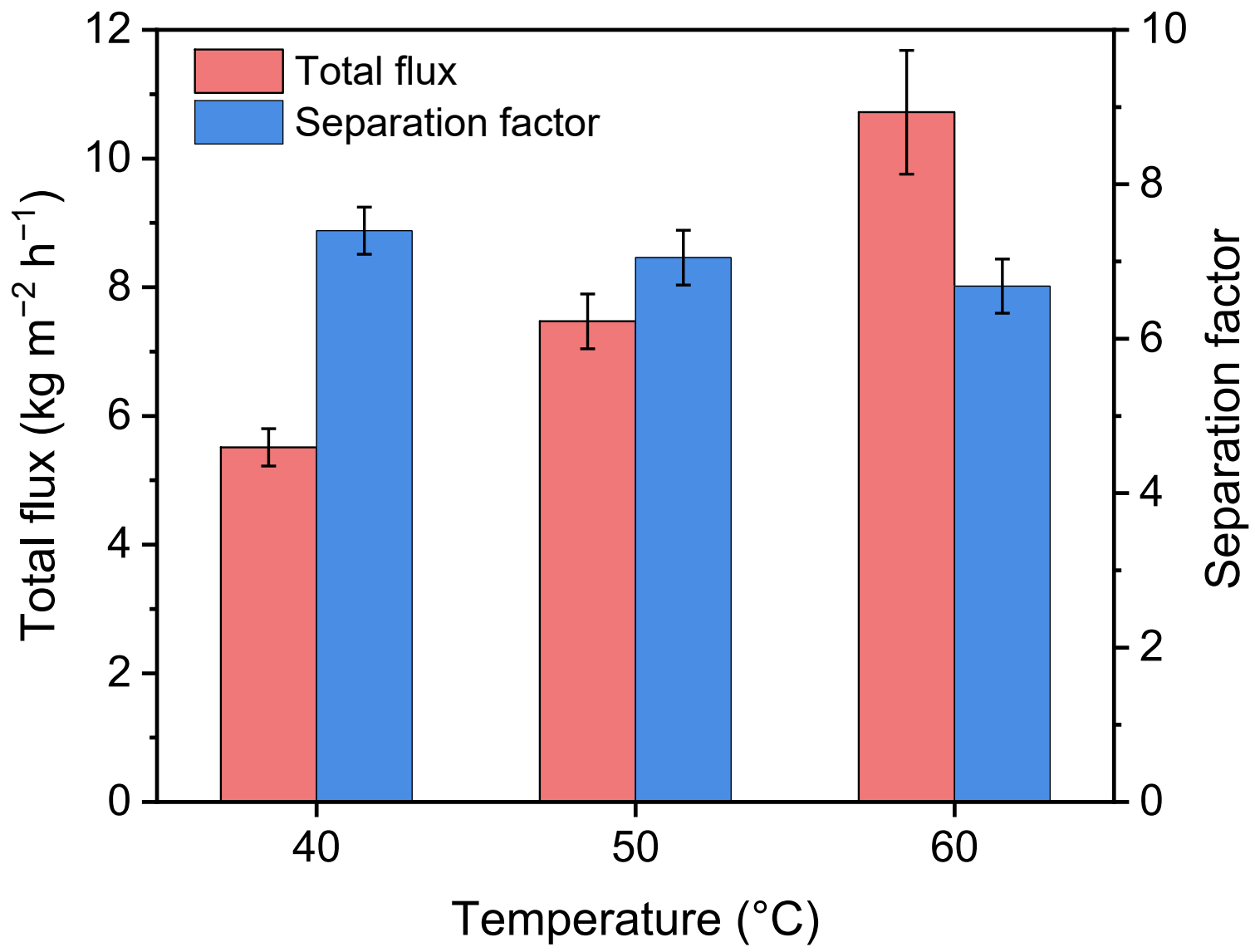

Supplementary Figure 51. The effect of operating temperature on the ethanol/water separation performance of graphene supported PDMS membrane. The feed solution was ethanol/water mixture with an ethanol concentration of 5 wt%.

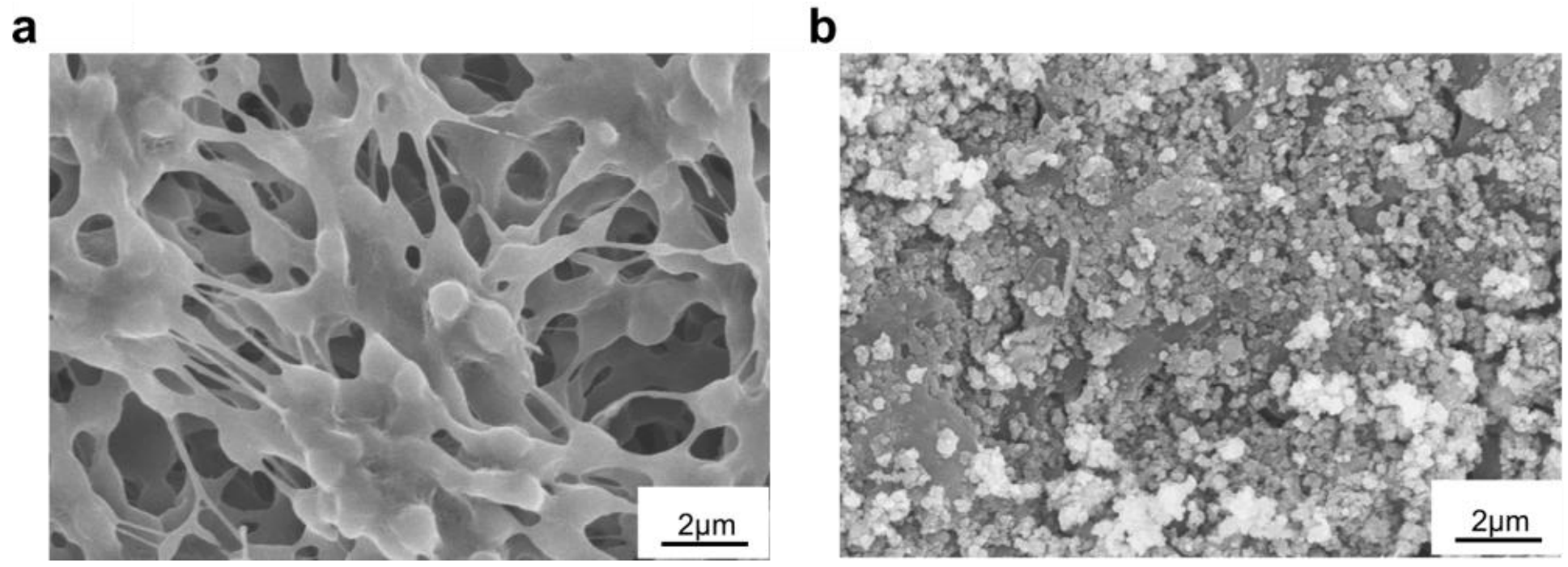

Supplementary Figure 52. SEM images of commercial PVDF (Millipore Durapore, HVHP04700) membrane (a) before and (b) after desalination testing.

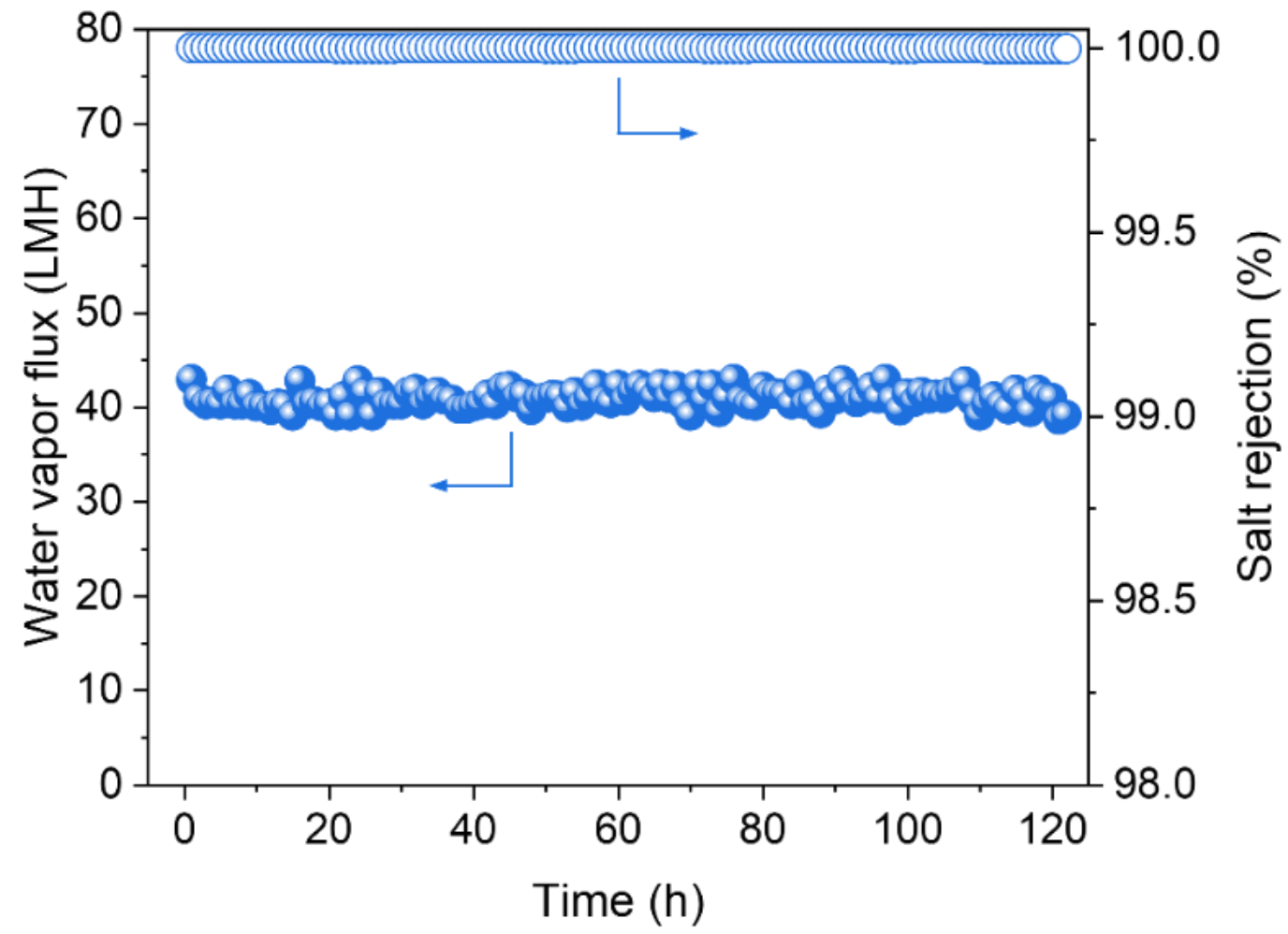

Supplementary Figure 53. Water vapor flux and salt rejection of the FAS-PVDF membrane. The feed and permeate temperatures were 60°C and 20°C, respectively, with a feed NaCl concentration of 35 g L^{-1}.

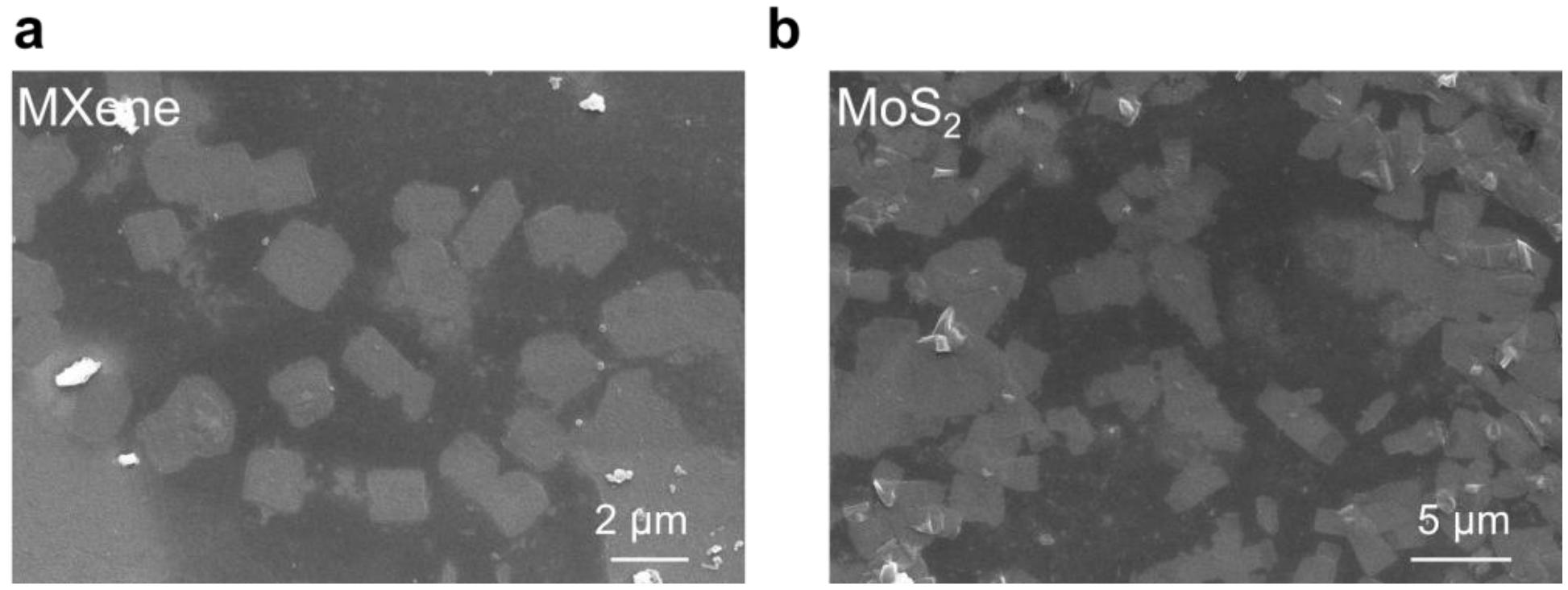

Supplementary Figure 54. SEM images of (a) MXene and (b) MoS_2 nanosheets.

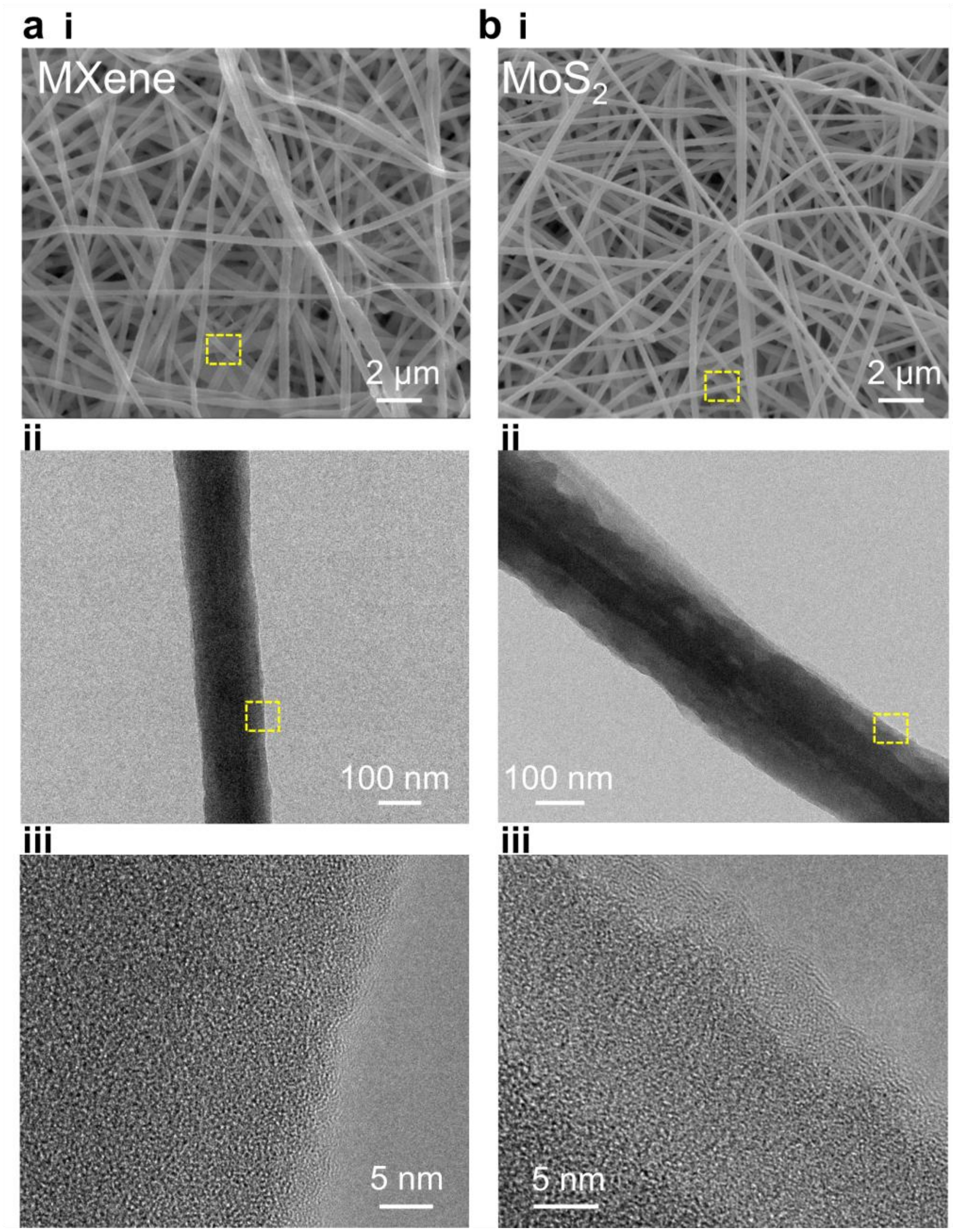

Supplementary Figure 55. SEM images of (a, i) MXene and (b, i) MoS_2 membranes fabricated via the ferroelectric-polarization-assisted strategy. Panels (ii) and (iii) show the corresponding TEM images. The dashed boxes in (i) and (ii) indicate the areas selected for (ii) and (iii), respectively.

a

b

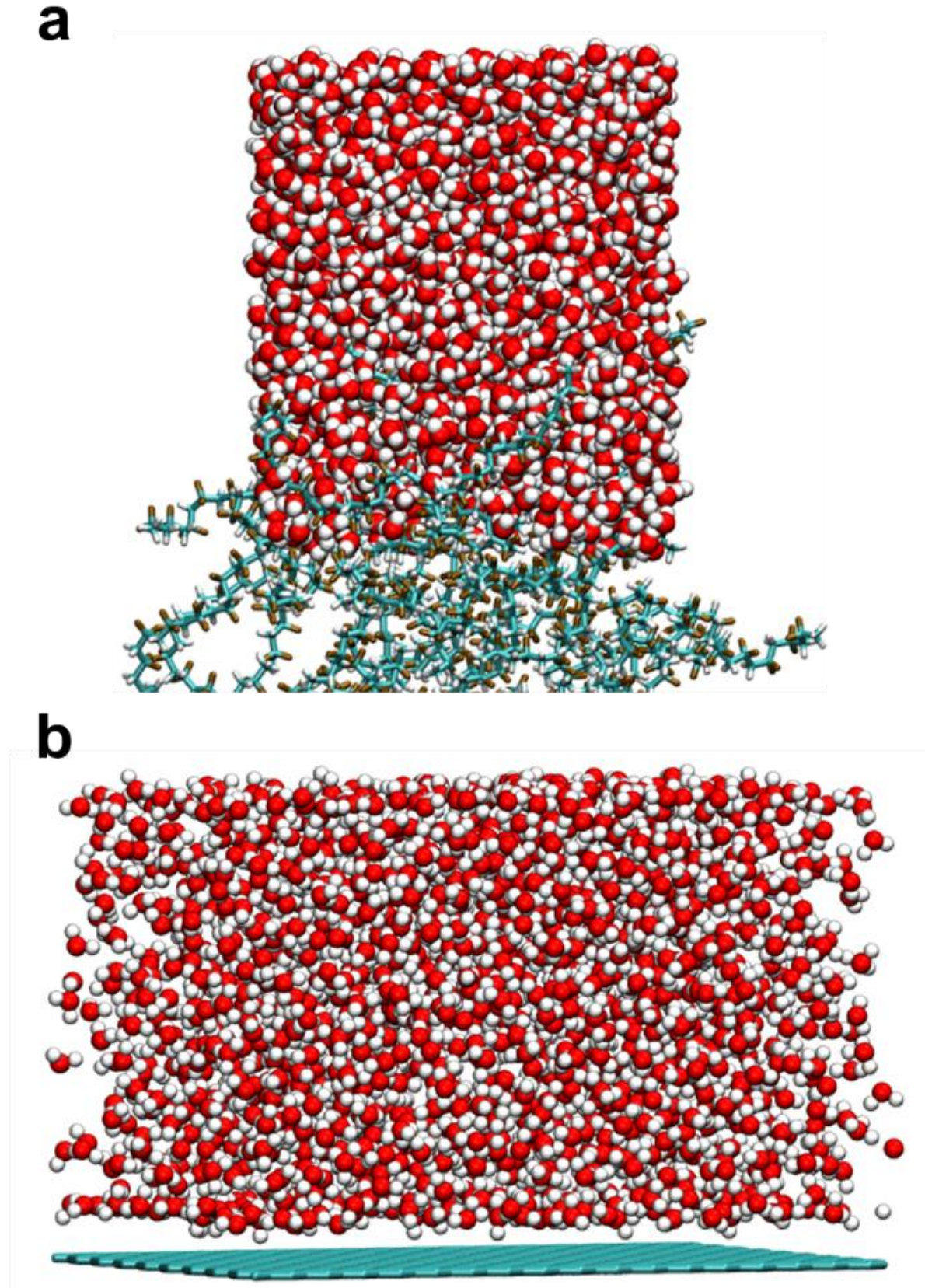

Supplementary Figure 56. Representative snapshots showing water sliding on (a) PVDF and (b) graphene slabs at the initial position.

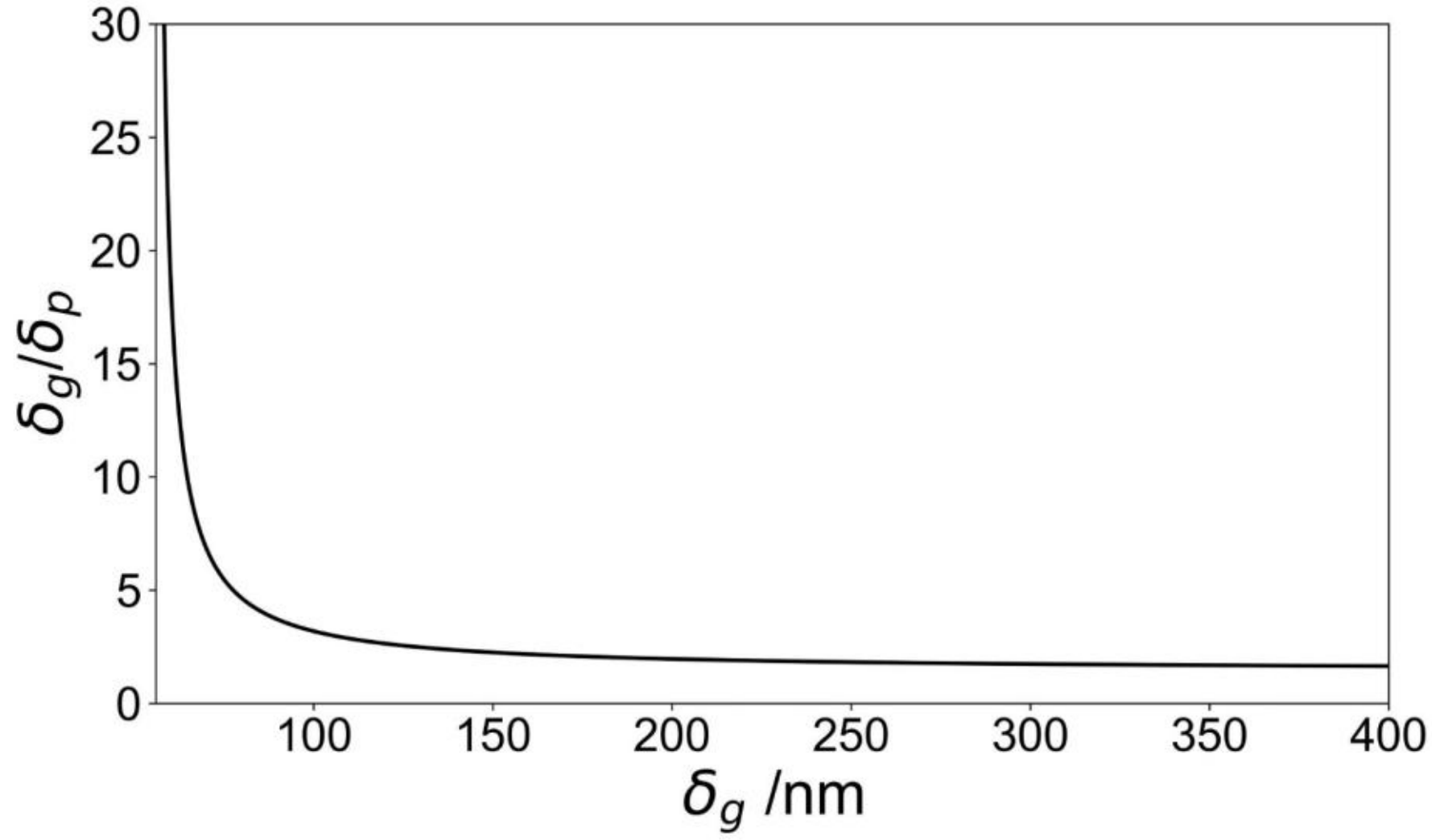

Supplementary Figure 57. Theoretical relationship between the slip length ratio of water-graphene to water-PVDF (δg/δp) and the slip length of water-graphene (δg).

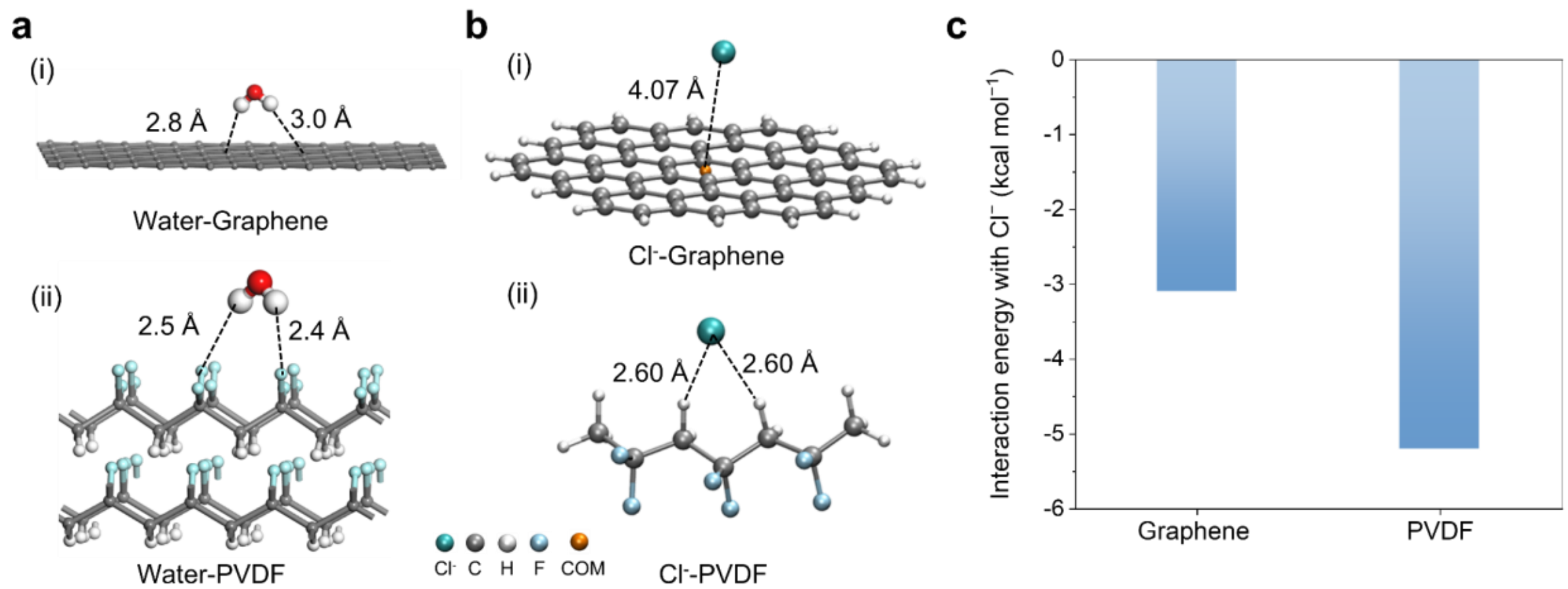

Supplementary Figure 58. Geometrically optimized structures: (a) water adsorption on (i) graphene or (ii) PVDF. (b, i) Cl^--graphene complex and (b, ii) the Cl^--*β*-PVDF complex. The centre of mass (COM) is indicated for the six-carbon ring in graphene. The distances between the Cl^- and the COMs, along with several of the shortest atomic distances between the water and Cl^- and PVDF, are denoted by the dotted lines. The distance unit is given in Å. (c) The interaction energy between Cl^- ion and graphene and *β*-PVDF.

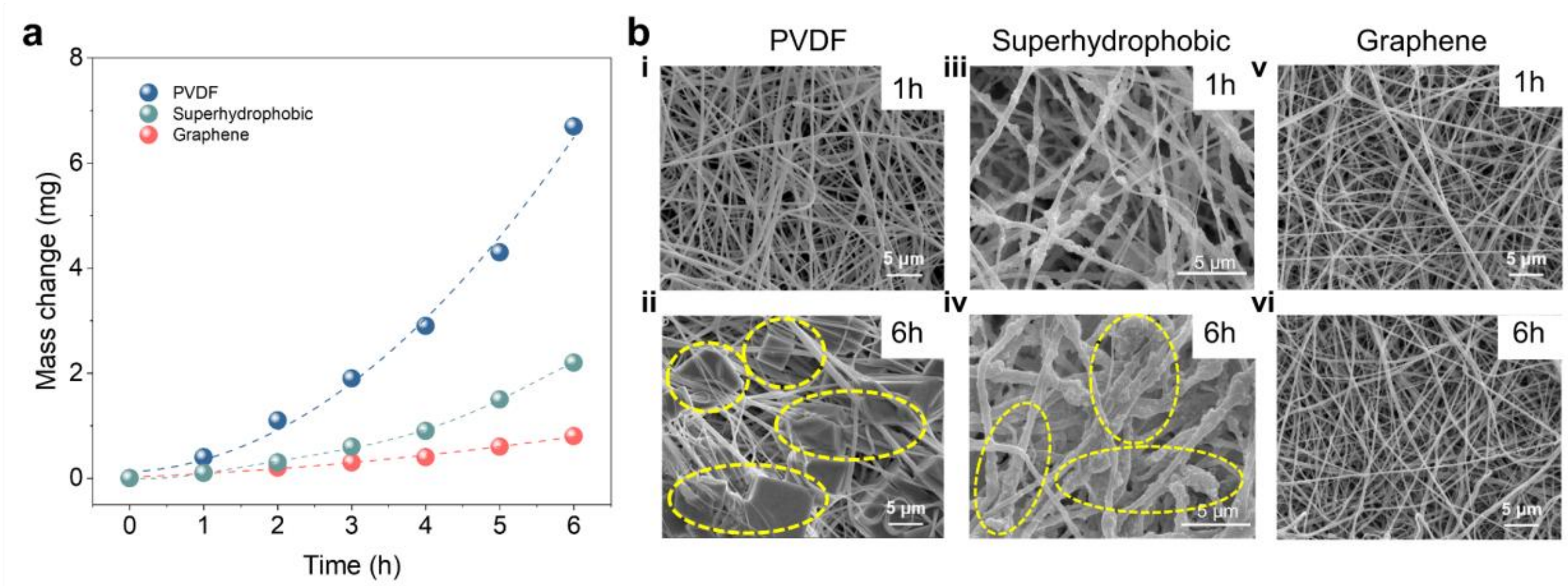

Supplementary Figure 59. (a) Mass change for graphene, PVDF, and superhydrophobic membranes immersing in high salinity water (salt concentration: 150g L^{-1}). (b) SEM surface images of (i) the PVDF, (ii) superhydrophobic and (iii) the graphene membrane after 1 h and 6 h of salt adsorption test. Yellow circles represent the salt crystals. No salt deposition is observed for graphene membrane, in contrast to the superhydrophobic and PVDF membranes.

Supplementary Table 1. Comparison of hydrophobic interfacial evaporation desalination performance of the graphene membrane and state-of-the-art membranes reported in the literature using a feed concentration of ≥50 g L^{-1} NaCl solution.

Membrane	NaCl concentration (g L^{-1})	Feed temperature (°C)	Permeate temperature (°C)	Flux (L m^{-2} h^{-1})	Permeance (10^{-7} kg m^{-2} s^{-1} Pa^{-1})	Salt rejection (%)	Duration (h)	Ref.
FAS GF	58.5	60	20	22	3.58	>99.99	8	[34]
rGO@PVDF-HFP	60	75	25	21	1.70	> 99.97	42	[35]
TF200	65	80	20	48	3.06	> 99.99	100	[36]
CVD graphene-based	70	60	20	~50	8.22	> 99.99	72	[37]
CNTs@PcH	70	60	20	24	3.94	> 99.99	/	[38]
PDTS-Plasma-PTFE	70	50	20	14.7	4.22	/	3	[39]
E-CNT	70	60	20	34.23	5.63	/	/	[40]
PVDF-NA	70	70	20	22	2.20	> 99.99	40	[41]
CNTs@PcH/PET	70	60	20	~16.5	2.71	/	/	[42]
Modified PVDF	100	53	20	9.2	2.29	> 99.99	150	[43]
LF-NC	100	60	20	10	1.68	> 99.99	120	[44]
Modified omniphobic PAN	100	60	20	16	2.63	/	/	[45]
DHPVC-g-PEA	100	65	20	14	1.83	> 99.9	/	[46]
PVDF hollow fiber	100	70	20	~12.5	1.28	/	/	[47]
PCAH	100	60	10	5	0.79	> 99.99	40	[48]
PVDF-P-T	100	60	20	19.5	3.44	> 99.9	/	[49]
Ti-CNFs/PVDF	135	70	20	24.5	2.59	> 99.9	72	[50]
PS/PAN	150	60	20	14.3	2.52	/	/	[51]
PP	150	60	20	11	1.94	/	/	[52]
PS/PC	150	60	20	23	4.06	> 99.9	48	[53]
Cold-pressed PMMA	150	60	20	35.94	6.34	> 99.99	24	[54]
SiC	150	78	/	14	1.02	> 99.9	48	[55]
PVDF	150	60	20	29.5	/	>99.99	90	This
Graphene	150	60	20	50.9	8.98	>99.99	1000	work

FAS: Fluoroalkylsilane; GF: Glass fiber; PVDF-HFP: Poly(vinylidene fluoride-co-hexafluoropropylene); TF-200: Commercial PTFE membrane; CNTs: Carbon nanotubes; PcH: Polyvinylidene fluoride-co-hexafluoropropylene; PDTS: 1H,1H,2H,2H-perfluorodecyl-triethoxysilane; PTFE: Polytetrafluoroethylene; PP: Polypropylene; NA: Nucleation agent; PET: Polyethylene glycol terephthalate; LF: LUMIFLON™ LF-200, a fluoroethylene vinyl ether polymer; NC: Nitrocellulose; PAN: Polyacrylonitrile; DHPVC-g-PEA: A PVC homopolymer along with a poly ethyl acrylate (PEA) monomer; PCAH: A composite membrane containing Nafion, RO membrane and PTFE support. PS: Polystyrene; PC: Polycarbonate; PMMA: Poly (methyl methacrylate); SiC: silicon carbide.

Supplementary Table 2. Comparison of hydrophobic interfacial evaporation desalination performance of the graphene membrane and state-of-the-art membranes reported in the literature using a feed concentration of 35 g L^{-1} NaCl solution.

Membrane	NaCl concentration (g L^{-1})	Feed temperature (°C)	Permeate temperature (°C)	Flux (L m^{-2} h^{-1})	Salt rejection (%)	Ref.
F-POSS/PVDF-HFP	35	50	25	8.0	99.90	[56]
PDA-GO/PVDF	35	75	20	11	> 99.90	[57]
Commercial PVDF	35	60	20	11	> 99.90	[58]
CNT	35	65	5	12	99.00	[59]
GO	35	60	20	14	99.99	[60]
PVDF-HFP-SiA	35	60	20	15	> 99.99	[61]
Commercial PVDF	35	60	20	22	> 99.98	[62]
FS-SiO_2-PVDF/PVDF	35	60	20	25	> 99.99	[63]
PDMS/silica/PVDF	35	60	20	28	>99.99	[64]
PH	35	60	20	29	99.99	[62]
CNT/PVDF-HFP	35	60	20	30	> 99.99	[65]
DPA-Ag/PVDF	35	60	20	32	> 99.99	[58]
PVDF	35	80	20	36	> 99.98	[66]
Ti-CNFs/PVDF	35	65	15	37	>99.99	[50]
PTFE/POSS	35	60	20	38	> 99.99	[67]
PVDF	35	70	20	41	99.00	[68]
F-SiO_2-PDA/PEI@PI	35	80	20	50	>99.99	[69]
PS	35	70	20	51	>99.99	[70]
Matrimid	~35	80	20	58	99.99	[71]
(GO-NBA)-PVDF	35	80	20	62	> 99.90	[72]
PVDF-PTFE hollow fiber	35	60	20	28	> 99.99	[73]
PVDF/TBAHP/PS	35	60	20	50	> 99.90	[74]
TiO_2-PVDF-HFP	35	60	20	38	> 99.99	[75]
MOF@GO-PDMS	35	60	20	22	>99.99	[76]
PVDF-Ag-F	35	60	20	23	99.90	[77]
TiO_2-PH	35	60	20	38	>99.99	[78]
OTS-silica-PVDF	35	60	20	21	>99.99	[79]
PTFE/PET Janus	35	65	25	19	99.90	[80]
PVDF-Teflon AF	35	60	10	28	99.90	[81]
F-TiO_2 nanofiber	35	80	20	12	> 99.99	[82]
PVDF-CNT	35	80	20	28	> 99.9	[83]
PS/PET	35	80	20	31	>99.99	[84]
PVDF-PTFE-PPFDA	35	50	20	13	99.99	[85]
Graphene	35	60	20	61	>99.99	This work
	35	80	20	105	>99.99	

PVDF: Polyvinylidene fluoride; POSS: Polyhedral oligomeric silsesquioxane; HFP: hexafluoropropylene; PDA: Polydopamine; GO: Graphene oxide; CNT: Carbon Nanotube; SiA: silica aerogel; FS: α,ω-triethoxysilane-terminated perfluoropolyether, Fluorolink S10; PDMS: Polydimethylsiloxane; PH: Poly(vinylidene fluoride-co-

hexafluoropropylene); DPA: Dopamine; CNFs: Carbon nano fibers; PTFE: Polytetrafluoroethylene; PEI: Polyethyleneimine; PI: Polyimide; PS: Polystyrene; TBAHP: Tetrabutylammonium hexafluorophosphate; OTS: Octadecyltri-chlorosilane; PET: Polyethylene glycol terephthalate; PI: Polyimide; PPFDA: Poly (1H,1H,2H,2H-perfluorodecyl acrylate).

Supplementary Table 3. Rejection of graphene membrane and conventional RO membranes for boron contaminant, boron-sodium mixture, cesium, cesium-sodium mixture, and simulated radioactive wastewater system (sodium-cobaltous-cesium-strontium mixture) with feed temperature of 60°C.

**System**	**RO membrane**	**This work**
Boron	30%~50%	>99.8%
Boron-NaCl		>99. 6%
Cesium (Cs)	95%~97%	>99. 99%
Cs-NaCl		>99. 98%
Co-Cs-Sr-NaCl		>99. 98%

Supplementary Table 4. Comparison of water-in-oil emulsion separation performance of the graphene membrane and state-of-the-art membranes reported in the literature.

Membrane	System	Concentration	Permeance ($L\ m^{-2}\ h^{-1}\ bar^{-1}$)	Separation efficiency (%)	Ref.
Modified Polyketone	Water-Hexane emulsion	1:99 (v:v)	~14500	~99	[86]
TA/DETA co-deposition	Water-Hexane emulsion	1:99 (v:v)	~12000	~99	[87]
PP-g-SiO_2 NPs/CF_3AZO	Water-Petroleum ether emulsion	1:1 (v:v)	~2427	~96.3	[88]
	Water-Hexane emulsion	1:1 (v:v)	~2653	~93	
PAN@TiO_2/PVDF-HFP@SiO_2	Water-Hexane emulsion	1:99 (v:v)	~2800	~99.3	[89]
F-TiO_2@PPS	Water-Hexane emulsion	1:99 (v:v)	~3889	~98.1	[90]
TPB-DMTP-COF/PAN	Water-Hexane emulsion	1:99 (v:v)	~4574	~97.3	[91]
	Water-Petroleum ether emulsion	1:99 (v:v)	~5645	~97.9	
PES/Fe-Ol	Water-Hexane emulsion	1:99 (v:v)	1275.5	97.4	[92]
Modified PVDF-silica	Water-Petroleum ether emulsion	1:99 (wt:wt)	300	~99.4	[93]
SiC	Water-Hexane emulsion	1000 ppm	~1000	~99	[94]
Al_2O_3-HDTMS	Water-Hexane emulsion	1000 ppm	~2432	~99	[95]
Coprinus comatus-coated	Water-Hexane emulsion	2:98 (v:v)	135	~98.8	[96]
PS-b-P4VP/PAAO	Water-Hexane emulsion	1:100 (v:v)	~1600	99.9	[97]
ZIF-8/P(AN-MA)	Water-Hexane emulsion	1:99 (v:v)	~880	~97.8	[98]
	Water-Petroleum ether emulsion	1:99 (v:v)	~900	~98.3	
Ag@PDA Nylon	Water-Hexane emulsion	1:100 (v:v)	~2073	~99.3	[99]
PFTS-SiO_2/PVDF	Water-Hexane emulsion	1:100 (v:v)	~6800	99	[100]
Polyaniline/SiO_2- PTFE	Water-Hexane emulsion	1:100 (v:v)	~1330	~99	[101]
SiO_2/PVDF + PEI/PDMS	Water-Hexane emulsion	1:24 (v:v)	237.6	99.68	[102]
ZnO NF@SiC	Water-Hexane emulsion	1000 ppm	~1300	~99	[103]
Graphene	Water-Petroleum ether emulsion	1:99 (v:v)	22034	98.7	This work
	Water-Hexane emulsion	1:99 (v:v)	25719	98.7	

TA: Tannic acid; DETA: Diethylenetriamine; CF_3AZO: 7-[(trifluoromethoxyphenylazo)phenoxy]pentanoic acid; PPS: Poly(phenylene sulfide); TPB: 1,3,5-tris(4-aminophenyl)benzene; DMTP: 2,5-dimethoxyterephthalaldehyde; PAAO: Porous Anodic Aluminum Oxide membrane; 4VP: 4-vinyl pyridine; p (AN-MA): Polyacrylonitrile–methyl acrylate.

Supplementary Table 5. Comparison of ethanol/water separation performance of the PDMS@graphene membrane and state-of-the-art membranes reported in the literature.

Membrane	Feed concentration (wt%)	Feed temperature (ºC)	Flux (kg m^{-2} h^{-1})	Separation factor	Ref.
PDMS/PVDF	5	40	0.38	5.7	[104]
PDMS/PVDF	5	40	0.49	5.4	[105]
PDMS/PVDF	5	40	~0.43	~6.4	[106]
PDMS/PVDF	5	40	~0.47	~7.3	[107]
PDMS/PVDF	5	40	~1.35	~7.4	[108]
PDMS/PVDF	5	50	0.80	5.6	[109]
PDMS/PVDF	5	50	2.60	6.7	[110]
PDMS/PVDF	5	50	0.75	5.8	[111]
PDMS/PVDF	5	55	~0.49	4.4	[112]
PDMS/CA	5	40	1.10	8.0	[113]
PDMS/PS	5	60	3.28	7.5	[114]
Corrugated PDMS/PVDF	5	40	3.9	7.8	[115]
PIM-1	5	65	0.83	3.1	[116]
Poly(octhylmethyl siloxane)/PAN	5	50	0.12	4.0	[117]
Polyether-block-amide/PTFE	5	60	3.9	4.9	[118]
Poly(styrene)-block-poly(butadiene)-block-poly(styrene)	10	40	0.03	5.8	[119]
Poly(styrene)-block-poly(butadiene)-block-poly(styrene)	5	50	0.06	3.9	[120]
Poly(vinyl butyral)	7.5	40	0.55	0.7	[121]
PDMS/PVDF	5	60	7.46	7	
PDMS@Graphene	5	40	5.51	7.4	This work
	5	50	7.47	7.1	
	5	60	10.72	6.7	

CA: Cellulose acetate; PDMS-b-PPO: Polydimethylsiloxane-block-polyphenylene oxide; PTFPMS: Poly[(3,3,3-trifluoropropyl)methylsiloxane].

Supplementary Table 6. Comparison of water activation energy during hypersaline desalination process between graphene membrane and reported membranes.

Membrane	E_{water} (kJ mol^{-1})	Ref.
PVDF-PTFE-PPFDA	59.36	[85]
Superhydrophobic PVDF	51.88	[122]
Polysulfone/polydimethylsiloxane	51.84	[123]
PS	50.88	[70]
Ti-CNFs/PVDF	49.55	[50]
PVDF	47.7	[68]
Crosshatched PVDF-HFP nanofiber	47.3	[124]
PS/PET	46.48	[84]
PVDF	46.31	[66]
PE	46.14	[125]
Graphene	37.16	This work

Supplementary Table 7. Comparison of ethanol activation energy during pervaporation recovery of ethanol process between graphene membrane and reported membranes.

Membrane	$E_{ethanol}$ (kJ mol^{-1})	Ref.
PDMS	53.44	[126]
PDMS	43.35	[127]
PDMS	40.38	[128]
PDMS	39.03	[129]
PDMS	37.66	[130]
PDMS	37.22	[131]
PDMS	36.35	[132]
PDMS	36.04	[133]
PDMS	35.55	[134]
PDMS (Graphene)	22.78	This work

References

1. Sauerbrey G. The use of quartz oscillators for weighing thin layers and for microweighing. *Z Fur Phys* 1959; **155**: 206-222.
2. Rodahl M, Höök F, Krozer A *et al.* Quartz crystal microbalance setup for frequency and Q-factor measurements in gaseous and liquid environments. *Rev Sci Instrum* 1995; **66**: 3924-3930.
3. Baker RW, Wijmans JG, Huang Y. Permeability, permeance and selectivity: A preferred way of reporting pervaporation performance data. *J Membr Sci* 2010; **348**: 346-352.
4. Van Der Spoel D, Lindahl E, Hess B *et al.* GROMACS: Fast, flexible, and free. *J Comput Chem* 2005; **26**: 1701-1718.
5. Humphrey W, Dalke A, Schulten K. VMD: Visual molecular dynamics. *J Mol Graph Model* 1996; **14**: 33-38.
6. Tang H, Liu DM, Zhao Y *et al.* Molecular Dynamics Study of the Aggregation Process of Graphene Oxide in Water. *J Phys Chem C* 2015; **119**: 26712-26718.
7. Lu T. Sobtop, Version [1.0(dev3.1)], http://sobereva.com/soft/Sobtop.
8. Liu ZY, Lu T, Chen QX. Intermolecular interaction characteristics of the all-carboatomic ring, cyclo [18] carbon: Focusing on molecular adsorption and stacking. *Carbon* 2021; **171**: 514-523.
9. Zhang J, Lu T. Efficient evaluation of electrostatic potential with computerized optimized code. *Phys Chem Chem Phys* 2021; **23**: 20323-20328.
10. Lu T, Chen FW. Multiwfn: A multifunctional wavefunction analyzer. *J Comput Chem* 2012; **33**: 580-592.
11. Neese F. Software update: The ORCA program system—Version 5.0. *Wires Comput Mol Sci* 2022; **12**: e1606.
12. He HY, Klinowski J, Forster M *et al.* A new structural model for graphite oxide. *Chem Phys Lett* 1998; **287**: 53-56.
13. Berendsen HJC, Postma JPM, van Gunsteren WF *et al.* Molecular dynamics with coupling to an external bath. *J Chem Phys* 1984; **81**: 3684-3690.
14. Hub JS, de Groot BL, van der Spoel D. g_wham—A Free Weighted Histogram Analysis Implementation Including Robust Error and Autocorrelation Estimates. *J Chem Theory Comput* 2010; **6**: 3713-3720.

15. Xiong W, Liu JZ, Ma M *et al.* Strain engineering water transport in graphene nanochannels. *Phys Rev E* 2011; **84**: 056329.
16. González MA, Abascal JLF. The shear viscosity of rigid water models. *J Chem Phys* 2010; **132**.
17. Lovinger AJ, Wang TT. Investigation of the properties of directionally solidified poly (vinylidene fluoride). *Polymer* 1979; **20**: 725-732.
18. Kresse G, Furthmuller J. Efficient iterative schemes for ab initio total-energy calculations using a plane-wave basis set. *Phys Rev B* 1996; **54**: 11169-11186.
19. Kresse G, Joubert D. From ultrasoft pseudopotentials to the projector augmented-wave method. *Phys Rev B* 1999; **59**: 1758-1775.
20. Perdew JP, Burke K, Ernzerhof M. Generalized gradient approximation made simple. *Phys Rev Lett* 1996; **77**: 3865-3868.
21. Prasert K, Sutthibutpong T. Unveiling the Fundamental Mechanisms of Graphene Oxide Selectivity on the Ascorbic Acid, Dopamine, and Uric Acid by Density Functional Theory Calculations and Charge Population Analysis. *Sensors* 2021; **21**: 2773.
22. Song C, Wang JY, Meng ZL *et al.* Density Functional Theory Calculations of the Quantum Capacitance of Graphene Oxide as a Supercapacitor Electrode. *Chemphyschem* 2018; **19**: 1579-1583.
23. Grimme S, Ehrlich S, Goerigk L. Effect of the Damping Function in Dispersion Corrected Density Functional Theory. *J Comput Chem* 2011; **32**: 1456-1465.
24. Monkhorst HJ, Pack JD. Special points for Brillouin-zone integrations. *Phys Rev B* 1976; **13**: 5188-5192.
25. Momma K, Izumi F. VESTA 3 for three-dimensional visualization of crystal, volumetric and morphology data. *J Appl Crystallogr* 2011; **44**: 1272-1276.
26. Kuhne TD, Iannuzzi M, Del Ben M *et al.* CP2K: An electronic structure and molecular dynamics software package - Quickstep: Efficient and accurate electronic structure calculations. *J Chem Phys* 2020; **152**: 194103.
27. VandeVondele J, Hutter J. Gaussian basis sets for accurate calculations on molecular systems in gas and condensed phases. *J Chem Phys* 2007; **127**: 114105.
28. Neese F. The ORCA program system. *Wiley Interdiscip Rev: Comput Mol Sci* 2012; **2**: 73-78.

29. Xie Q, Alibakhshi MA, Jiao SP *et al.* Fast water transport in graphene nanofluidic channels. *Nat Nanotechnol* 2018; **13**: 238-245.
30. Keerthi A, Goutham S, You Y *et al.* Water friction in nanofluidic channels made from two-dimensional crystals. *Nat Commun* 2021; **12**: 3092.
31. Celebi K, Buchheim J, Wyss RM *et al.* Ultimate Permeation Across Atomically Thin Porous Graphene. *Science* 2014; **344**: 289-292.
32. Radha B, Esfandiar A, Wang FC *et al.* Molecular transport through capillaries made with atomic-scale precision. *Nature* 2016; **538**: 222-225.
33. Ohshima H, Takeda S-i. Determination of the Hamaker constant for polymers using their Hansen solubility parameters. *Colloid Polym Sci* 2025; **303**: 805-811.
34. Boo C, Lee J, Elimelech M. Engineering Surface Energy and Nanostructure of Microporous Films for Expanded Membrane Distillation Applications. *Environ Sci Technol* 2016; **50**: 8112-8119.
35. Chen TT, Soroush A, Rahaman MS. Highly Hydrophobic Electrospun Reduced Graphene Oxide/Poly(vinylidene fluoride-co-hexafluoropropylene) Membranes for Use in Membrane Distillation. *Ind Eng Chem Res* 2018; **57**: 14535-14543.
36. Contreras-Martínez J, García-Payo C, Khayet M. Electrospun Nanostructured Membrane Engineering Using Reverse Osmosis Recycled Modules: Membrane Distillation Application. *Nanomaterials* 2021; **11**: 1601.
37. Seo DH, Pineda S, Woo YC *et al.* Anti-fouling graphene-based membranes for effective water desalination. *Nat Commun* 2018; **9**: 683.
38. Tijing LD, Woo YC, Shim WG *et al.* Superhydrophobic nanofiber membrane containing carbon nanotubes for high-performance direct contact membrane distillation. *J Membr Sci* 2016; **502**: 158-170.
39. Giraldo-Mejía H, Quintero YM, Mery F *et al.* Plasma-grafting surface modifications to enhance membrane hydrophobicity for brine membrane distillation. *Desalination* 2023; **567**: 116942.
40. Lee JG, Lee EJ, Jeong S *et al.* Theoretical modeling and experimental validation of transport and separation properties of carbon nanotube electrospun membrane distillation. *J Membr Sci* 2017; **526**: 395-408.

41. Han MY, Liu GH, Fang CJ *et al.* Robust superhydrophobic Poly(vinylidene fluoride) membranes with spherulitic surface morphology against wetting and scaling in membrane distillation. *J Membr Sci* 2024; **708**: 123027.
42. Song JL, Deng Q, Huang MH *et al.* Carbon nanotube enhanced membrane distillation for salty and dyeing wastewater treatment by electrospinning technology. *Environ Res* 2022; **204**: 111892.
43. Hou DY, Christie KSS, Wang K *et al.* Biomimetic superhydrophobic membrane for membrane distillation with robust wetting and fouling resistance. *J Membr Sci* 2020; **599**: 117708.
44. Zhang YJ, Chong JY, Zhao YL *et al.* Facile hydrophobic modification of hydrophilic membranes by fluoropolymer coating for direct contact membrane distillation. *J Membr Sci* 2023; **672**: 121432.
45. Afsari M, Li QY, Karbassiyazdi E *et al.* Electrospun nanofiber composite membranes for geothermal brine treatment with lithium enrichment via membrane distillation. *Chemosphere* 2023; **318**.
46. Hussein SS, Ibrahim SS, Toma MA *et al.* Novel chemical modification of polyvinyl chloride membrane by free radical graft copolymerization for direct contact membrane distillation (DCMD) application. *J Membr Sci* 2020; **611**: 118266.
47. Chen HY, Wu CR, Jia Y *et al.* Comparison of three membrane distillation configurations and seawater desalination by vacuum membrane distillation. *Desalin Water Treat* 2011; **28**: 321-327.
48. Zhao RX, Meng FX, Wu QH *et al.* Ultra-antiwetting Membrane for Hypersaline Water Crystallization in Membrane Distillation. *Environ Sci Technol* 2024; **58**: 14929-14939.
49. Fang CY, Garcia-Rodriguez O, Shang CN *et al.* An omniphobic membrane with macro-corrugation for the treatment of real pharmaceutical wastewater via membrane distillation. *J Membr Sci* 2023; **676**: 121582.
50. Zhang W, Wang Z, Li BA. Omniphobic membrane with nest-like re-entrant structure via electrospraying strategy for robust membrane distillation. *J Membr Sci* 2021; **640**: 119824.
51. Afsari M, Shirazi MMA, Ghorbani AH *et al.* Triple-layer nanofiber membrane with improved energy efficiency for treatment of hypersaline solution via membrane distillation. *J Environ Chem Eng* 2023; **11**: 110638.

52. Eykens L, Reyns T, De Sitter K *et al.* How to select a membrane distillation configuration? Process conditions and membrane influence unraveled. *Desalination* 2016; **399**: 105-115.
53. Bonyadi E, Niknejad AS, Ashtiani FZ *et al.* A well-designed polystyrene/polycarbonate membrane for highly saline water desalination using DCMD process. *Desalination* 2022; **528**: 115604.
54. Niknejad AS, Bazgir S, Kargari A. Desalination by direct contact membrane distillation using a superhydrophobic nanofibrous poly (methyl methacrylate) membrane. *Desalination* 2021; **511**: 115108.
55. Miao K, Song YQ, Guan KC *et al.* Robust hydrophobic ceramic membrane for high-salinity wastewater separation via membrane distillation. *Desalination* 2024; **592**: 118091.
56. Lu C, Su CL, Cao HB *et al.* Nanoparticle-free and self-healing amphiphobic membrane for anti-surfactant-wetting membrane distillation. *J Environ Sci* 2021; **100**: 298-305.
57. Alberto M, Skuse C, Tamaddondar M *et al.* Immobilized graphene oxide-based membranes for improved pore wetting resistance in membrane distillation. *Desalination* 2022; **537**: 115898.
58. Liao Y, Wang R, Fane AG. Engineering superhydrophobic surface on poly(vinylidene fluoride) nanofiber membranes for direct contact membrane distillation. *J Membr Sci* 2013; **440**: 77-87.
59. Dumée LF, Sears K, Schütz J *et al.* Characterization and evaluation of carbon nanotube Bucky-Paper membranes for direct contact membrane distillation. *J Membr Sci* 2010; **351**: 36-43.
60. Mao YY, Huang QB, Meng BC *et al.* Roughness-enhanced hydrophobic graphene oxide membrane for water desalination via membrane distillation. *J Membr Sci* 2020; **611**: 118364.
61. Woo YC, Yao MW, Shim WG *et al.* Co-axially electrospun superhydrophobic nanofiber membranes with 3D-hierarchically structured surface for desalination by long-term membrane distillation. *J Membr Sci* 2021; **623**: 119028.
62. Yao MW, Woo YC, Tijing LD *et al.* Effect of heat-press conditions on electrospun membranes for desalination by direct contact membrane distillation. *Desalination* 2016; **378**: 80-91.
63. Liao Y, Loh CH, Wang R *et al.* Electrospun Superhydrophobic Membranes with Unique Structures for Membrane Distillation. *ACS Appl Mater Interfaces* 2014; **6**: 16035-16048.

64. Liao Y, Zheng GT, Huang JJ *et al.* Development of robust and superhydrophobic membranes to mitigate membrane scaling and fouling in membrane distillation. *J Membr Sci* 2020; **601**: 117962.
65. Tijing LD, Woo YC, Shim WG *et al.* Superhydrophobic nanofiber membrane containing carbon nanotubes for high-performance direct contact membrane distillation. *J Membr Sci* 2016; **502**: 158-170.
66. Francis L, Ghaffour N, Alsaadi AS *et al.* PVDF hollow fiber and nanofiber membranes for fresh water reclamation using membrane distillation. *J Membr Sci* 2014; **49**: 2045-2053.
67. Ju JG, Fejjari K, Cheng Y *et al.* Engineering hierarchically structured superhydrophobic PTFE/POSS nanofibrous membranes for membrane distillation. *Desalination* 2020; **486**: 114481.
68. Tian MM, Yuan S, Decaesstecker F *et al.* One-step fabrication of isotropic poly(vinylidene fluoride) membranes for direct contact membrane distillation (DCMD). *Desalination* 2020; **477**: 114265.
69. Zhu ZG, Liu YR, Hou HQ *et al.* Dual-Bioinspired Design for Constructing Membranes with Superhydrophobicity for Direct Contact Membrane Distillation. *Environ Sci Technol* 2018; **52**: 3027-3036.
70. Li X, Wang C, Yang Y *et al.* Dual-Biomimetic Superhydrophobic Electrospun Polystyrene Nanofibrous Membranes for Membrane Distillation. *ACS Appl Mater Interfaces* 2014; **6**: 2431-2438.
71. Francis L, Maab H, AlSaadi A *et al.* Fabrication of electrospun nanofibrous membranes for membrane distillation application. *Desalin Water Treat* 2013; **51**: 1337-1343.
72. Lu KJ, Zuo J, Chung T-S. Novel PVDF membranes comprising n-butylamine functionalized graphene oxide for direct contact membrane distillation. *J Membr Sci* 2017; **539**: 34-42.
73. Zhao J, Shi L, Loh CH *et al.* Preparation of PVDF/PTFE hollow fiber membranes for direct contact membrane distillation via thermally induced phase separation method. *Desalination* 2018; **430**: 86-97.
74. Li ZJ, Cheng BW, Ju JG *et al.* Development of a novel multi-scale structured superhydrophobic nanofiber membrane with enhanced thermal efficiency and high flux for membrane distillation. *Desalination* 2021; **501**: 114834.

75. Lee EJ, An AK, Hadi P *et al.* Advanced multi-nozzle electrospun functionalized titanium dioxide/polyvinylidene fluoride-co-hexafluoropropylene (TiO_2/PVDF-HFP) composite membranes for direct contact membrane distillation. *J Membr Sci* 2017; **524**: 712-720.
76. Mao YY, Xu JY, Chen HM *et al.* Hydrophobic metal-organic framework@graphene oxide membrane with enhanced water transport for desalination. *J Membr Sci* 2023; **669**: 121324.
77. Liao XJ, Wang YQ, Liao Y *et al.* Effects of different surfactant properties on anti-wetting behaviours of an omniphobic membrane in membrane distillation. *J Membr Sci* 2021; **634**: 119433.
78. Lee EJ, An AK, He T *et al.* Electrospun nanofiber membranes incorporating fluorosilane-coated TiO_2 nanocomposite for direct contact membrane distillation. *J Membr Sci* 2016; **520**: 145-154.
79. Li X, Yu XF, Cheng C *et al.* Electrospun superhydrophobic organic/inorganic composite nanofibrous membranes for membrane distillation. *ACS Appl Mater Interfaces* 2015; **7**: 21919-21930.
80. Zhang JH, Li N, Wang D *et al.* Omniphobic palygorskite coated Janus membrane with enhanced fouling and wetting resistance for direct contact membrane distillation. *Desalination* 2021; **505**: 114986.
81. Chen YML, Lu KJ, Chung T-S. An omniphobic slippery membrane with simultaneous anti-wetting and anti-scaling properties for robust membrane distillation. *J Membr Sci* 2020; **595**: 117572.
82. Fan YF, Chen S, Zhao HM *et al.* Distillation membrane constructed by TiO_2 nanofiber followed by fluorination for excellent water desalination performance. *Desalination* 2017; **405**: 51-58.
83. Yan KK, Jiao L, Lin SS *et al.* Superhydrophobic electrospun nanofiber membrane coated by carbon nanotubes network for membrane distillation. *Desalination* 2018; **437**: 26-33.
84. Ke H, Feldman E, Guzman P *et al.* Electrospun polystyrene nanofibrous membranes for direct contact membrane distillation. *J Membr Sci* 2016; **515**: 86-97.
85. Ghaleni MM, Al Balushi A, Bavarian M *et al.* Omniphobic Hollow Fiber Membranes for Water Recovery and Desalination. *Acs Applied Polymer Materials* 2020; **2**: 3034-3038.

86. Cheng L, Wang DM, Shaikh AR *et al.* Dual Superlyophobic Aliphatic Polyketone Membranes for Highly Efficient Emulsified Oil-Water Separation: Performance and Mechanism. *ACS Appl Mater Interfaces* 2018; **10**: 30860-30870.
87. Zhang X, Liu C, Yang J *et al.* Wettability Switchable Membranes for Separating Both Oil-in-water and water-in-oil emulsions. *J Membr Sci* 2021; **624**: 118976.
88. Chen Z, Xie HY, Li YJ *et al.* Smart light responsive polypropylene membrane switching reversibly between hydrophobicity and hydrophilicity for oily water separation. *J Membr Sci* 2021; **638**: 119704.
89. Cui WY, Fan TY, Li YN *et al.* Robust functional Janus nanofibrous membranes for efficient harsh environmental air filtration and oil/water separation. *J Membr Sci* 2022; **663**: 121018.
90. Yang C, Han N, Han CY *et al.* Design of a Janus F-TiO_2@PPS Porous Membrane with Asymmetric Wettability for Switchable Oil/Water Separation. *ACS Appl Mater Interfaces* 2019; **11**: 22408-22418.
91. Zhang YQ, Tian SW, Sha QK *et al.* Covalent organic framework functionalized smart membranes with under-liquid dual superlyophobicity for efficient separation of oil/water emulsions. *Sci Total Environ* 2023; **904**: 166895.
92. Abuhasheesh YH, Hegab HM, Wadi VS *et al.* Phase inverted hydrophobic polyethersulfone/iron oxide-oleylamine ultrafiltration membranes for efficient water-in-oil emulsion separation. *Chemosphere* 2023; **337**: 139431-139431.
93. Liao Y, Tian M, Wang R. A high-performance and robust membrane with switchable super-wettability for oil/water separation under ultralow pressure. *J Membr Sci* 2017; **543**: 123-132.
94. Wei YB, Xie ZX, Qi H. Superhydrophobic-superoleophilic SiC membranes with micro-nano hierarchical structures for high-efficient water-in-oil emulsion separation. *J Membr Sci* 2020; **601**: 117842.
95. Ding D, Mao HY, Chen XF *et al.* Underwater superoleophobic-underoil superhydrophobic Janus ceramic membrane with its switchable separation in oil/water emulsions. *J Membr Sci* 2018; **565**: 303-310.
96. Long YF, Shen YQ, Tian HF *et al.* Superwettable Coprinus comatus coated membranes used toward the controllable separation of emulsified oil/water mixtures. *J Membr Sci* 2018; **565**: 85-94.

97. Cai YH, Chen DY, Li NJ *et al.* A smart membrane with antifouling capability and switchable oil wettability for high-efficiency oil/water emulsions separation. *J Membr Sci* 2018; **555**: 69-77.
98. Zhang YQ, Tan LL, Han N *et al.* Janus ZIF-8/P(AN-MA) hybrid microfiltration membrane with selected wettability for highly efficient separation of water/oil emulsions. *Sep Purif Technol* 2023; **304**: 122273.
99. Zheng LZ, Li HQ, Lai XJ *et al.* Superwettable Janus nylon membrane for multifunctional emulsion separation. *J Membr Sci* 2022; **642**: 119995.
100. Lin YQ, Salem MS, Zhang L *et al.* Development of Janus membrane with controllable asymmetric wettability for highly -efficient oil/water emulsions separation. *J Membr Sci* 2020; **606**: 118141.
101. Zhang WF, Li XY, Qu RX *et al.* Janus membrane decorated via a versatile immersion-spray route: controllable stabilized oil/water emulsion separation satisfying industrial emission and purification criteria. *J Mater Chem A* 2019; **7**: 4941-4949.
102. Yang H, Wang Y, Fang S *et al.* Janus polyvinylidene fluoride membranes with controllable asymmetric configurations and opposing surface wettability fabricated via nanocasting for emulsion separation. *Colloid Surface A* 2021; **616**: 126120.
103. Wei JJ, Nian P, Wang YX *et al.* Preparation of superhydrophobic-superoleophilic ZnO nanoflower@SiC composite ceramic membranes for water-in-oil emulsion separation. *Sep Purif Technol* 2022; **292**: 121002.
104. Zhu TY, Xu S, Yu F *et al.* ZIF-8@GO composites incorporated polydimethylsiloxane membrane with prominent separation performance for ethanol recovery. *J Membr Sci* 2020; **598**: 117681.
105. Pan Y, Xie R, Xu BM *et al.* Determination of sorption and diffusion for ethanol through superhydrophobic ZIF/PDMS mixed matrix membrane. *Micropor Mesopor Mat* 2021; **320**: 111086.
106. Zhu TY, Yu X, Yi M *et al.* Facile Covalent Crosslinking of Zeolitic Imidazolate Framework/Polydimethylsiloxane Mixed Matrix Membrane for Enhanced Ethanol/Water Separation Performance. *ACS Sustainable Chem Eng* 2020; **8**: 12664-12676.

107. Mao H, Zhen HG, Ahmad A *et al.* Highly selective and robust PDMS mixed matrix membranes by embedding two-dimensional ZIF-L for alcohol permselective pervaporation. *J Membr Sci* 2019; **582**: 307-321.
108. Mao H, Zhen H-G, Ahmad A *et al.* In situ fabrication of MOF nanoparticles in PDMS membrane via interfacial synthesis for enhanced ethanol permselective pervaporation. *Journal of Membrane Science* 2019; **573**: 344-358.
109. Li YB, Wee LH, Martens JA *et al.* ZIF-71 as a potential filler to prepare pervaporation membranes for bio-alcohol recovery. *J Mater Chem A* 2014; **2**: 10034-10040.
110. Claes S, Vandezande P, Mullens S *et al.* Preparation and benchmarking of thin film supported PTMSP-silica pervaporation membranes. *J Membr Sci* 2012; **389**: 265-271.
111. Si ZH, Li GZ, Wang Z *et al.* A Particle-Driven, Ultrafast-Cured Strategy for Tuning the Network Cavity Size of Membranes with Outstanding Pervaporation Performance. *ACS Appl Mater Interfaces* 2020; **12**: 31887-31895.
112. Pan Y, Yu X. Preparation of Zeolitic Imidazolate Framework-91 and its modeling for pervaporation separation of water/ethanol mixtures. *Sep Purif Technol* 2020; **237**: 116330.
113. Fan SQ, Xiao ZY, Li MH. Energy efficient of ethanol recovery in pervaporation membrane bioreactor with mechanical vapor compression eliminating the cold traps. *Bioresour Technol* 2016; **211**: 24-30.
114. Wang R, Shan LL, Zhang GJ *et al.* Multiple sprayed composite membranes with high flux for alcohol permselective pervaporation. *J Membr Sci* 2013; **432**: 33-41.
115. Li JH, Pan Y, Ji WQ *et al.* High-flux corrugated PDMS composite membrane fabricated by using nanofiber substrate. *J Membr Sci* 2022; **647**: 120336.
116. Alberto M, Luque-Alled JM, Gao L *et al.* Enhanced organophilic separations with mixed matrix membranes of polymers of intrinsic microporosity and graphene-like fillers. *J Membr Sci* 2017; **526**: 437-449.
117. Lazarova M, Boesch P, Friedl A. POMS Membrane for Selective Separation of Ethanol from Dilute Alcohol-Aqueous Solutions by Pervaporation. *Separation Science and Technology* 2012; **47**: 1709-1714.
118. Cai P, Li J, Song DY *et al.* Liquid-liquid interface induced high-flux PEBA pervaporation membrane for ethanol recovery. *J Membr Sci* 2023; **668**: 121254.

119. Kamtsikakis A, Delepierre G, Weder C. Cellulose nanocrystals as a tunable nanomaterial for pervaporation membranes with asymmetric transport properties. *J Membr Sci* 2021; **635**: 119473.
120. Santoro S, Galiano F, Jansen JC *et al.* Strategy for scale-up of SBS pervaporation membranes for ethanol recovery from diluted aqueous solutions. *Sep Purif Technol* 2017; **176**: 252-261.
121. Li JJ, Huang M, Wei P *et al.* Comprehensive analysis on anomalous phenomenon of ethanol-soluble poly(vinyl butyral) membrane for ethanol recovery via pervaporation. *AlChE J* 2022; **68**: e17560.
122. Munirasu S, Banat F, Durrani AA *et al.* Intrinsically superhydrophobic PVDF membrane by phase inversion for membrane distillation. *Desalination* 2017; **417**: 77-86.
123. Li X, Garcia-Payo MC, Khayet M *et al.* Superhydrophobic polysulfone/polydimethylsiloxane electrospun nanofibrous membranes for water desalination by direct contact membrane distillation. *J Membr Sci* 2017; **542**: 308-319.
124. Kim S, Heath DE, Kentish SE. Crosshatched nanofibre membranes for direct contact membrane distillation. *Desalination* 2023; **548**: 116277.
125. Zuo J, Bonyadi S, Chung T-S. Exploring the potential of commercial polyethylene membranes for desalination by membrane distillation. *J Membr Sci* 2016; **497**: 239-247.
126. Sun D, Yang QC, Sun HL *et al.* Effects of PES support layer structure on pervaporation performances of PDMS/PES hollow fiber composite membranes. *Desalin Water Treat* 2016; **57**: 9123-9135.
127. Mohammadi T, Aroujalian A, Bakhshi A. Pervaporation of dilute alcoholic mixtures using PDMS membrane. *Chem Eng Sci* 2005; **60**: 1875-1880.
128. He XP, Wang T, Huang JH *et al.* Fabrication and characterization of superhydrophobic PDMS composite membranes for efficient ethanol recovery via pervaporation. *Sep Purif Technol* 2020; **241**: 116675.
129. Rezakazemi M, Shahidi K, Mohammadi T. Synthetic PDMS composite membranes for pervaporation dehydration of ethanol. *Desalin Water Treat* 2015; **54**: 1542-1549.
130. Li L, Xiao ZY, Tan SJ *et al.* Composite PDMS membrane with high flux for the separation of organics from water by pervaporation. *J Membr Sci* 2004; **243**: 177-187.
131. Wei W, Xia SS, Liu GP *et al.* Effects of polydimethylsiloxane (PDMS) molecular weight on performance of PDMS/ceramic composite membranes. *J Membr Sci* 2011; **375**: 334-344.

132. Zhan X, Li JD, Huang JQ *et al.* Enhanced Pervaporation Performance of Multi-layer PDMS/PVDF Composite Membrane for Ethanol Recovery from Aqueous Solution. *Appl Biochem Biotechnol* 2010; **160**: 632-642.

133. Luo Y, Tan SJ, Wang H *et al.* PPMS composite membranes for the concentration of organics from aqueous solutions by pervaporation. *Chem Eng J* 2008; **137**: 496-502.

134. Zhuang Y, Chen WC, Si ZH *et al.* Polydimethylsiloxane membrane formed by thiol-ene photopolymerization for bio-alcohols pervaporation. *Sep Purif Technol* 2024; **329**: 125138.
